# Illustrating papyrus in Ancient Egypt

**DOI:** 10.1038/s41598-023-27761-7

**Published:** 2023-01-10

**Authors:** Pierre-Olivier Autran, Catherine Dejoie, Caroline Dugand, Maeva Gervason, Pierre Bordet, Jean-Louis Hodeau, Michel Anne, Pauline Martinetto

**Affiliations:** 1 European Synchrotron Radiation Facility, 71 avenue des Martyrs, 38000 Grenoble, France; 2 Univ. Grenoble Alpes, CNRS, Institut Néel, 38000 Grenoble, France; 3 Musée Champollion, 45 rue Champollion, 38450 Vif, France

**Keywords:** Materials science, Condensed-matter physics

## Abstract

Illustrated papyruses from Ancient Egypt have survived across millennia, depicting with vivid colors numerous stories and practices from a distant past. We have investigated a series of illustrated papyruses from Champollion’s private collection showing scenes from the Book of the Dead, a document essential to prepare for the afterlife. The nature of the different pigments and their distribution are revealed by combining optical microscopy, Raman spectroscopy, and synchrotron X-ray powder diffraction and fluorescence. The standardized three-step process from the New Kingdom period was used, comprising a preparatory drawing made of red hematite, a coloring step using pigments from the Egyptian palette, and a final black contour drawn with a carbon-based ink. Interestingly, specific pigment mixes were deliberately chosen to obtain different shades. In some parts, the final contour significantly differs from the preliminary drawing, revealing the artist’s creativity. These results enhance our knowledge of illustrative practices in Ancient Egypt.

## Introduction

A large number of written and illustrated papyruses from ancient Egypt have survived through the ages, carrying stories, practices, and numerous details of the everyday life in Egypt several millennia ago. The first occurrence of papyrus used as a support for communication dates from the first dynasty, with the discovery of a blank roll in the tomb of Hemaka (3200 BC)^1^. The first referenced illustrations were found in a series of administrative documents from the 5th and the 6th dynasties, with the addition by the scribe of descriptive images to support the text^2^. Colored illustrations occupying large portions of papyrus documents flourished during the New Kingdom period, giving a new importance to these images with vivid colors, seen as a new way to disseminate information^2^. Among the illustrated papyruses, funerary documents, massively produced from the New Kingdom until the end of the Roman period, occupy a special place. Indeed, owning a “Book of the Dead” was essential to prepare for entering the afterlife, and consequently its confection and final appearance scaled with the social position of the owner^2^.

A highly standardized illustration process was developed during the New Kingdom period and extensively applied to mural paintings^3^. The main steps involve the realization of a preliminary drawing to position the different elements of the illustration, the coloring of these different elements, and the drawing of the final contours. A similar procedure to illustrate papyruses is generally assumed, and only mentioned in a few studies^2^. Specialized craftsmen, different from the scribe writing the main text, were most probably in charge of the illustrations, another indication of standardization. The base pigments reported in the literature include Egyptian blue and Egyptian green (based on cuprorivaite CaCuSi_4_O_10_ and (cupro)wollastonite ((Cu),Ca)_3_Si_3_O_9_), green malachite (Cu_2_CO_3_(OH)_2_), red hematite (α-Fe_2_O_3_), realgar (As_4_S_4_) and cinnabar (HgS), white calcite (CaCO_3_), gypsum (CaSO_4_·2H_2_O), huntite (Mg_3_Ca(CO_3_)_4_) and lead white (a mix of cerussite PbCO_3_ and hydrocerussite (PbCO_3_)_2_·Pb(OH)_2_), yellow orpiment (As_2_S_3_), and black carbon^2,4–10^. Different hues such as pink were generally obtained by mixing the relevant pigments.

**Figure 1:**
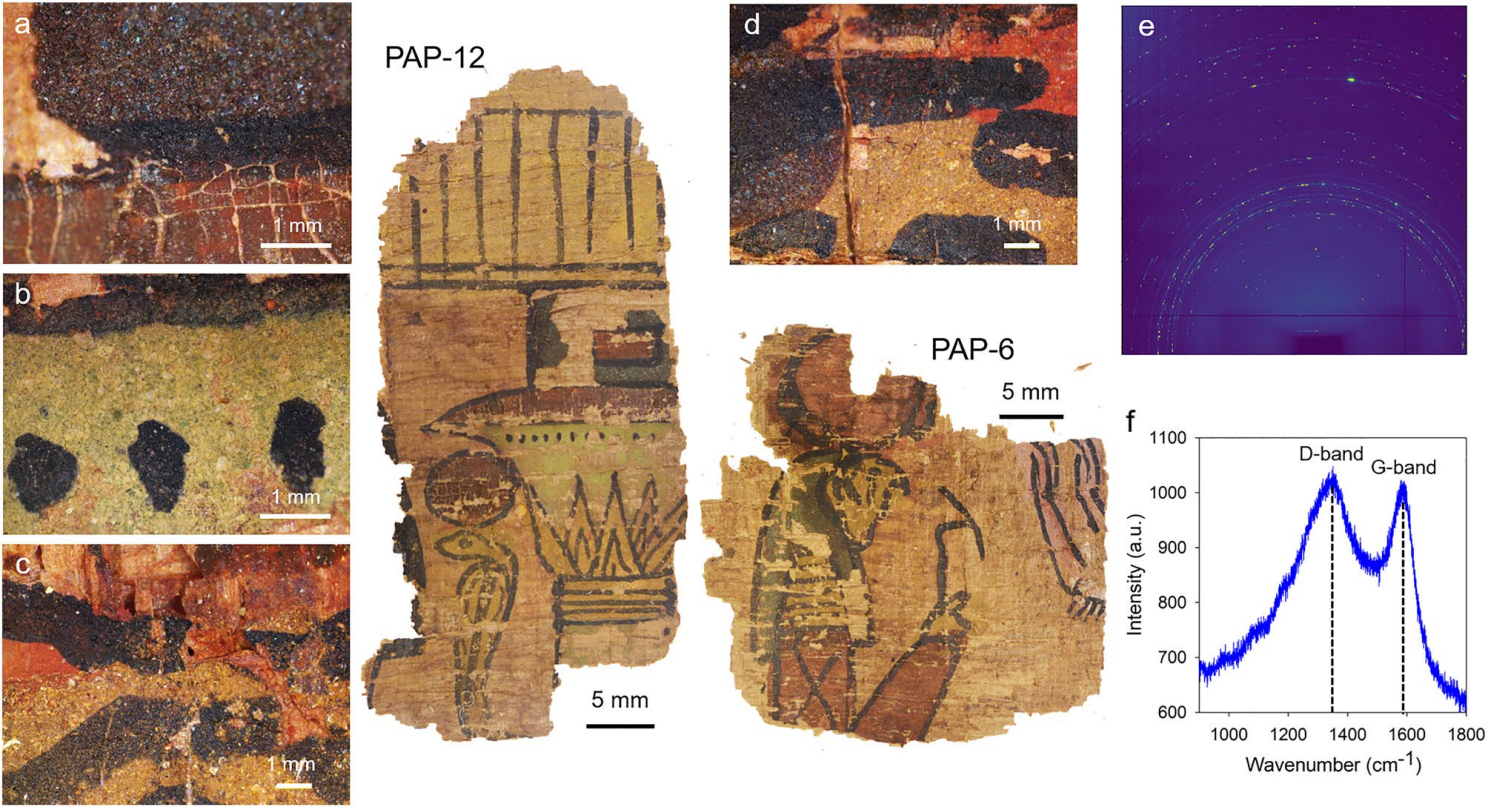
Papyrus fragments PAP-6 and PAP-12 from the Champollion museum (département de l’Isère, Vif, France), showing scenes from the Book of the Dead. (**a**) Detail of the column decoration (PAP-12) obtained from optical microscopy with a blue part, a black line, and a red part where cracks are observed. (**b**) Detail of the column decoration (PAP-12) with dark spots apposed on green paint. (**c**) Region between the red solar disk and the cobra head (PAP-12), with a red part corresponding to the preparatory drawing. (**d**) Detail of the God head (PAP-6) with a yellow paint used to color the face of the God, and a blue one for his cap. The red part corresponds to the preparatory drawing. (**e**) Two-dimensional diffraction image showing spotty diffraction rings. (**f**) Raman spectrum recorded on a black stripe decorating the cloth of the deceased (PAP-6).

The papyrus collection of the Champollion museum (département de l’Isère, Vif, France) contains 280 fragments, the largest ones showing illustrations identified as scenes from the Book of the Dead (Fig. 1). These fragments were stored and preserved by the Champollion-Figeac family over the last two centuries. To the best of our knowledge, no restoration or preservation treatments were ever implemented. Two of these fragments are shown in Fig. 1. On the first fragment (PAP-6), the supposed deceased faces the combined form of the god Ra (solar disk) with Horus (Ra-Hor-Achti), with his hands raised in adoration. The second fragment (PAP-12) represents a cobra (Wadjet) with a solar disk on his head standing in front of a papyriform column (Fig. 1). The colors are typical from the Egyptian palette^8^, with blue, green, red, pink, yellow and white zones clearly identifiable. A black line delineates the contour of the different characters and elements of the illustrations. The stylistic characteristics of these fragments indicate a possible dating from the New Kingdom period.

This access to the papyruses of the Champollion collection is a unique opportunity to gain additional insights into the illustration process of papyrus in Ancient Egypt. One point of particular interest is to understand if the standardized process applied to mural paintings was also commonly used for papyrus illustration, and within this scope, the place left for artistic creativity. To do so, the different colored regions at the surface of the two papyruses shown in Fig. 1 were investigated combining optical microscopy, synchrotron powder X-ray diffraction (XRD) and fluorescence (XRF), and Raman spectroscopy. For each of these regions, the main pigments were identified, and their distribution assessed. Based on such structural and chemical information, the illustration process followed by Ancient Egyptians is revealed, and we show that both standard practices and creativity co-existed.

## Results

The two papyrus fragments PAP-6 and PAP-12 were investigated using optical microscopy, and a series of images recorded on the different colored regions are displayed in Fig. 1. Additional images can be found in Fig. S1. Large blue crystals are seen in the blue regions corresponding to the head of God Ra (Fig. S1A) and to the blue part of the column (Fig. 1a). A few large blue crystals are also present on the black dots of the column decoration (Fig. S1D). The yellow regions are quite heterogeneous, with a mix of yellow, red and white transparent crystals (Fig. 1c,d). The red regions of PAP-6 (the God solar disk and his arm) are more homogeneous, with less noticeable features (Fig. S1B). Similar conclusions are drawn for the red regions of PAP-12 (solar disk of the cobra and column decoration), with the additional presence of large cracks in the red paint (Fig. 1a, Fig. S1E). No particular feature was noticed from the arm of the deceased (pink region) and from its white cloth (Fig. S1C). In the green region toward the top of the column, small green crystals co-exist with some large white/transparent ones (Fig. 1b and Fig. S1D). Finally, in a few specific parts, drawn underneath the colored parts, a red layer is observed (Fig. 1c,d, Fig. S1F).

**Figure 2:**
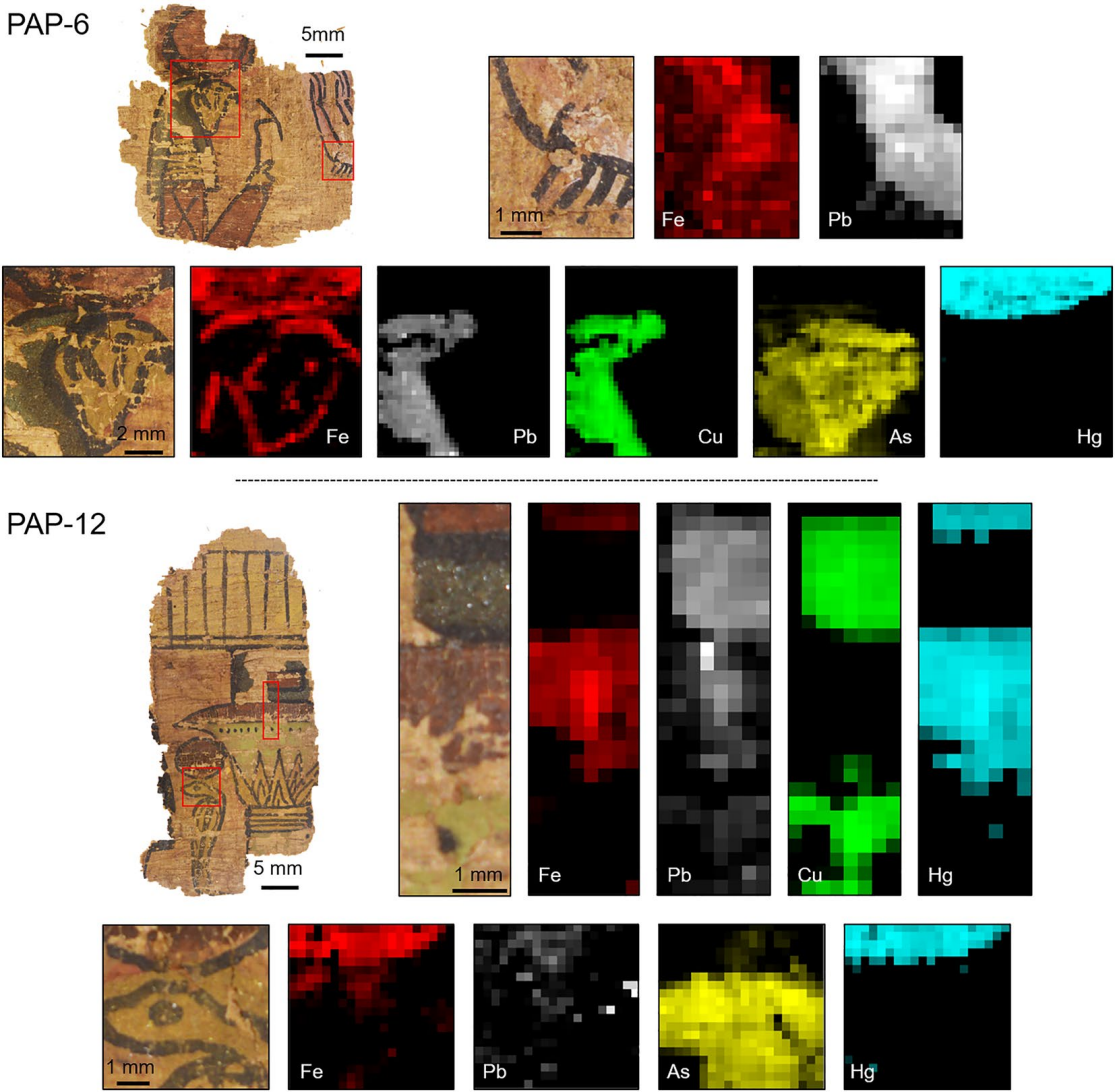
X-ray fluorescence maps recorded on PAP-6 and PAP-12. Data were normalized to the incident X-ray flux, and for each of the maps, the brightest pixel corresponds to the highest amount of the selected chemical element (logarithmic scale, arbitrary units).

Four selected regions from both fragments were investigated by XRF, and corresponding chemical maps are shown in Fig. 2. The fit of the four resulting sum spectra reveals the presence of iron, lead, copper, arsenic, and mercury as main relevant chemical elements (Fig. S2). Iron is found in the red regions corresponding to the two solar disks, the arm of the deceased, and part of the column decoration. Additionally, iron was identified in the region of the cobra head, and on the contour of the God features. Mercury was exclusively found in the red regions of the two solar disks and of the column. Copper appears in blue and green regions (God head and column decoration), and arsenic is present in the yellow parts of the God face and of the cobra. What is interesting to note is the weaker signal of arsenic on the God face, in a part where copper is also present. This is in relation with the presence of a copper-based layer painted on top of an arsenic-based layer, the copper part inducing a fluorescence reabsorption phenomenon. Finally, lead was found on the white cloth of the deceased, and in the blue regions of the God head and of the column.

**Figure 3:**
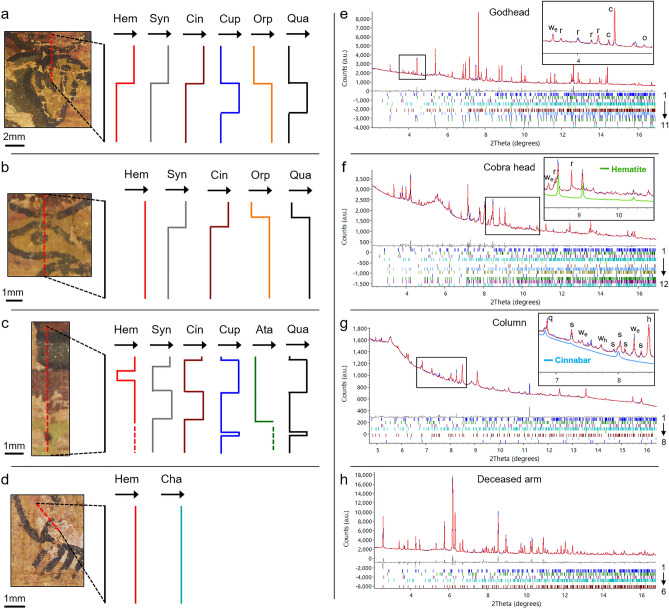
Phase profiles obtained from X-ray diffraction data recorded along the four red lines on (**a**) the Godhead, (**b**) the Cobra head, (**c**) the column, and (**d**) the arm of the deceased. Only the main relevant phases are indicated. The black arrows indicate the absence or presence of each phase, with no information on their relative amount. *Hem* hematite, *Syn* syngenite, *Cin* cinnabar, *Cup* cuprorivaite, *Orp* orpiment, *Qua* quartz, *Ata* atacamite, *Cha* challacolloite. Rietveld and Pawley refinements against diffraction data collected on (**e**) the Godhead, (**f**) the cobra head, (**g**) the column, and (**h**) the arm of the deceased (thin blue line: measured pattern, red line: calculated pattern, grey line: difference, phases 1–4 correspond to the two cellulose phases, weddellite (w_e_) and whewellite (w_h_)). (**e**) Cuprorivaite (c), orpiment (o) and realgar (r) co-exist in the same region of the Godhead (phases 5–11: aphthitalite, realgar, orpiment, cuprorivaite_SC1 and _SC2, quartz_SC1 and _SC2, with SC1 and SC2 representing two large-grain contributions of the same phase, see Fig. S3; R_wp_ 2.82%). (**f**) Hematite from the preparatory drawing is present in the cobra head (phases 5–12: hematite, aphthitalite, realgar, orpiment, quartz_SC, realgar_SC1 and _SC2 (r), orpiment_SC; R_wp_ 1.50%). (**g**) A small amount of cinnabar is present in the red part of the column (phases 5–8: hematite (h), syngenite (s), cinnabar, quartz_SC (q); R_wp_ 0.78%). (**h**) Rietveld refinement against diffraction data collected on the arm of the deceased (phases 5–6: hematite, challacolloite; R_wp_ 4.66%).

Pigments were identified by XRD along four lines crossing the four regions investigated by XRF (Fig. 3a–d). Hematite (α-Fe_2_O_3_), cuprorivaite (CaCuSi_4_O_10_), atacamite (Cu_2_Cl(OH)_3_), orpiment (As_2_S_3_) and realgar (As_4_S_4_), and challacolloite (KPb_2_Cl_5_) were identified in the red, blue, green, yellow and white regions, respectively, in agreement with the results obtained by chemical mapping. In the red regions, along with hematite, syngenite (K_2_Ca(SO_4_)_2_) and gypsum (CaSO_4_·2H_2_O) were also identified. The presence of mercury in some of the red regions, attested by XRF measurements, directed us toward the possible presence of cinnabar (HgS), and indeed, even if difficult to spot, the main diffraction peaks of cinnabar were identified in a few patterns (Fig. 3g). In the pinkish region of the arm of the deceased, hematite was found associated to challacolloite. Hematite was finally identified in the diffraction patterns recorded on the yellow part of the cobra head, in agreement with the presence of iron in the fluorescence map (Fig. 2) and of a red underlying layer in the optical microscopy images (Fig. 1c). In the green region of the column, in addition to atacamite, another copper-based phase, moolooite (Cu(C_2_O_4_)·nH_2_O), was identified. In the yellow regions, aphthitalite, a sulfate of potassium and sodium ((K,Na)_3_Na(SO_4_)_2_), was also found. Finally, quartz (SiO_2_) was identified in the blue regions of PAP-6 and PAP-12, and in the yellow part of the cobra head. A few additional grains of quartz were found randomly dispersed all over the different colored regions (not shown in Fig. 3). A few non-indexed diffraction peaks remain in some patterns.

In addition to the phases identified in the colored regions, a few additional ones, related to the raw papyrus support, are systematically present. The first is cellulose, the main component of papyrus, and two different phases of cellulose were previously identified in the papyrus of the Champollion collection^11^. Then, two calcium oxalate phases, weddellite (Ca(C_2_O_4_)·2H_2_O) and whewellite (Ca(C_2_O_4_)·H_2_O), usually a sign of degradation and possible bacterial activity, were found in all of the regions investigated.

Following phase identification, all the XRD patterns were processed through Rietveld^12^ and Pawley refinements^13^. Rietveld refinement is the method of choice for in-depth structural analysis and/or phase quantification. In such an approach, the pattern calculated from a structural model is compared to the measured one. This requires a precise measure of the reflection intensities through the fulfillment of the powder condition, e.g. the presence of a large number of crystallites randomly oriented in the diffraction volume. In the case of unknown structural models or poorly measured intensities (e.g. with heterogeneous samples), other non-quantitative methods such as Pawley or Le Bail refinements can be implemented, as the reflection intensities are freely adjusted. In the present case, due to a complex microstructure and the cohabitation of small-grain and large-grain phases in the diffraction volume, mixed Rietveld and Pawley refinements were implemented against the XRD patterns collected in the colored regions. Cuprorivaite and quartz are mainly present as large crystals, giving rise to highly spotty diffraction rings (Fig. 1e), and consequently poorly reliable reflection intensities after integration of the 2D diffraction patterns. In such a case, only a Pawley refinement could be performed (Fig. S3). Orpiment and realgar show similar large-crystal diffraction tendency, with nonetheless part of it behaving as a powder. This indicates that a fraction of the yellow pigment has smaller-size crystals, improving, to some extent, the powder average and the reliability of the reflection intensities. Consequently, joint Rietveld and Pawley refinements were carefully implemented for these two phases (Fig. S4).

Among the different colored regions, the red ones, present on both papyruses, appears as the most homogeneous, and with the exception of a few random single-crystals of quartz modelled through Pawley fitting, all the other phases that were identified (cellulose, weddellite, whewellite, hematite, syngenite, gypsum, and cinnabar) behaved as a powder and were included in the Rietveld refinement. The main results obtained after refinement against a total of sixteen diffraction patterns collected on PAP-6 (red solar disk) and on PAP-12 (red part of the column) are shown in Table 1, and the details of the phase quantification are given in Tables S1 and S2. The pigment layer represents less than 10% of the diffraction signal, dominated by cellulose. A slightly higher amount in calcium oxalate (sum of weddellite and whewellite) was found for PAP-6 (average of 5.05% vs. 3.87% for PAP-12), and a change in the weddellite-to-whewellite ratio was observed (average of 1.86 for PAP-6 vs. 1.05 for PAP-12). The hematite-to-syngenite ratio also differs significantly, with an average of 0.29 and of 1.33 for PAP-6 and for PAP-12, respectively.

**Table 1:** Quantitative refinement results from the XRD data collected in the red solar disk region of PAP-6 and in the red region of the column decoration in PAP-12.

Samples		Cellulose (wt%)	Calcium oxalate (wt%)	Ratio Weddelite/Whewellite	Ratio Hematite/Syngenite
PAP-6	Average	92.3	5.05	1.86	0.29
	St. dev	1.4	0.70	0.46	0.12
PAP-12	Average	92.5	3.87	1.05	1.33
	St. dev	2.4	1.07	0.24	0.66

Averages and standard deviations (St. dev.) are calculated from the refinement results of eight patterns in each case (see Tables S1, S2). The weight percent (wt%) in cellulose and in calcium oxalate were obtained after summing the contributions of the two cellulose phases and of weddellite and whewellite, respectively.

A selection of refined diffraction patterns from four different colored regions is shown in Fig. 3. Additional refinements are given in SI. The first diffraction pattern shown in Fig. 3e was recorded in the blue region of the God head (PAP-6) where both Cu and As are simultaneously present in the chemical maps (Fig. 2). As can be seen in Fig. 3e, this is in relation with the concomitant presence of cuprorivaite, orpiment and realgar, in agreement with the results from fluorescence mapping and the presence of superposed painted layers. Another example of pigment layer superposition involving atacamite, moolooite and cuprorivaite from a diffraction pattern recorded on one of the black dots of the column is shown in Fig. S5. The second diffraction pattern displayed in Fig. 3f comes from the yellow part of the cobra head (PAP-12). As explained earlier, orpiment and realgar are described using combined Rietveld and Pawley refinements to take into account both powder and single-crystal (noted SC) contributions, respectively. Hematite is also present (see the inset of Fig. 3f), in agreement with the presence of iron in the fluorescence map (Fig. 2) and of a red underlying layer on the optical microscopy images (Fig. 1c). The third diffraction pattern was recorded in the red region of the column (PAP-12) (Fig. 3g), and hematite, syngenite and cinnabar were included in the fit. The 2θ range where the main reflections of cinnabar are expected is highlighted in the inset. Finally, the last diffraction pattern is from the white cloth of the deceased (PAP-6) where both challacolloite and hematite were identified (Fig. 3h).

The black pigment used to draw the contour lines was investigated through Raman spectroscopy, and the characteristic features of amorphous carbon (D-band at around 1340 cm^−1^, and G-band at around 1590 cm^−1^) were identified in several places over the two fragments (Fig. 1f, Fig. S6). This is in agreement with one of our previous studies, where we showed that the black ink used on the papyrus of the Champollion collection was based on flame carbon^11^. Amorphous carbon was also detected on the series of black dots decorating the column (Fig. 1b), where a few large blue crystals were also observed by optical microscopy (Fig. S1D) and identified as cuprorivaite by XRD (Fig. S5).

## Discussion

Our chemical and structural investigations carried out on the illustrated papyrus of the Champollion collection was a unique opportunity to clarify the illustration process implemented by Ancient Egyptians. First of all, we show that the illustration sequence involves three main steps. The first step corresponds to a preparatory drawing, made of red hematite (Fig. 3f). Part of it can be directly seen on the pictures obtained from optical microscopy (Fig. 1, Fig. S1), and its presence under the pigment layers is also attested by chemical mapping (Fig. 2). Then, the different colors and pigments were apposed, and, from what we observed, a specific order was applied, with the lighter tones first, and the darker shades last. This can be seen on the God head where the face of the God was first painted in yellow with a paint made of orpiment and realgar. As revealed by X-ray fluorescence and by X-ray diffraction, a blue layer, made of cuprorivaite, was added on top of it, to represent the cap of the God (Figs. 2, 3a,e). Another example of such a color superposition is found on the column (PAP-12), where a mix of carbon black and of blue cuprorivaite was used to make the decorative dots, apposed on top of the lighter green part (Fig. S5). Finally, following the coloring process, the contour line made of black carbon was drawn, giving their final appearance to the illustrated scenes. When looking at the features of the God head, the contour line does not strictly follow the preliminary drawings (Fig. 2), suggesting that the craftsman in charge of the final drawing freely adjusted the first model. The three-step process we highlight here—red preparatory drawing, coloring phase, and final black contour—clearly indicates that the standardized three-step process applied to mural paintings at the New Kingdom period was undoubtedly transposed to papyrus illustration.

The nature and the microstructure of the main pigments used on the Champollion papyruses underline further the standardization of the illustration process. Indeed, blue color comes from cuprorivaite (e.g. Egyptian Blue), yellow from orpiment and realgar, red from hematite and cinnabar, with all these pigments described in the literature as being part of the Egyptian palette^2,4,6–8^. What we show in addition is that the microstructure of these three groups of pigments does not significantly change over the two papyrus fragments, with large crystals of cuprorivaite in all of the blue regions (Figs. S1, S3), the co-existence of larger crystallites of orpiment and realgar with a finer fraction in all of the yellow ones (Figs. S1, S4), and the presence of hematite mainly as a fine powder. This indicates that the raw pigments were obtained from standardized sources, following strict synthetic procedure as in the case of cuprorivaite^1^, or grinding protocols for the mineral-based pigments. Concerning the green and white regions where less standard pigments were found, however, it was shown that atacamite and challacolloite can result from the degradation of green malachite and of lead white, respectively, two well-known pigments also used in Ancient Egypt and in agreement with the Egyptian palette^7,10^.

The use of lead white (and also of cinnabar) opens the question of the dating of the papyrus of the Champollion collection, as these two pigments have been mainly found on papyrus or artifacts dated from the Ptolemaic period^7^. More rigorous dating techniques (e.g. carbon dating of the papyrus support) should be carried out, but such destructive methods cannot be implemented in the present case. Nevertheless, the presence of these pigments in the colored illustrations of the two papyrus fragments of the Champollion collection strongly suggest a later dating than first guessed from stylistic considerations.

As just demonstrated, if the choice of the raw pigments mainly results from standardized practices, on the other hand, we also show that their use in a mix and in association with other compounds was freely adjusted depending on the illustrative element to color. For example, the yellow paint used to color the face of the God (PAP-6) is mainly based on orpiment and realgar (Fig. 3a). On the other hand, the yellow pigment used to color the cobra head (PAP-12) was probably “diluted” with quartz, as shown by the almost systematic presence of this phase over that region (Fig. 3b). The red color constitutes another striking example, of which at least three instances were found on PAP-6, and two on PAP-12. The first instance corresponds to the red color used for the preparatory drawing, for which a red ink mainly based on hematite was used. The second instance of red was used to color the two solar disks of the God and of the cobra, and also part of the column decoration. In addition to hematite, a small amount of cinnabar was found, with as well a certain amount of gypsum and of syngenite, the latter resulting from the degradation of gypsum^14^. The hematite-to-syngenite ratio calculated for the red regions of PAP-6 and PAP-12 (Table 1) indicates that different proportions of gypsum were used. Combining hematite, cinnabar and gypsum, and adjusting their relative amount, clearly result from a choice of the craftsman to obtain specific red shades. Finally, the third instance of red corresponds to the arm of the deceased, where hematite and challacolloite were identified. This time, red hematite is mixed with a lead-based white pigment to obtain a different hue and a more pinkish color. Again, this emphasizes the careful choice of the pigment mix to paint the skin of the arm with a dedicated reddish tone. We conclude that the standardization guiding the illustration process and the raw pigment nature did not extend to the paint preparation in terms of pigment mixing, and so, the final choice of the color shades was left to the craftsman.

In order to be used as ink or paint, pigments in Ancient Egypt were generally mixed with an organic binder, typically Arabic gum or animal glue, and with water^4^. In addition, a drier could be added, as shown in previous studies of papyruses from the Greco-Roman period^15,16^. Using Raman spectroscopy, we failed to isolate any signal related to the organic content that may have survived over time. However, the characteristic “cracking” pattern resulting from the drying of an organic binder is clearly visible on both the red solar disk of the cobra and the red part of the column decoration (PAP-12, Fig. 1a, Fig. S1E). In addition, the presence of lead was noticed in the blue regions of the God head (PAP-6) and of the column (PAP-12) (Fig. 2), and we attribute it to the use of a lead-based drier. The use of litharge (PbO) as drier was reported quite early on by Galen (second century AD) and by Marcellus (fourth century AD)^17^, and the presence of lead phosphate, lead sulfate, and lead carboxylate identified in inks from the Roman period were also attributed to the use of driers^15,16^. The presence of a lead-based drier specifically in those blue regions may be related to the microstructure of cuprorivaite, present as large crystals (Fig. 1, Figs. S1, S3), and so requiring the additional use of a drier for better adherence. We highlight here that Ancient Egyptians adjusted the recipe of the paint depending on the pigment chosen in order to obtain optimum adherence properties.

Finally, we could only note the degradation process affecting most of the colored regions. As mentioned in the previous lines, gypsum degraded into syngenite, lead white into challacolloite, and malachite into atacamite and moolooite. Aphthitalite, found in all of the yellow regions, also results from a degradation process, and this will be the topic of a dedicated publication. The systematic presence of calcium oxalates (weddellite and whewellite) all over the two papyrus fragments attests the importance of the general degradation process and the possible role of microorganism activity. We observed that a larger amount of calcium oxalate is present in the red regions of PAP-6, with also a different weddellite-to-whewellite ratio (Table 1). This suggests that the two fragments may have been produced in a different period of time and/or were exposed to a different environment. The additional use of a specific binder in the red regions of PAP-12 also suggests that these two fragments of the Champollion collection may not belong to the same set or the same Book of the Dead.

## Conclusion

Combining optical microscopy, Raman spectroscopy and synchrotron X-ray diffraction and fluorescence, we investigated the nature and the structural properties of the pigments used to illustrate ancient papyruses from the Champollion collection, in relation with the illustration technique deployed by Ancient Egyptians. Most of the pigments identified are part of the known Egyptian palette, with Egyptian blue made of cuprorivaite, malachite to color green parts, hematite and cinnabar for red elements, orpiment and realgar for yellow ones, lead white for white parts, and carbon black to draw the contour line. A few phases, such as challacolloite, atacamite, moolooite or syngenite correspond to degradation phases, and their presence will be the topic of a forthcoming publication. We show that the illustration sequence applied to mural paintings was transposed to papyrus illustration, with a preparatory drawing, a coloring process, and finally the drawing of the final contour. Besides the classic nature of the pigments and the highly standardized illustration technique, we highlight the free and careful choices made by Egyptian craftsmen in the coloring of the illustrations, with specific pigments and mix of pigments to obtain various shades, adapting to the scene and decorative elements to be colored. The use of adjuvants such as driers to improve the adherence for particular color should also be mentioned. The hand of the artist and his creativity emerge at the last step, when apposing the black contour that gives its final force to the illustration. We believe that these results improve our knowledge on the illustration techniques used to massively produce illustrated “Book of the Dead” in Ancient Egypt, and highlight this subtle balance between standardization and creativity, craftsman and artistic work.

## Methods

Optical microscopy measurements were carried out using a Nikon Eclipse LV100ND optical microscope. At each measured point, a series of images were recorded varying the focal length, a way to reduce non-flat surface effects.

Raman spectroscopy experiments were carried out using a Jobin Yvon T6400 spectrometer using a 633 nm incident radiation wavelength. Spectra were recorded for a counting time of 20–40 s, and a laser power of 1mW.

Synchrotron XRD and XRF experiments were carried out at the ID22 beamline of the European Synchrotron Radiation Facility (ESRF, Grenoble, France). XRD data were collected using a Perkin Elmer XRD 1611CP3 detector positioned at a distance of 1400 mm from the sample with a beam size of 1 mm × 0.1 mm (h × v) and at a wavelength of 0.4009 Å. The wavelength and the detector parameters were calibrated using a diffraction pattern collected on a silicon NIST 640c sample. Papyrus fragments were positioned in the beam using dedicated 3D-printed sample holders^11^. At each measurement points, 100 diffraction patterns were recorded (1 s per pattern), before averaging. Azimuthal integration and Pawley and Rietveld refinements were carried out using the PyFAI^18^ library and the Topas software^19^, respectively. X-ray fluorescence spectra were recorded with a Hitachi Vortex 90EX SDD, and corresponding data processing carried out using the PyMca software^20^.

## Supplementary Information

Supplementary Information.

## Data Availability

Data are available from the corresponding authors upon reasonable request.
