# Supplementary material for: Illustrating papyrus in Ancient Egypt
Source: Sci Rep. 2023 Jan 10;13:524. doi: 10.1038/s41598-023-27761-7 (PMC9832040; doi:10.1038/s41598-023-27761-7)
Supplement: Supplementary file 1 — Supplementary Information. [file 41598_2023_27761_MOESM1_ESM.pdf]

**Supplementary information**

# Illustrating papyrus in Ancient Egypt

Pierre-Olivier Autran[1,2], Catherine Dejoie[1,*], Caroline Dugand[3], Maeva Gervason[3], Pierre Bordet[2], Jean-Louis Hodeau[2], Michel Anne[2], Pauline Martinetto[2,*]

[1]European Synchrotron Radiation Facility, 71 avenue des Martyrs, 38000 Grenoble, France
[2]Univ. Grenoble Alpes, CNRS, Institut Néel, 38000 Grenoble, France
[3]Musée Champollion, 45 rue Champollion, 38450 Vif, France

*Corresponding authors: catherine.dejoie@esrf.fr; pauline.martinetto@neel.cnrs.fr

**Table S1.** Quantitative analysis obtained after refinement against the diffraction data collected in the red solar disk region of PAP-6 (see Fig. S7). The total weight percent in cellulose (Sum Cellu.) and in calcium oxalate (Sum WW) were obtained after summing the contributions of the two cellulose phases (P1 and P2) and of weddellite (Wedd) and whewellite (Whe), respectively. The value obtained for gypsum, if present, was added to the syngenite (Syn) one. Cin: cinnabar; Ratio W/W: ratio weddellite/whewellite; Ratio H/S: ratio hematite/syngenite; St. dev.: standard deviation.

| Pattern no. | P1 | P2 | Wedd | Whe | Hem | Syn | Cin | Sum Cellu. | Sum WW | Ratio W/W | Ratio H/S |
|---|---|---|---|---|---|---|---|---|---|---|---|
| G-6785 | 87.9(1) | 3.42(6) | 4.06(5) | 2.01(4) | 0.63(1) | 1.93(6) | 0.068(4) | 91.3 | 6.08 | 2.02 | 0.33 |
| G-7285 | 89.9(2) | 3.50(9) | 3.24(7) | 1.70(5) | 0.37(1) | 1.20(8) | 0.059(5) | 93.4 | 4.94 | 1.90 | 0.31 |
| G-7535 | 90.8(1) | 2.40(5) | 2.91(4) | 1.55(5) | 0.44(1) | 1.79(4) | 0.070(2) | 93.2 | 4.46 | 1.88 | 0.25 |
| G-7785 | 88.8(2) | 3.63(9) | 3.63(6) | 1.53(5) | 0.47(1) | 1.76(7) | 0.142(5) | 92.5 | 5.16 | 2.37 | 0.27 |
| G-8035 | 87.4(2) | 4.9(1) | 2.87(7) | 1.65(5) | 0.24(1) | 2.96(8) | 0.012(5) | 92.3 | 4.53 | 1.74 | 0.08 |
| G-8285 | 90.3(1) | 4.39(9) | 3.00(5) | 1.20(4) | 0.21(1) | 0.91(6) | 0.024(3) | 94.7 | 4.20 | 2.49 | 0.23 |
| G-8535 | 85.7(2) | 4.82(9) | 3.47(6) | 2.59(5) | 0.92(2) | 2.43(5) | 0.069(5) | 90.5 | 6.07 | 1.34 | 0.38 |
| G-8785 | 86.5(1) | 4.17(9) | 2.65(5) | 2.33(5) | 1.44(2) | 2.83(6) | 0.058(4) | 90.7 | 4.98 | 1.14 | 0.51 |
| Average | 88.4 | 3.90 | 3.23 | 1.82 | 0.59 | 1.98 | 0.06 | 92.3 | 5.05 | 1.86 | 0.29 |
| St. dev. | 1.9 | 0.83 | 0.47 | 0.46 | 0.41 | 0.73 | 0.04 | 1.4 | 0.70 | 0.46 | 0.12 |

**Table S2.** Quantitative analysis obtained after refinement against the diffraction data collected in the red region of the column decoration of PAP-12 (see Fig. S7). The total weight percent in cellulose (Sum Cellu.) and in calcium oxalate (Sum WW) were obtained after summing the contributions of the two cellulose phases (P1 and P2) and of weddellite (Wedd) and whewellite (Whe), respectively. The value obtained for gypsum, if present, was added to the syngenite (Syn) one. Cin: cinnabar; Ratio W/W: ratio weddellite/whewellite; Ratio H/S: ratio hematite/syngenite; St. dev.: standard deviation.

| Pattern no. | P1 | P2 | Wedd | Whe | Hem | Syn | Cin | Sum Cellu. | Sum WW | Ratio W/W | Ratio H/S |
|---|---|---|---|---|---|---|---|---|---|---|---|
| C-22750 | 90.8(1) | 6.63(8) | 0.87(2) | 0.72(4) | 0.67(1) | 0.30(2) | 0.052(4) | 97.4 | 1.59 | 1.22 | 2.22 |
| C-20250 | 84.6(3) | 7.1(2) | 1.58(6) | 2.75(8) | 2.66(5) | 1.06(3) | 0.238(9) | 91.7 | 4.33 | 0.57 | 2.52 |
| C-20000 | 83.2(3) | 7.1(1) | 2.26(6) | 2.50(7) | 2.39(4) | 2.39(8) | 0.174(7) | 90.3 | 4.76 | 0.90 | 1.00 |
| C-19750 | 83.9(4) | 6.7(2) | 2.3(1) | 2.1(1) | 2.42(5) | 2.42(8) | 0.092(8) | 90.6 | 4.46 | 1.08 | 1.00 |
| C-19500 | 85.2(2) | 6.9(1) | 2.30(8) | 1.67(7) | 2.06(3) | 1.72(6) | 0.095(5) | 92.1 | 3.98 | 1.38 | 1.20 |
| C-19250 | 83.8(3) | 8.2(2) | 2.19(6) | 1.91(7) | 1.91(4) | 1.90(6) | 0.105(5) | 92.0 | 4.10 | 1.15 | 1.01 |
| C-19000 | 82.4(3) | 8.8(2) | 2.53(6) | 2.20(7) | 1.90(3) | 2.11(5) | 0.047(4) | 91.2 | 4.72 | 1.15 | 0.90 |
| C-18750 | 87.4(2) | 7.3(1) | 1.47(7) | 1.57(8) | 0.95(2) | 1.23(5) | 0.037(3) | 94.7 | 3.04 | 0.94 | 0.77 |
| Average | 85.2 | 7.4 | 1.94 | 1.93 | 1.87 | 1.64 | 0.11 | 92.5 | 3.87 | 1.05 | 1.33 |
| St. dev. | 2.7 | 0.8 | 0.57 | 0.63 | 0.71 | 0.73 | 0.07 | 2.4 | 1.07 | 0.24 | 0.66 |

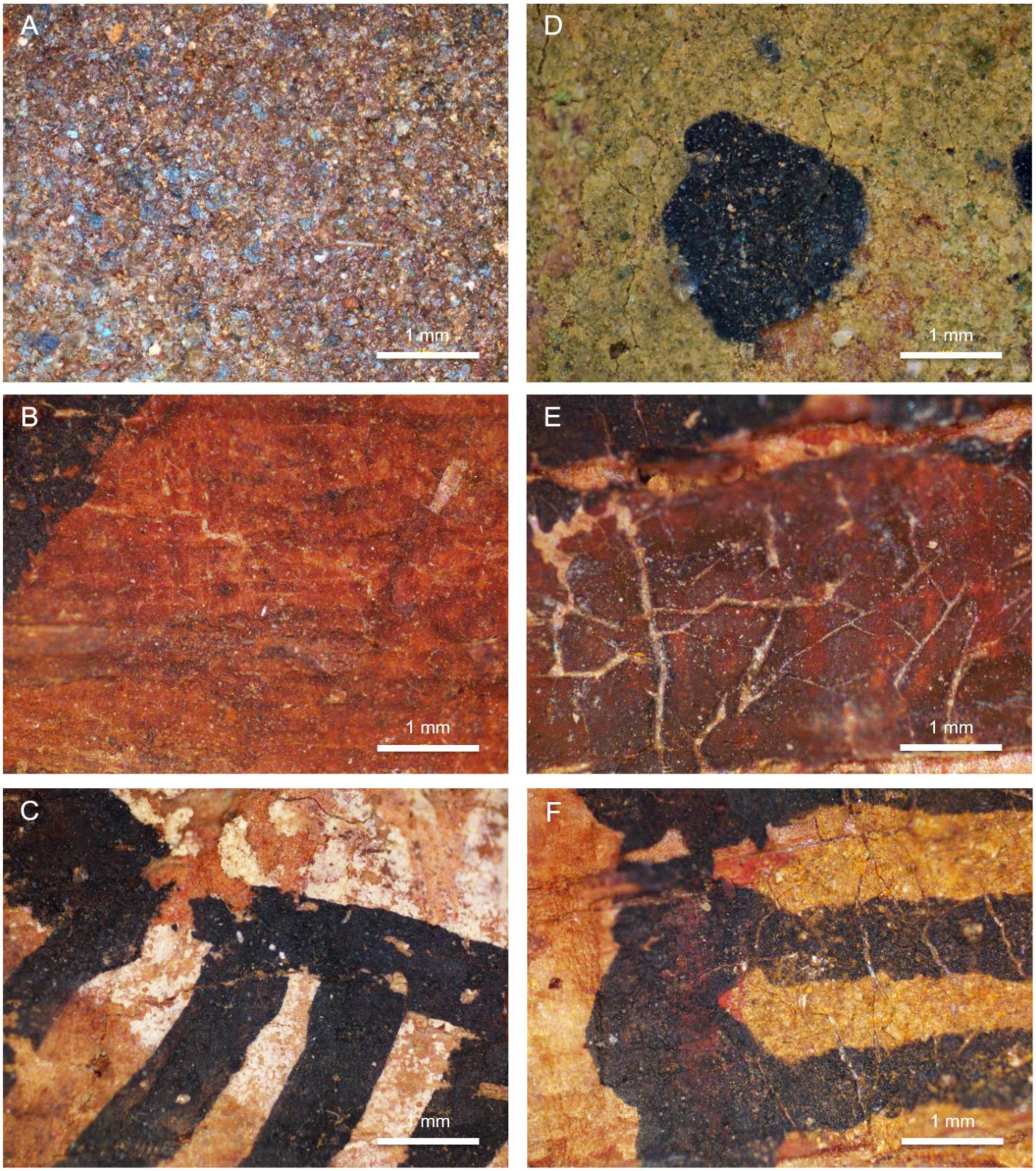


**Figure S1.** Optical microscopy images recorded on PAP-6 (A, B, C) and PAP-12 (D, E, F). A. Detail of the blue cap of the God, with large blue crystals. B. Detail of the red arm of the God. C. Detail of the white cloth of the deceased, and of the decorating black stripes. D. Detail of one of the black dots decorating the column, with a few blue crystals dispersed over it. E. Detail of the red solar disk of the cobra, showing some cracks. F. Detail of the column, where the red layer corresponding to the preparatory drawing can be seen under the black contour line.

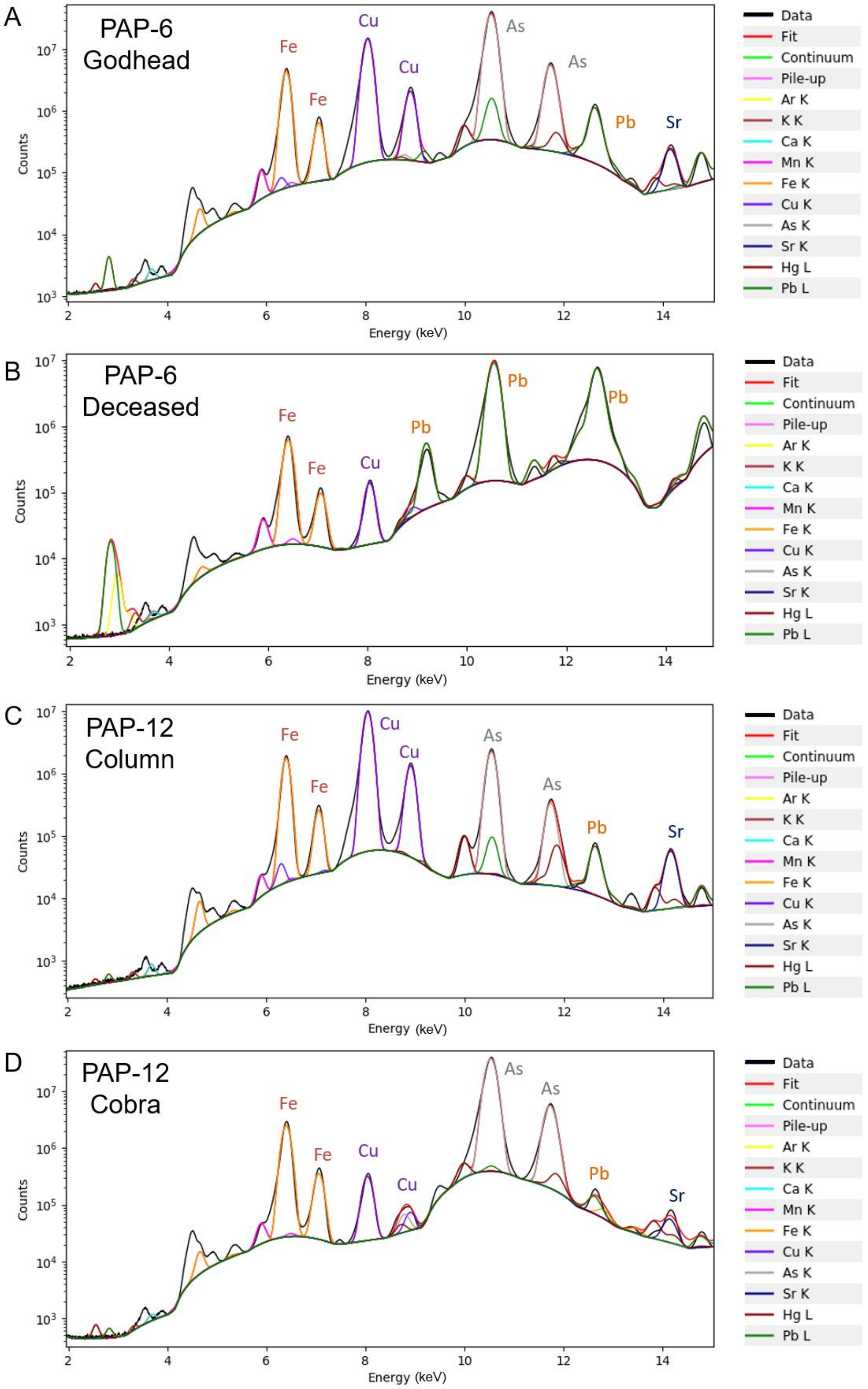


**Figure S2.** Sum spectra obtained from the X-ray fluorescence maps. A, Godhead (PAP-6); B, arm of the deceased (PAP-6); C, column (PAP-12); D, cobra head (PAP-12).

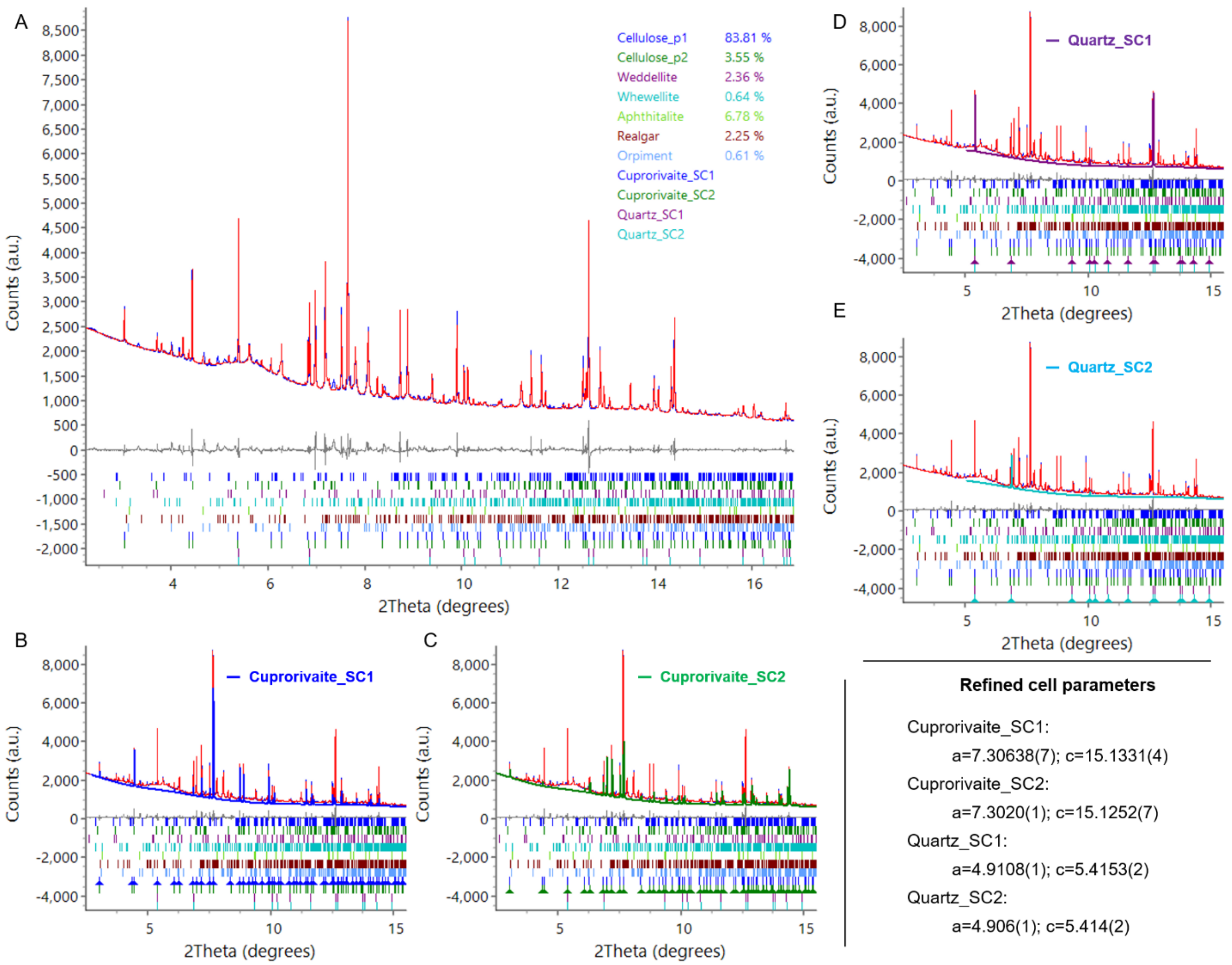


**Figure S3.** A. Combined Rietveld and Pawley refinements carried out against a diffraction pattern recorded on PAP-6 (X-10285, see Fig. S7) on the blue cap of the God ($R_{wp}$: 2.82%, $R_{exp}$: 0.86%, thin blue line: measured data, red line: calculated pattern, grey line: difference). Cellulose_p1 and Cellulose_p2 correspond to two different phases of cellulose. Cuprorivaite and quartz, present as large crystals, were modelled through Pawley fitting. When a large crystal is in the diffracting volume, single-crystal (SC) diffraction spots are recorded on 2D diffraction images, resulting in really sharp peaks after integration. A few large crystals will result in additional sharp peaks slightly shifted from each other for the same reflection. In the present case, this effect was taken into account by introducing two different phases of cuprorivaite (B and C, Cuprorivaite_SC1 and Cuprorivaite_SC2) and of quartz (D and E, Quartz_SC1 and Quartz_SC2), with slightly different refined values of their respective cell parameters, allowing quite different reflection intensities to be fitted.

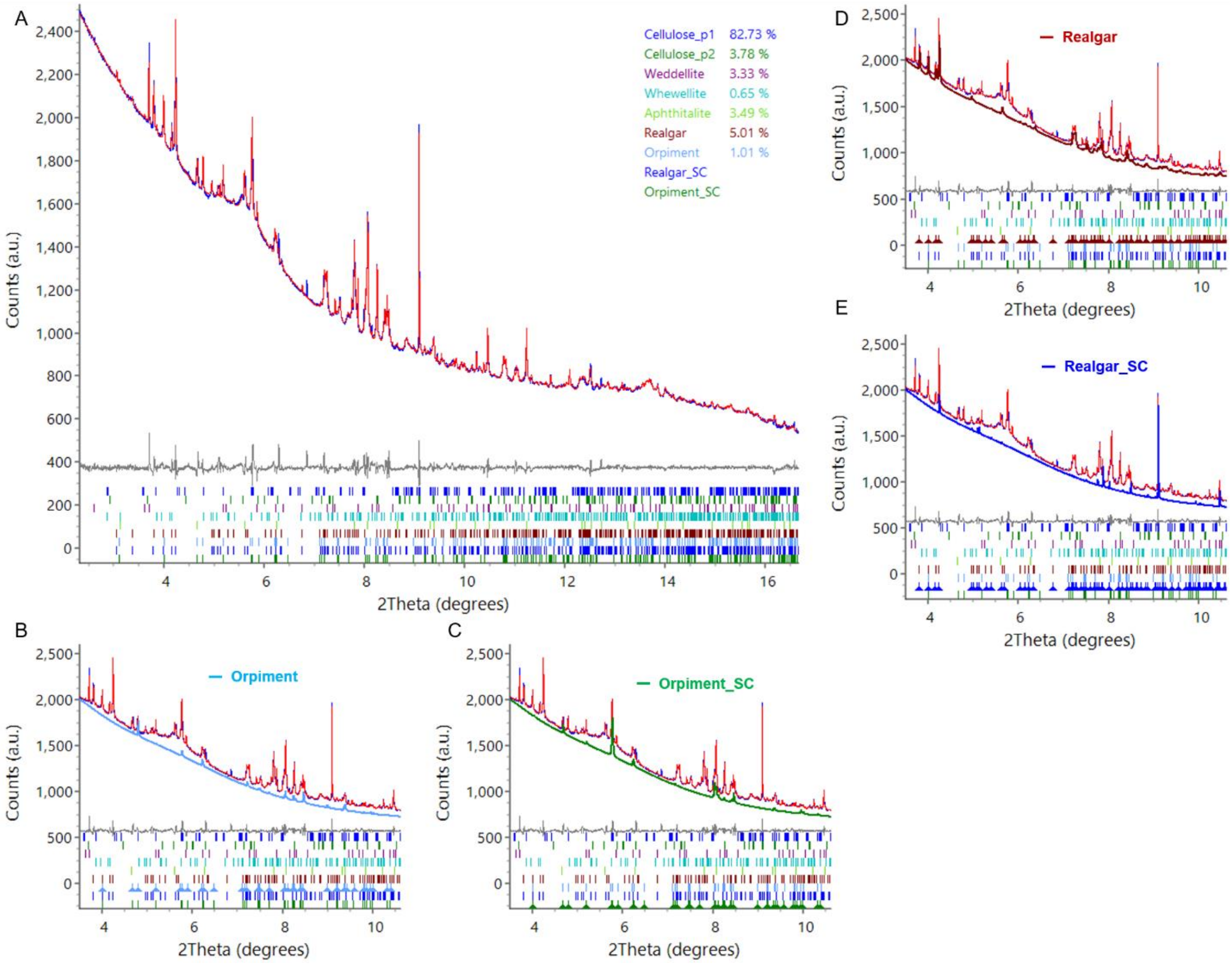


**Figure S4.** A. Combined Rietveld and Pawley refinements carried out against a diffraction pattern recorded on PAP-6 (X-11535, see Fig. S7) in the yellow region of the God face ($R_{wp}$: 1.05%, $R_{exp}$: 0.89%, thin blue line: measured data, red line: calculated pattern, grey line: difference). The orpiment and realgar phases found in the yellow regions are present as both large and smaller-size crystals. In the current example, for each of the orpiment and realgar phases, two phases were introduced, the first ones entering into the Rietveld refinement (Orpiment and Realgar in B and D, respectively), and the second ones modelled through Pawley fitting (Orpiment_SC and Realgar_SC in C and E, respectively, SC indicating a single-crystal contribution). The refinement was done in two steps. In the first step, scale factors of all the phases part of the Rietveld refinement were refined. Then, the scale factors of the orpiment and of the realgar phases were fixed, and the second phases modelled through Pawley fitting were introduced.

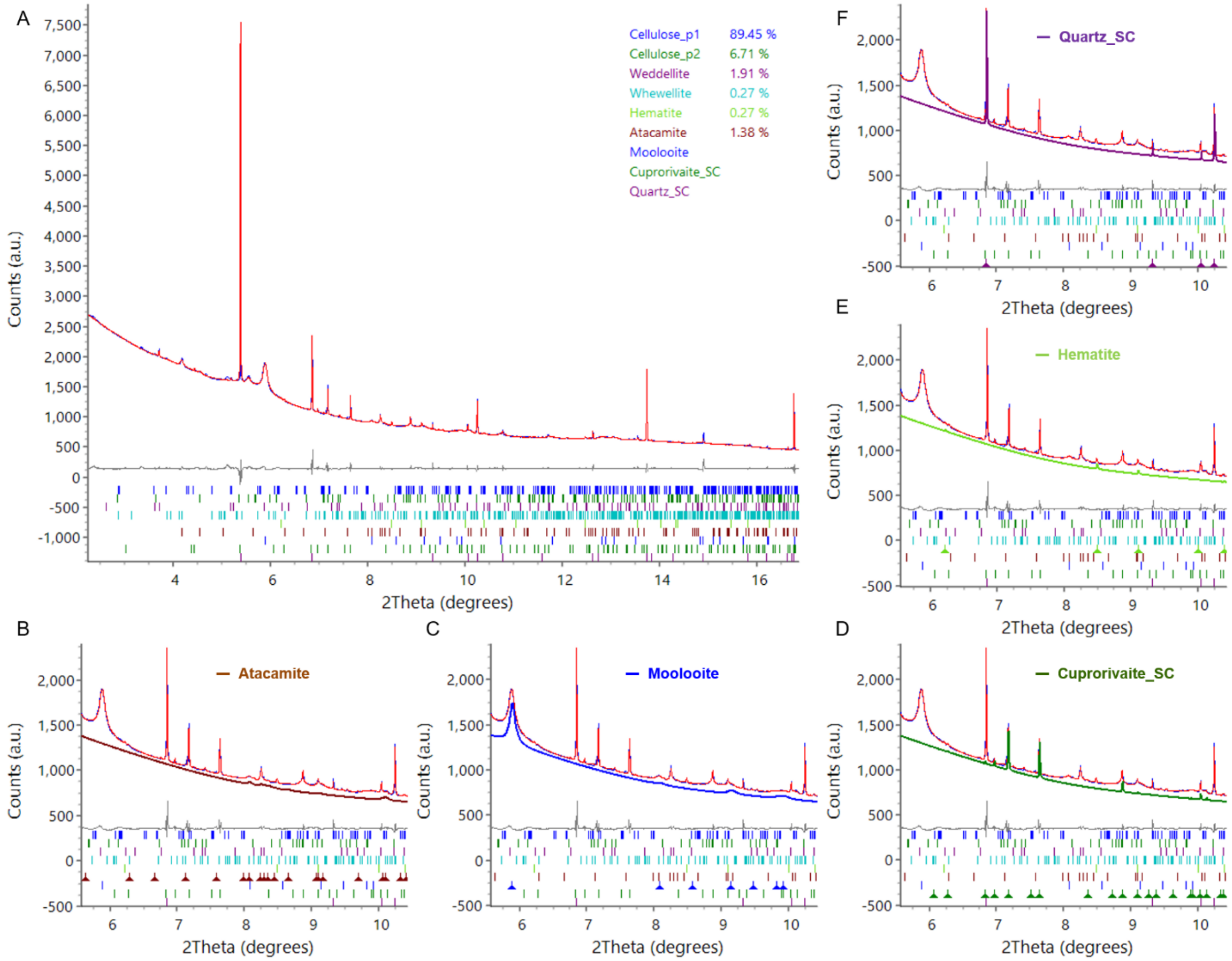


**Figure S5.** Combined Rietveld and Pawley refinements carried out against a diffraction pattern recorded on PAP-12 (X-17500, see Fig. S7) on one of the dark dots decorating the column ($R_{wp}$: 1.30%, $R_{exp}$: 0.55%, thin blue line: measured data, red line: calculated pattern, grey line: difference). In addition to the two phases of cellulose (p1 and p2), weddellite and whewellite, the phases of atacamite (B), moolooite (C), cuprorivaite (D), hematite (E), and quartz (F) are also present.

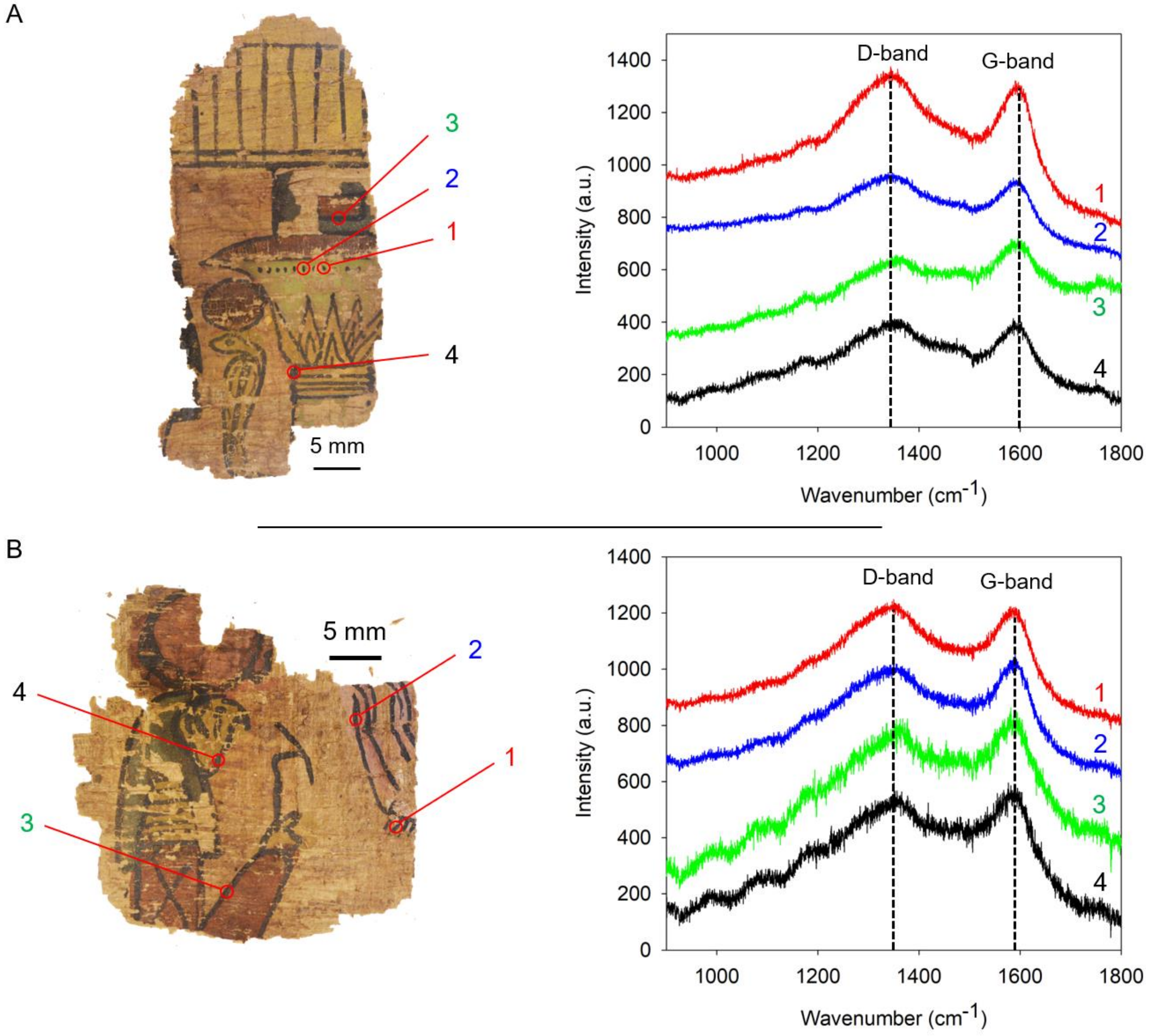


**Figure S6.** Raman spectroscopy spectra recorded in four different black points on PAP-12 (A) and on PAP-6 (B). The D-band and G-band characteristic of amorphous carbon were identified.

**Refinement results - Introduction**

The diffraction patterns collected over PAP-6 and PAP-12 were processed through combined Rietveld and Pawley refinements. The number of patterns refined and their positions on the four papyrus fragments are given in Fig. S7. Phases modelled through Pawley refinements are indicated with "_SC" in their name. Moolooite, for which we did not find information about the structural model, was also included through Pawley fitting. The two phases of cellulose are indicated as "Cellulose_p1" and "Cellulose_p2". A semi-amorphous phase, noted "UP" for "Unidentified Phase" and approximated to $K_2CO_3$ for the time being, was included in the refinements against the patterns collected on the arm of the deceased. Peak profiles of the different phases were modelled with a pseudo-Voight function. Cell parameters, peak profile parameters, scale factors (in the case of Rietveld) or reflection intensities (in the case of Pawley) were allowed to vary. All the refinements are shown in the next pages (blue: measured data, red: calculated pattern, grey: difference).

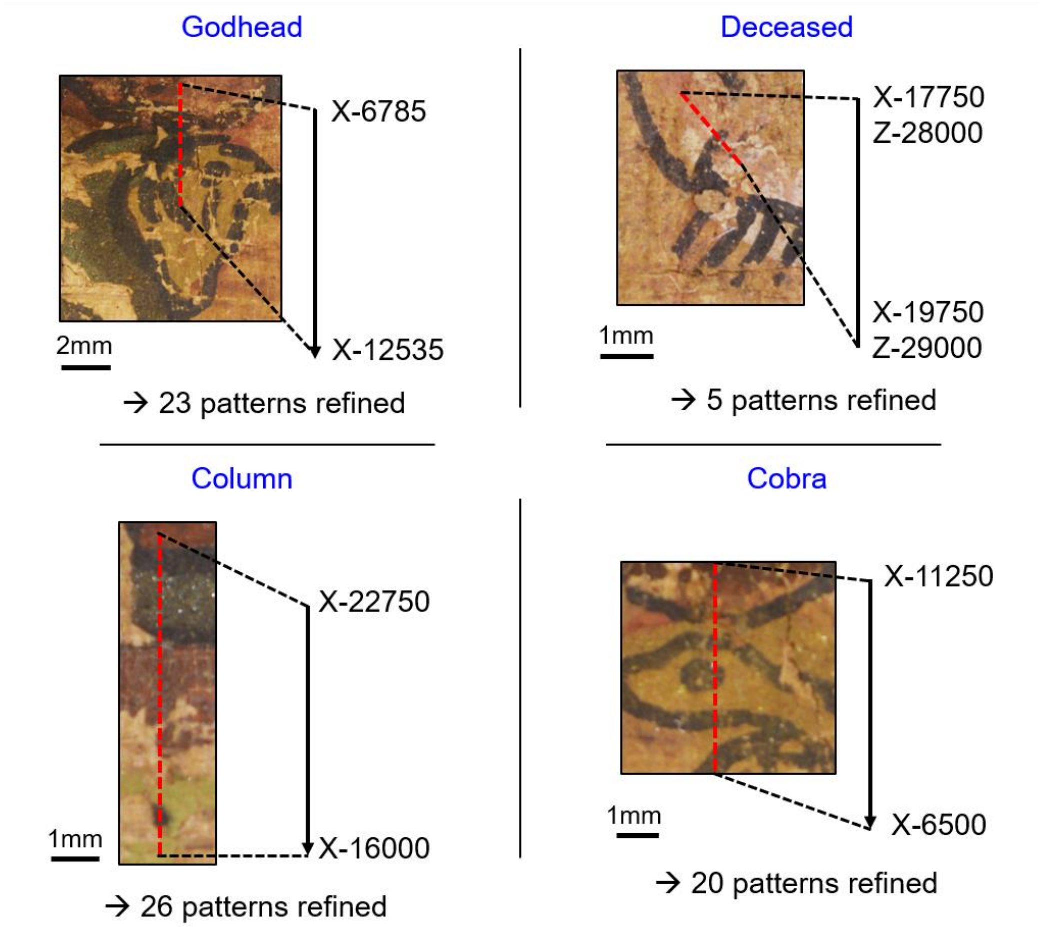


**Figure S7.** Powder diffraction patterns were collected along the four red lines over four different parts of PAP-6 (Godhead and arm of the deceased) and PAP-12 (column and cobra head). The labeled positions (in microns) as well as the number of patterns refined are indicated.

**Refinement results – Godhead**

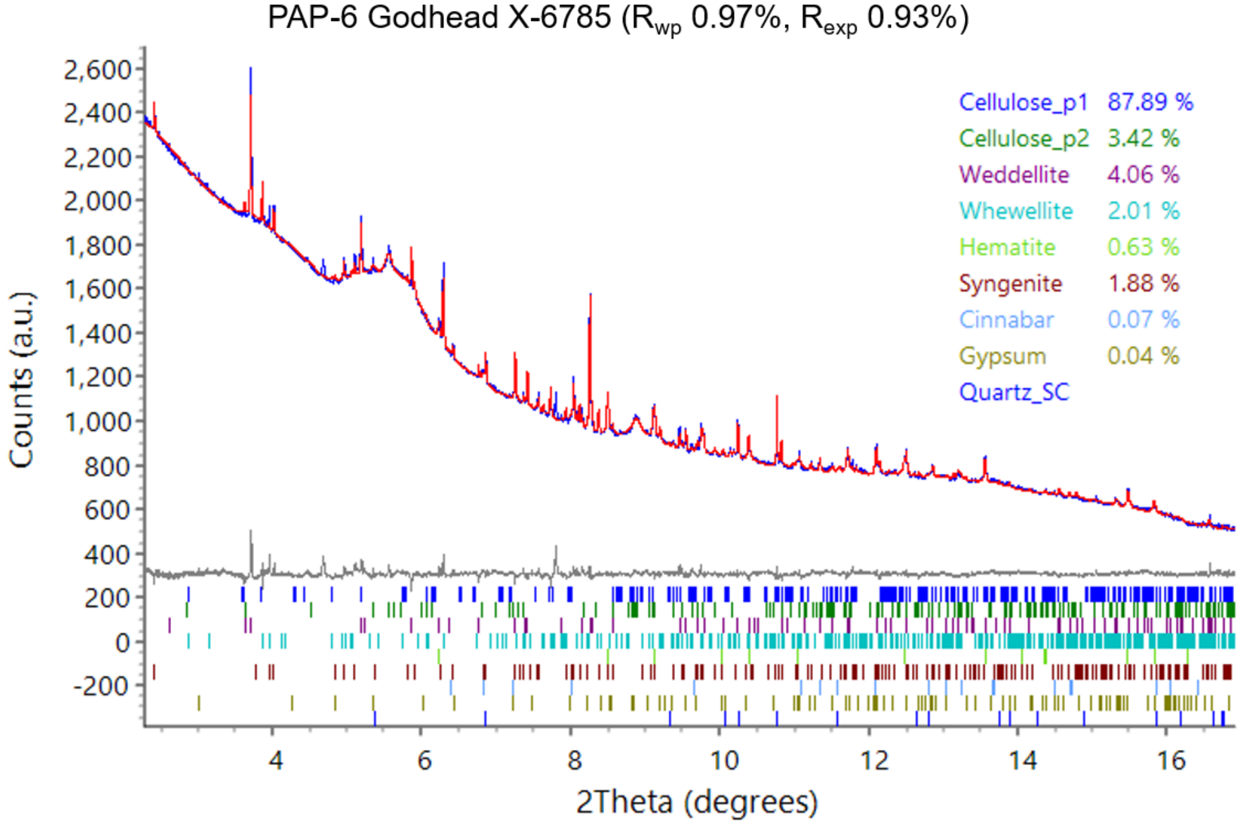


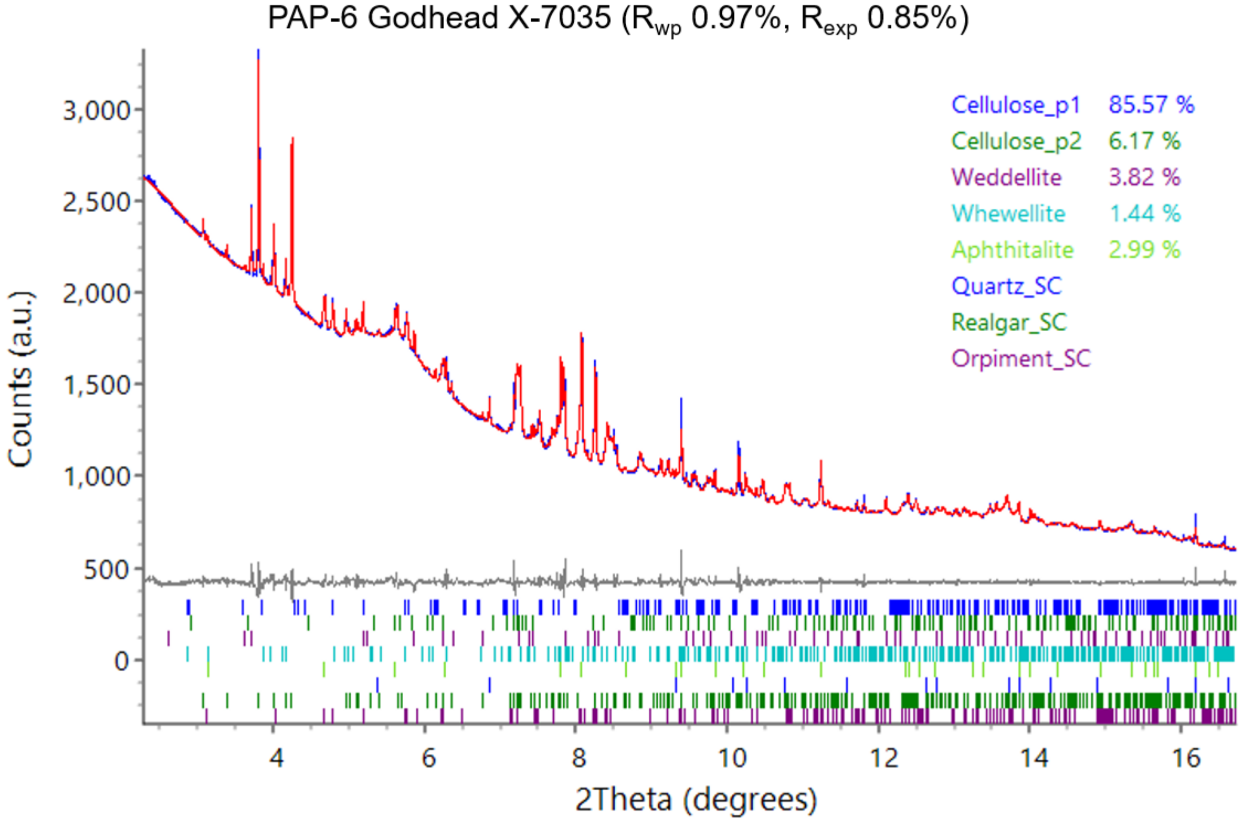

PAP-6 Godhead X-7285 ($R_{wp}$ 1.07%, $R_{exp}$ 0.99%)

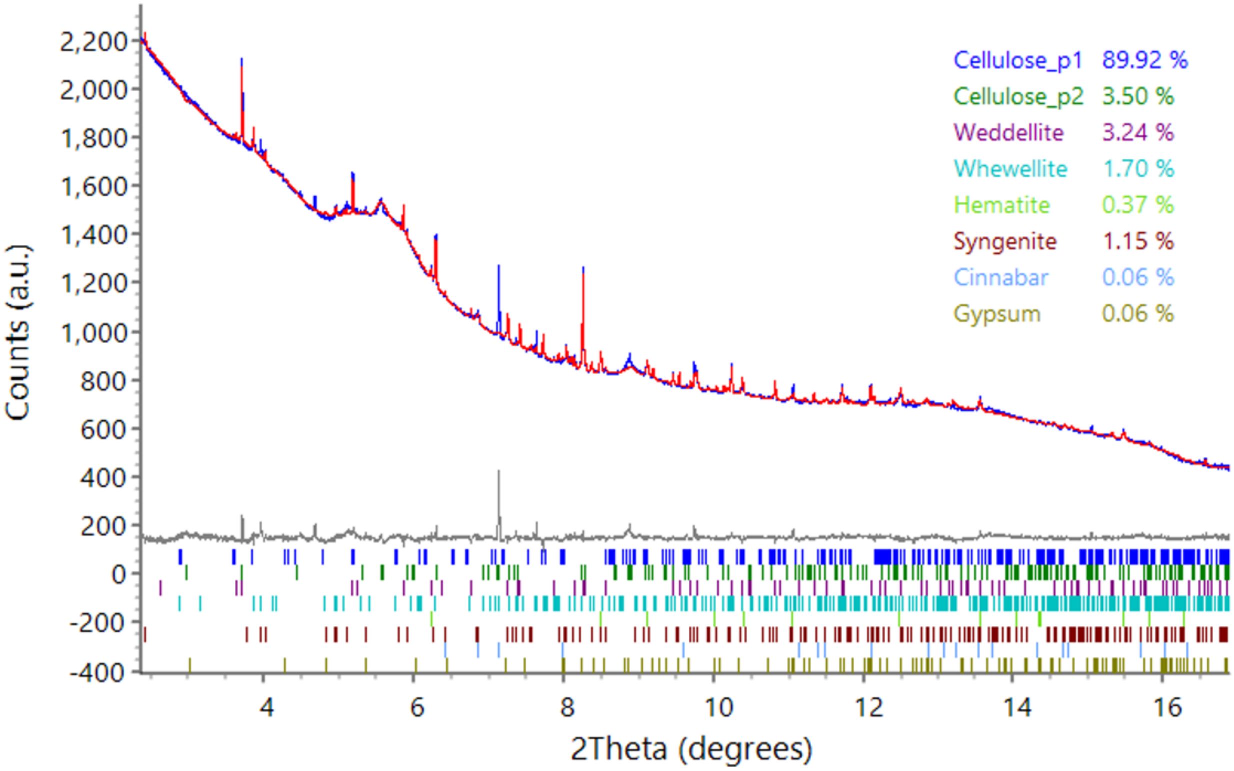


PAP-6 Godhead X-7535 ($R_{wp}$ 0.96%, $R_{exp}$ 0.93%)

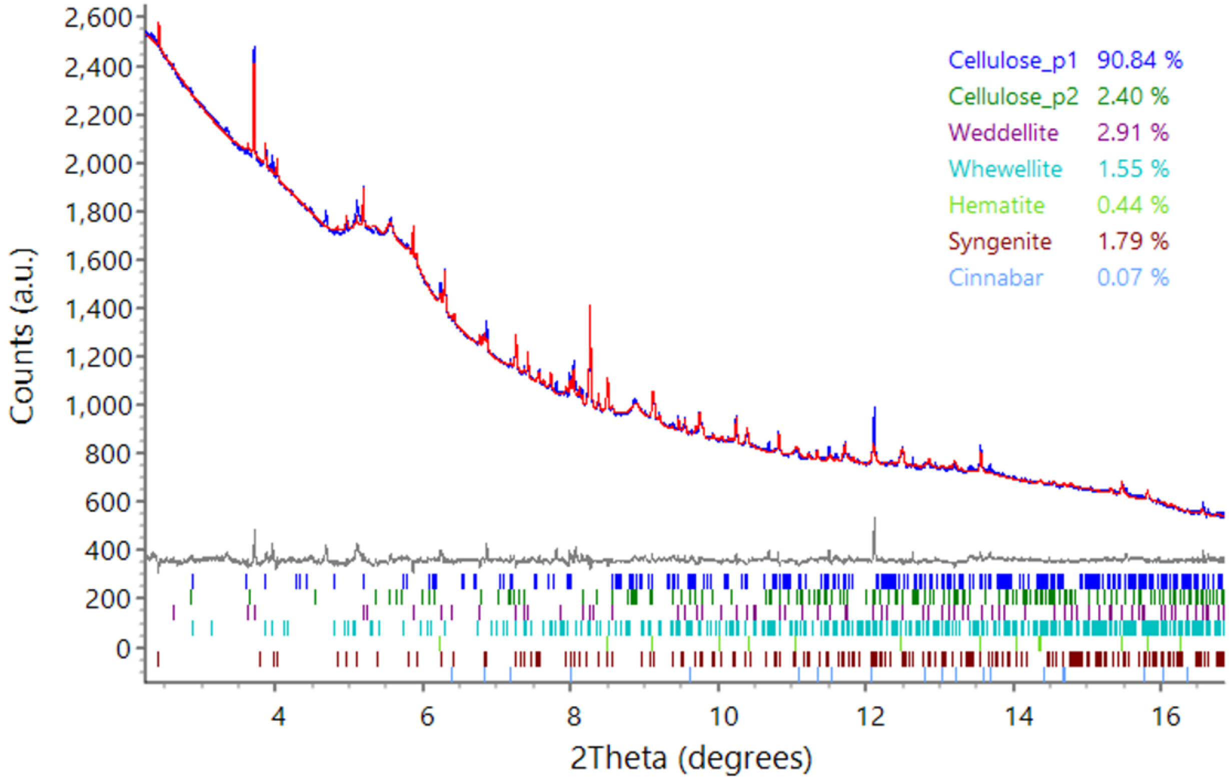

PAP-6 Godhead X-7785 ($R_{wp}$ 1.05%, $R_{exp}$ 0.99%)

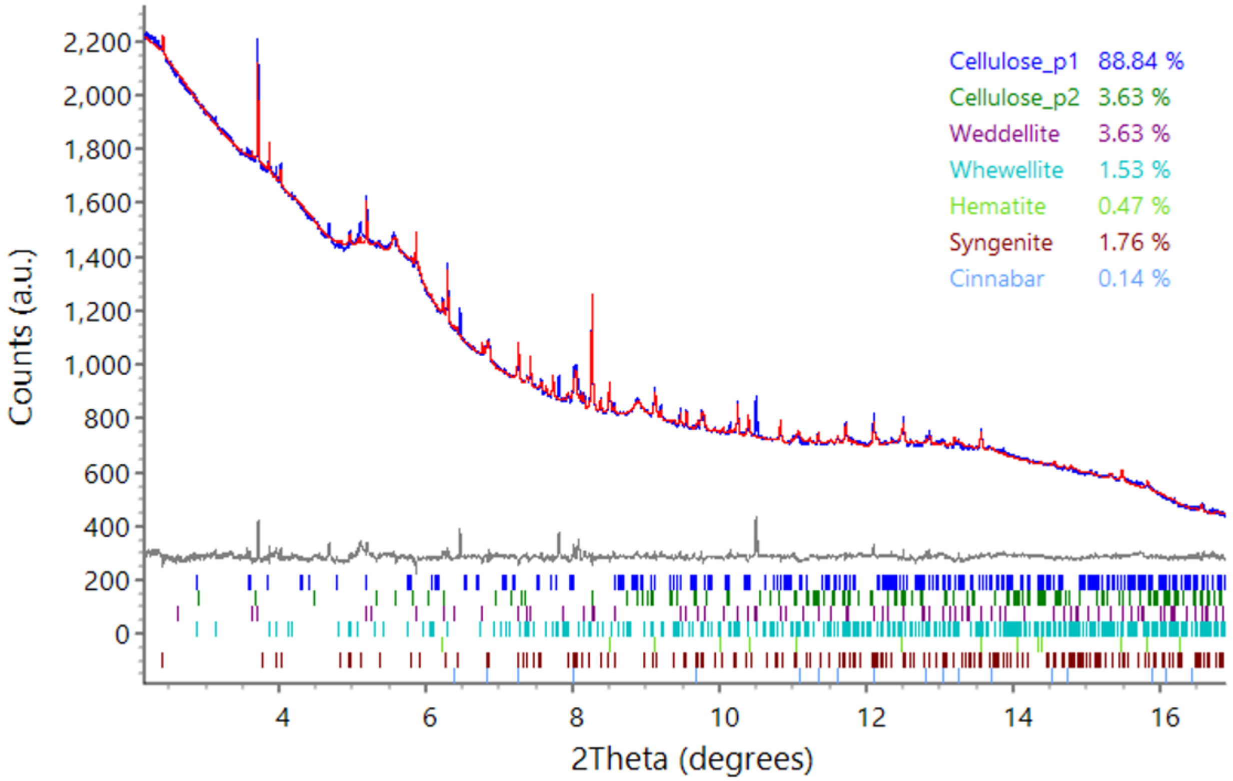


PAP-6 Godhead X-8035 ($R_{wp}$ 1.21%, $R_{exp}$ 0.93%)

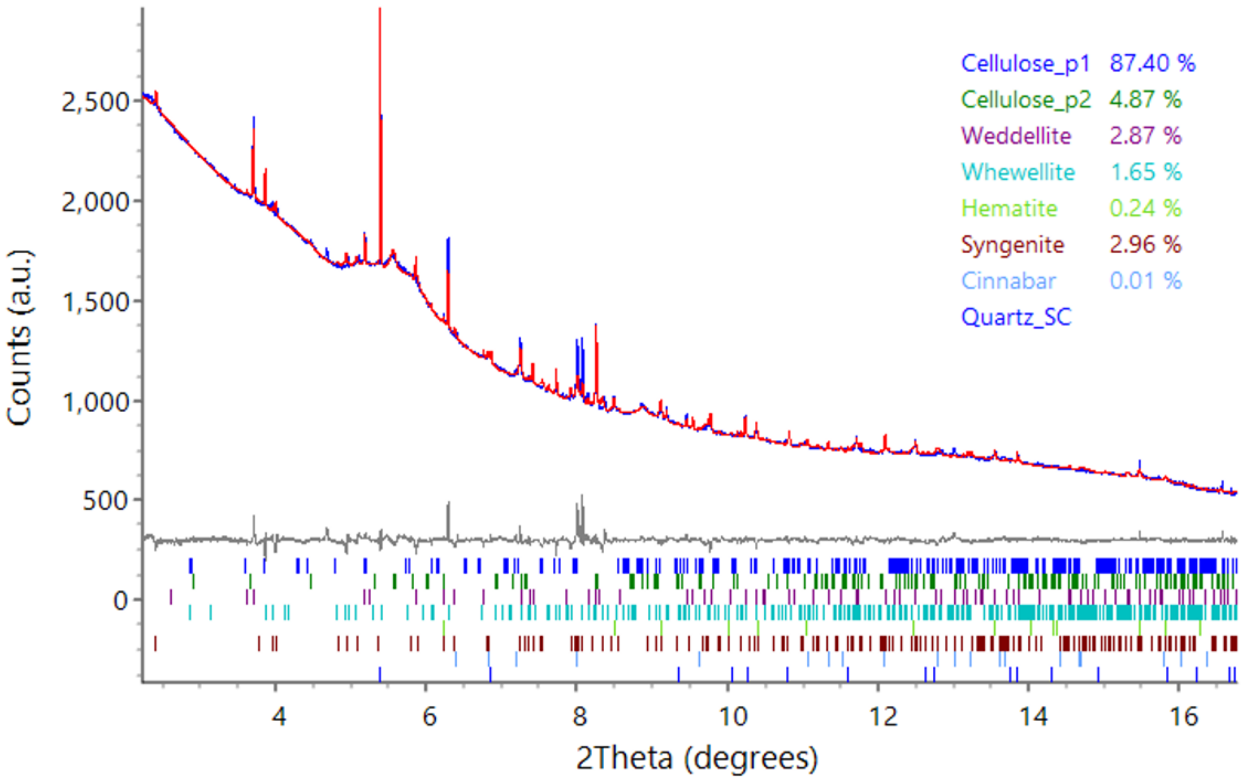

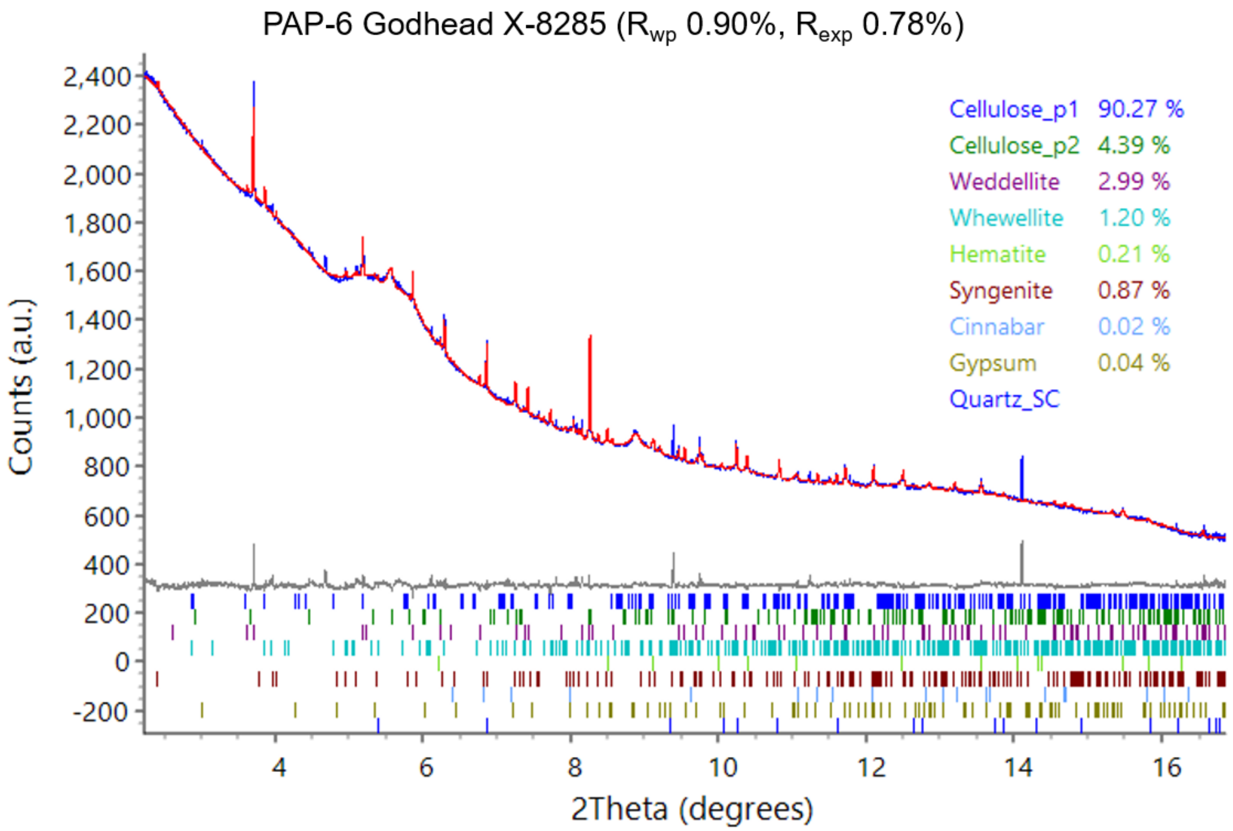

PAP-6 Godhead X-8285 ($R_{wp}$ 0.90%, $R_{exp}$ 0.78%)
Counts (a.u.)
2Theta (degrees)
Cellulose_p1 90.27 %
Cellulose_p2 4.39 %
Weddellite 2.99 %
Whewellite 1.20 %
Hematite 0.21 %
Syngenite 0.87 %
Cinnabar 0.02 %
Gypsum 0.04 %
Quartz_SC


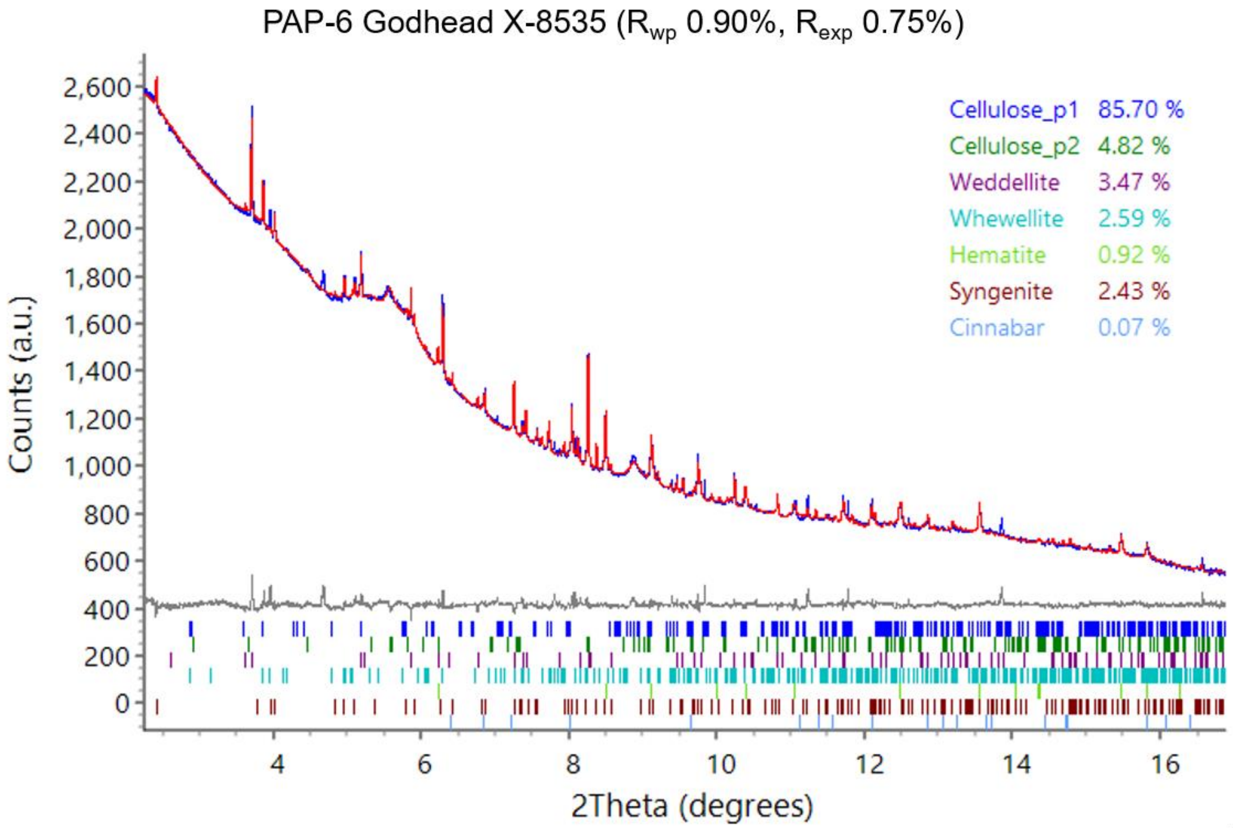

PAP-6 Godhead X-8535 ($R_{wp}$ 0.90%, $R_{exp}$ 0.75%)
Counts (a.u.)
2Theta (degrees)
Cellulose_p1 85.70 %
Cellulose_p2 4.82 %
Weddellite 3.47 %
Whewellite 2.59 %
Hematite 0.92 %
Syngenite 2.43 %
Cinnabar 0.07 %

PAP-6 Godhead X-8785 ($R_{wp}$ 1.00%, $R_{exp}$ 0.90%)

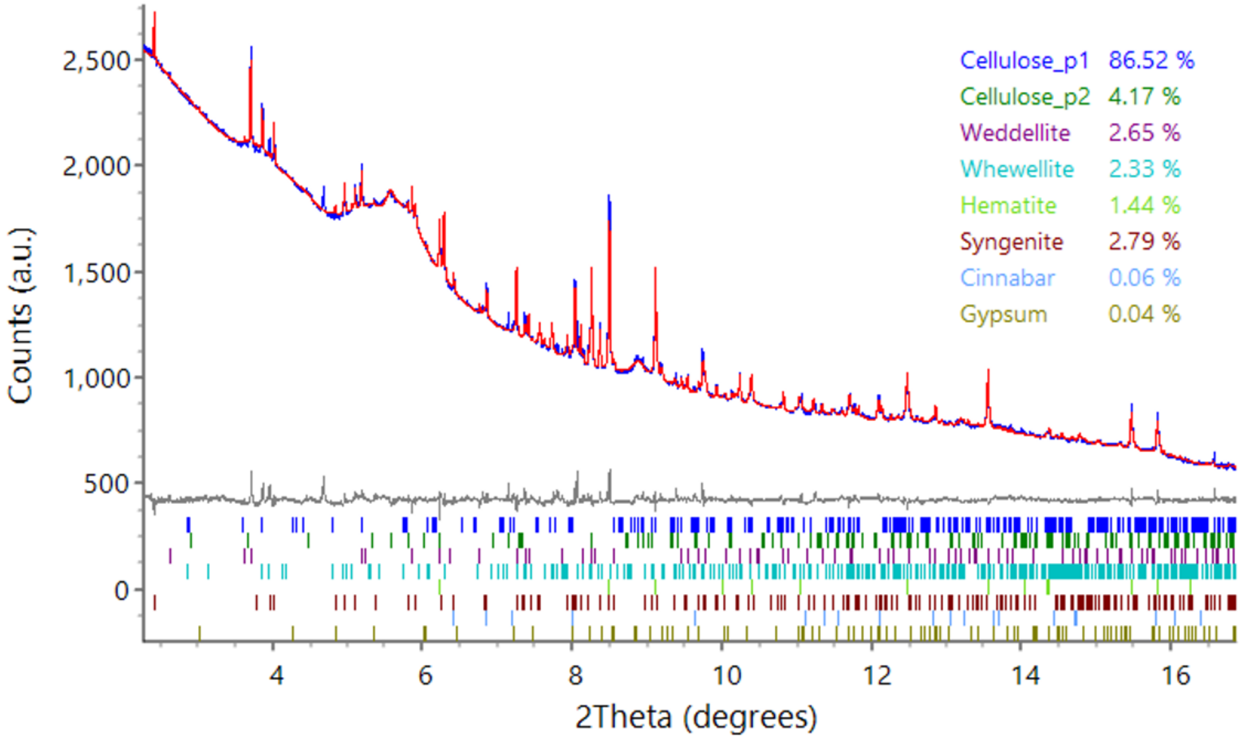


PAP-6 Godhead X-9035 ($R_{wp}$ 1.23%, $R_{exp}$ 0.89%)

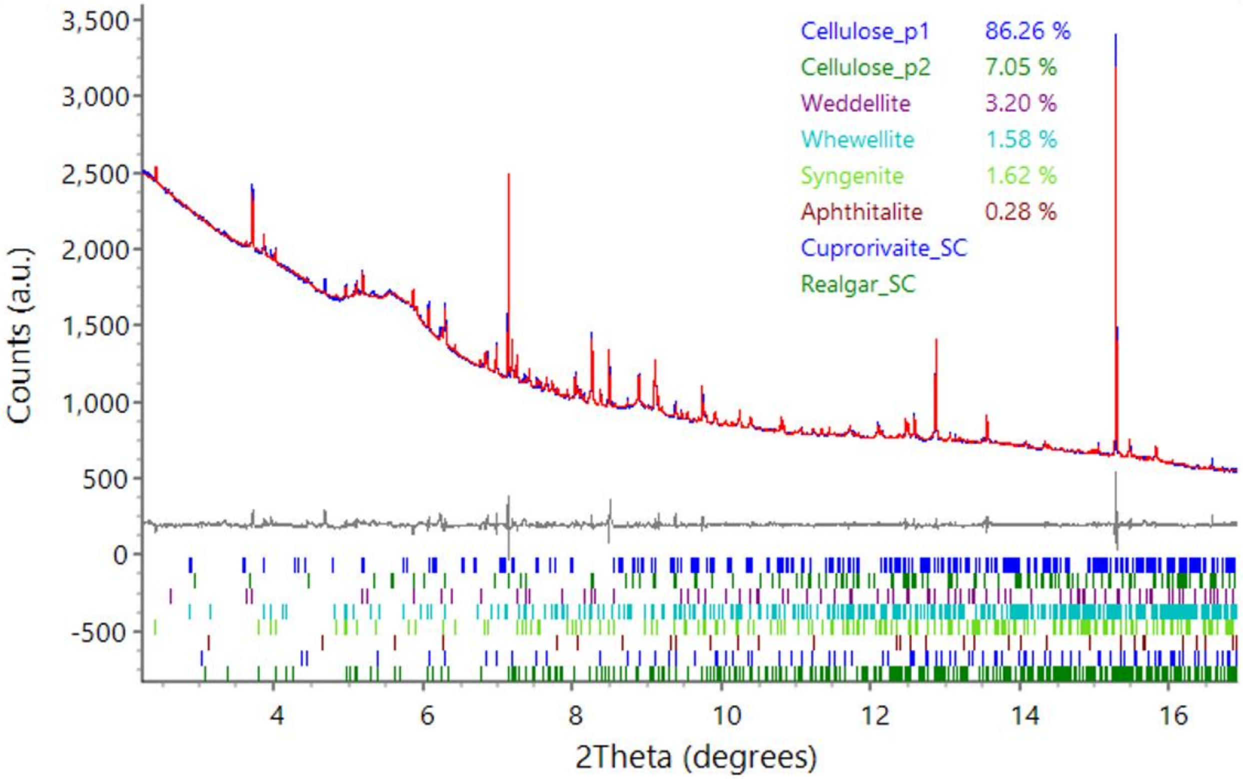

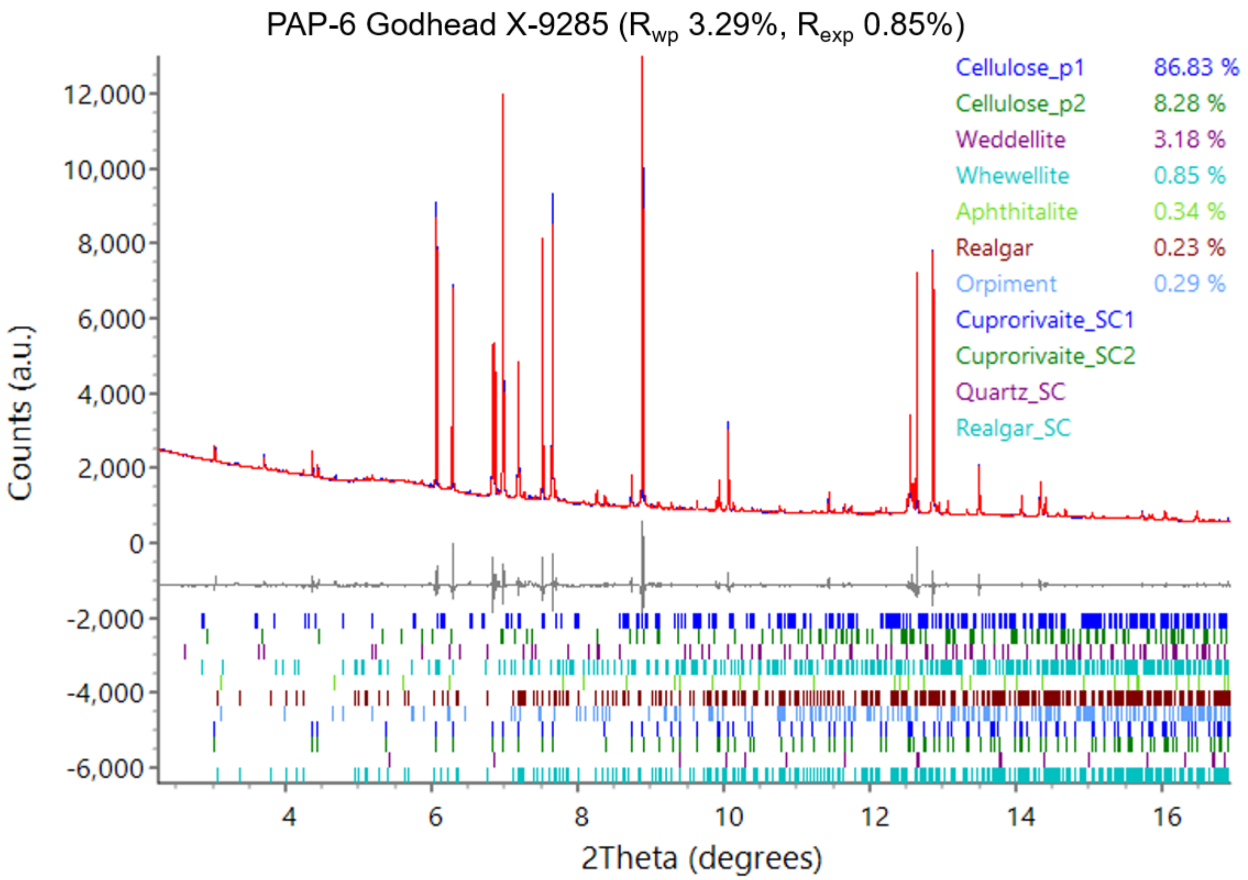

PAP-6 Godhead X-9285 ($R_{wp}$ 3.29%, $R_{exp}$ 0.85%)
Cellulose_p1 86.83 %
Cellulose_p2 8.28 %
Weddellite 3.18 %
Whewellite 0.85 %
Aphthitalite 0.34 %
Realgar 0.23 %
Orpiment 0.29 %
Cuprorivaite_SC1
Cuprorivaite_SC2
Quartz_SC
Realgar_SC
Counts (a.u.)
12,000
10,000
8,000
6,000
4,000
2,000
0
-2,000
-4,000
-6,000
4
6
8
10
12
14
16
2Theta (degrees)


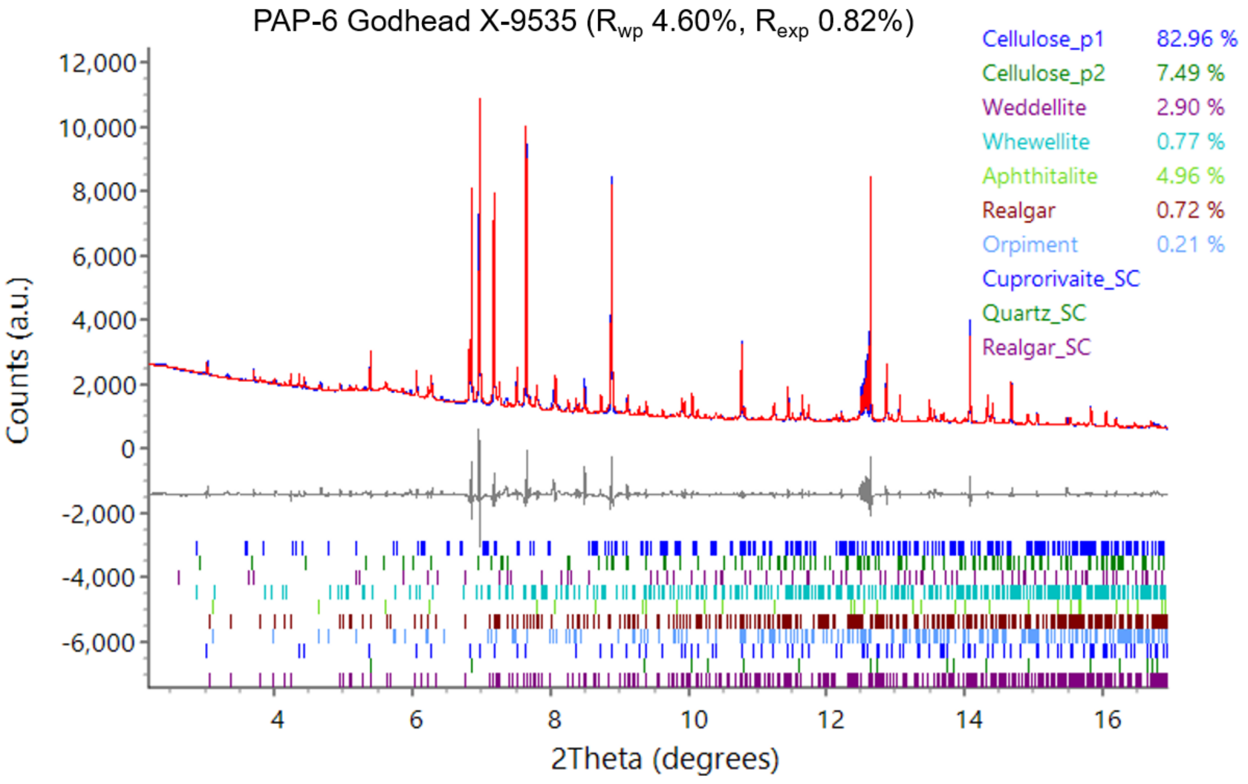

PAP-6 Godhead X-9535 ($R_{wp}$ 4.60%, $R_{exp}$ 0.82%)
Cellulose_p1 82.96 %
Cellulose_p2 7.49 %
Weddellite 2.90 %
Whewellite 0.77 %
Aphthitalite 4.96 %
Realgar 0.72 %
Orpiment 0.21 %
Cuprorivaite_SC
Quartz_SC
Realgar_SC
Counts (a.u.)
12,000
10,000
8,000
6,000
4,000
2,000
0
-2,000
-4,000
-6,000
4
6
8
10
12
14
16
2Theta (degrees)

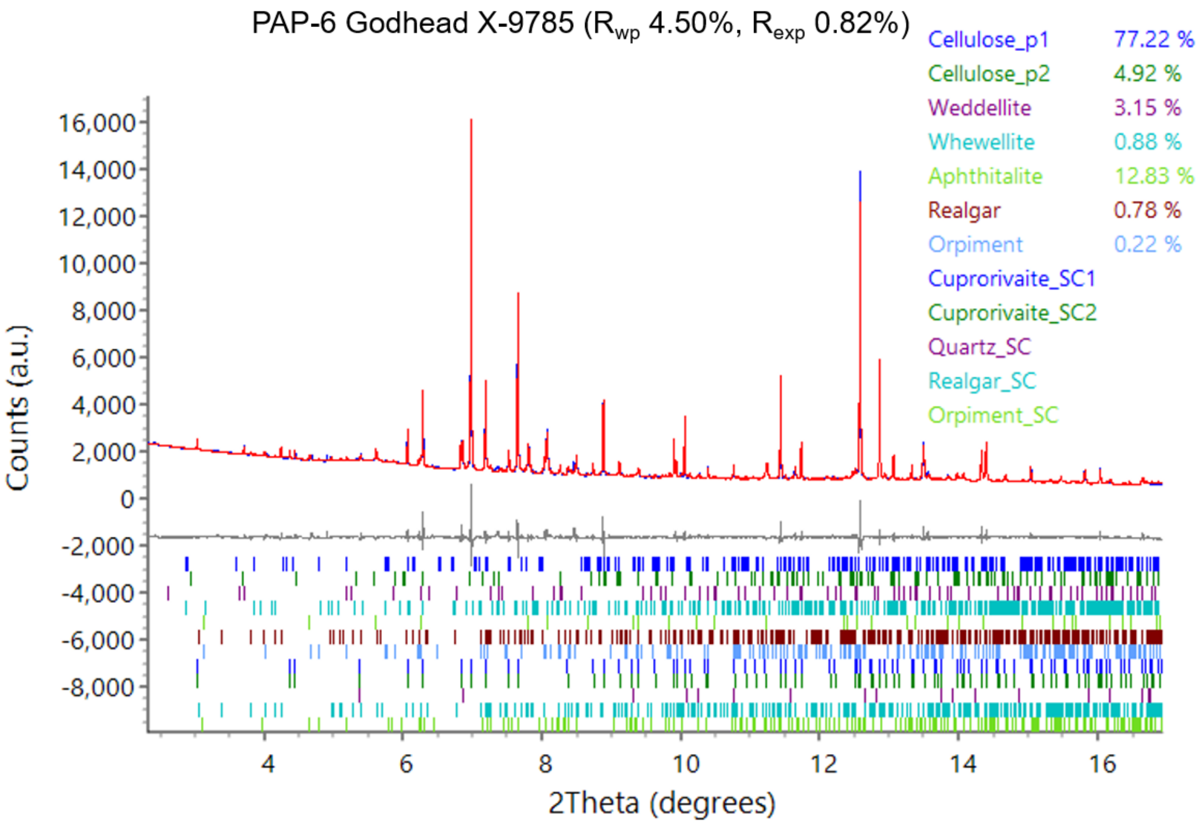

PAP-6 Godhead X-9785 ($R_{wp}$ 4.50%, $R_{exp}$ 0.82%)
Cellulose_p1 77.22 %
Cellulose_p2 4.92 %
Weddellite 3.15 %
Whewellite 0.88 %
Aphthitalite 12.83 %
Realgar 0.78 %
Orpiment 0.22 %
Cuprorivaite_SC1
Cuprorivaite_SC2
Quartz_SC
Realgar_SC
Orpiment_SC
Counts (a.u.)
2Theta (degrees)


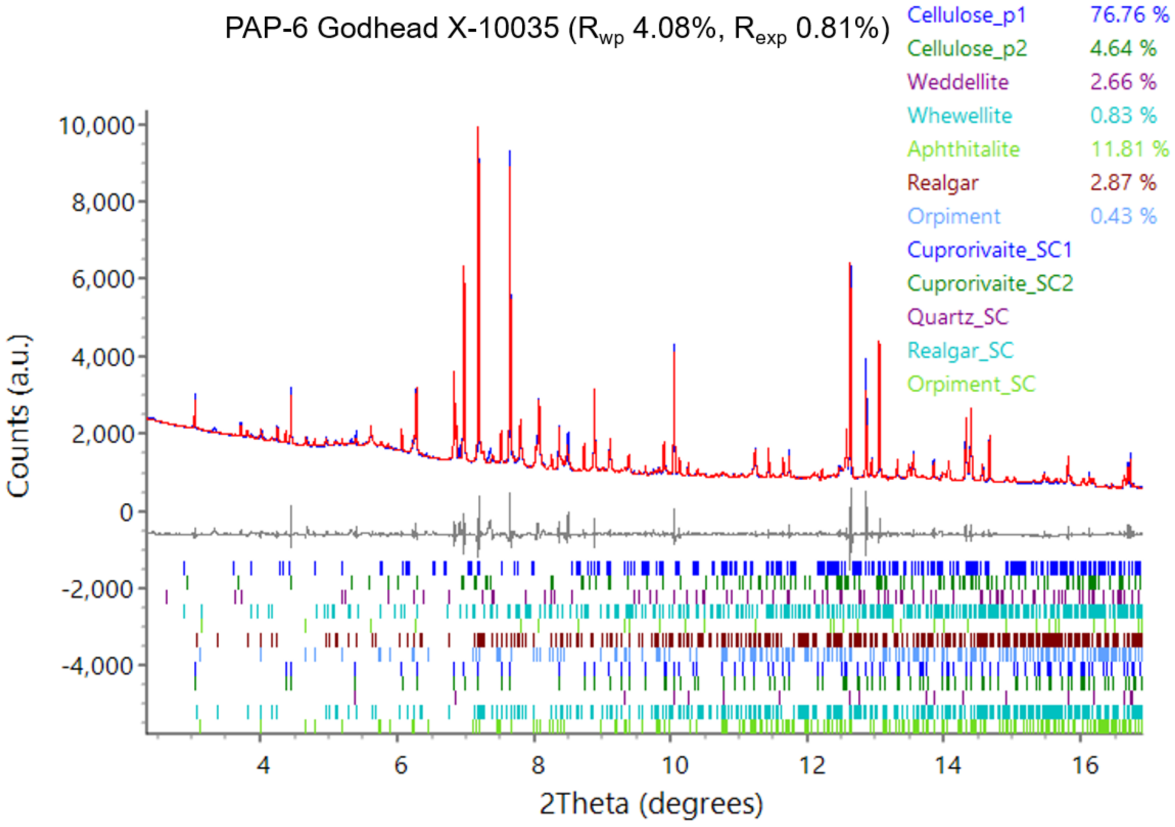

PAP-6 Godhead X-10035 ($R_{wp}$ 4.08%, $R_{exp}$ 0.81%)
Cellulose_p1 76.76 %
Cellulose_p2 4.64 %
Weddellite 2.66 %
Whewellite 0.83 %
Aphthitalite 11.81 %
Realgar 2.87 %
Orpiment 0.43 %
Cuprorivaite_SC1
Cuprorivaite_SC2
Quartz_SC
Realgar_SC
Orpiment_SC
Counts (a.u.)
2Theta (degrees)

PAP-6 Godhead X-10285 ($R_{wp}$ 2.82%, $R_{exp}$ 0.86%)

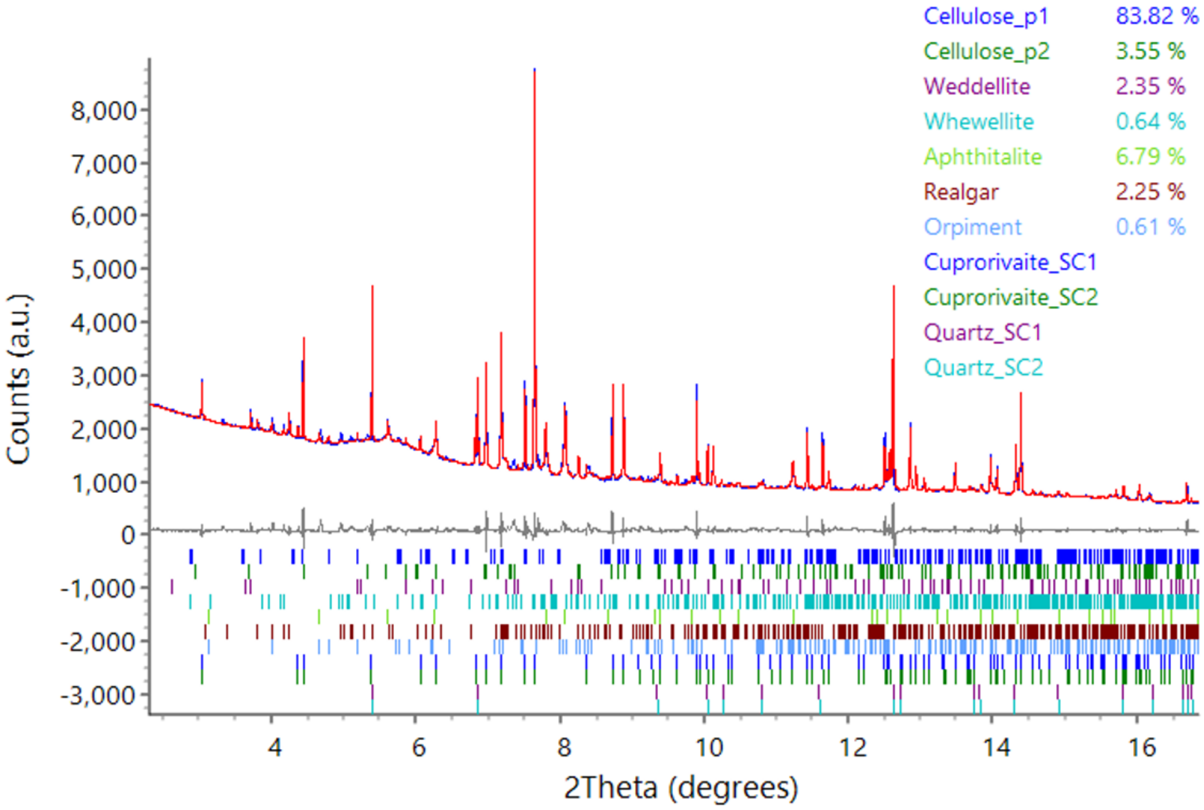


PAP-6 Godhead X-10535 ($R_{wp}$ 1.20%, $R_{exp}$ 0.86%)

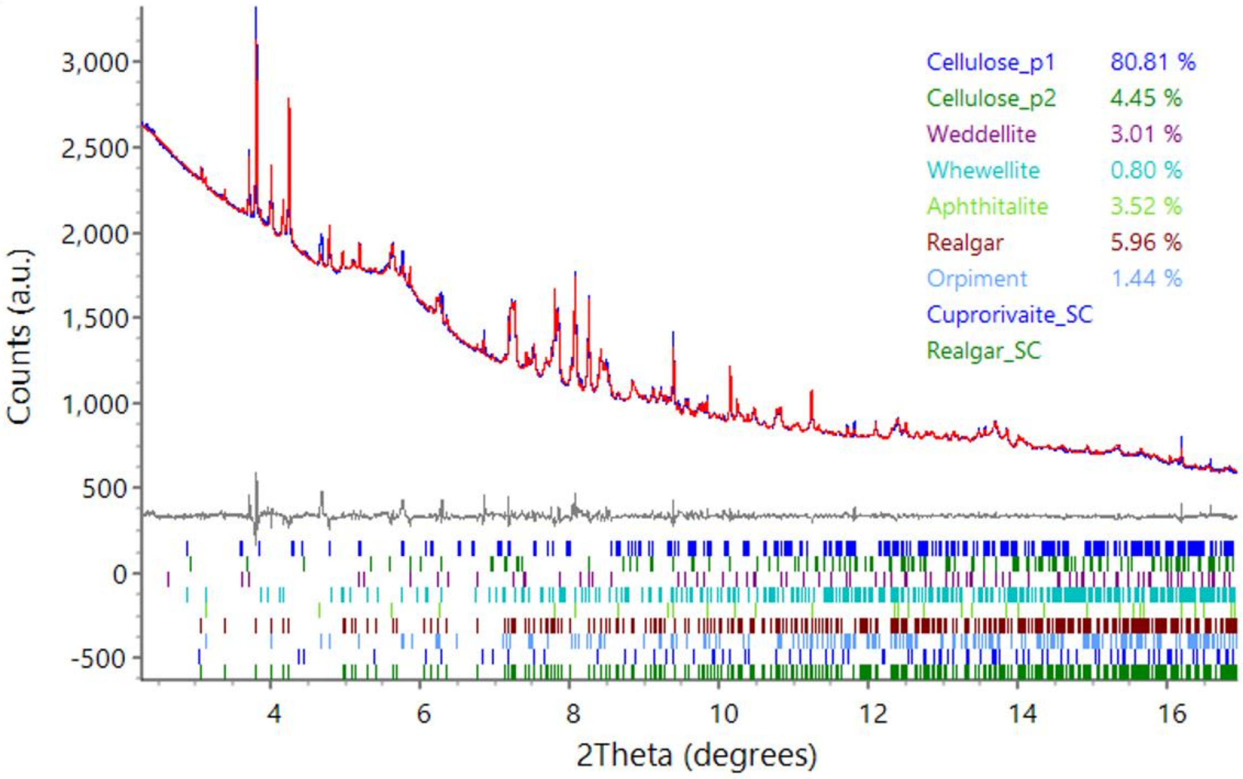

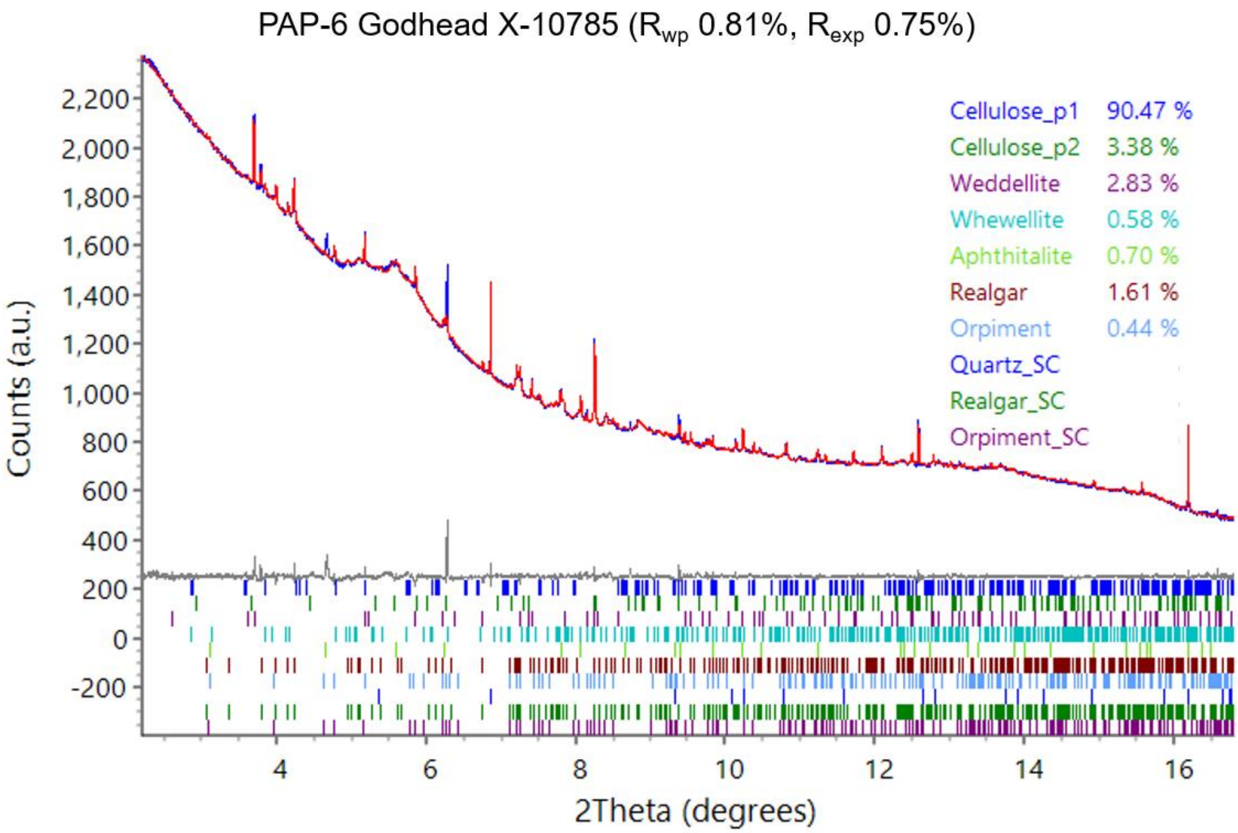
PAP-6 Godhead X-10785 ($R_{wp}$ 0.81%, $R_{exp}$ 0.75%)
Cellulose_p1 90.47 %
Cellulose_p2 3.38 %
Weddellite 2.83 %
Whewellite 0.58 %
Aphthitalite 0.70 %
Realgar 1.61 %
Orpiment 0.44 %
Quartz_SC
Realgar_SC
Orpiment_SC
Counts (a.u.)
2Theta (degrees)

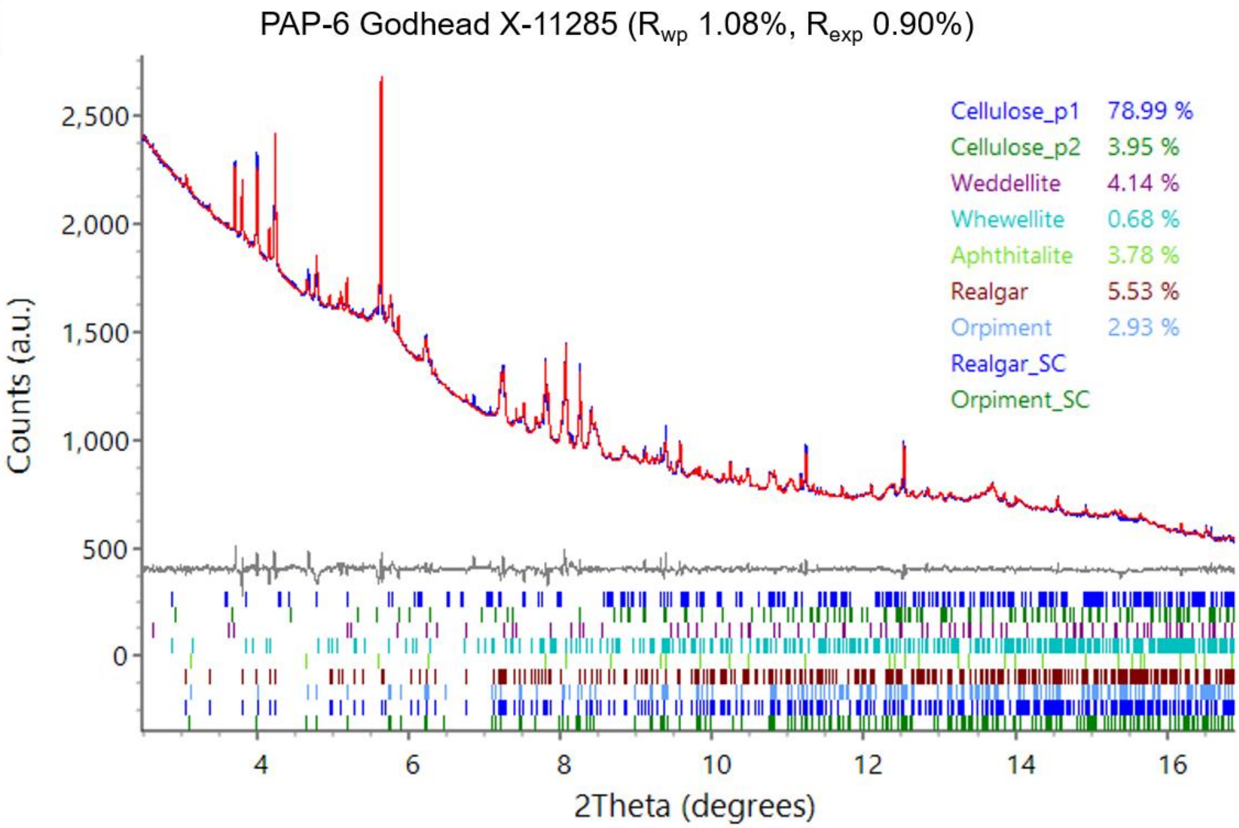
PAP-6 Godhead X-11285 ($R_{wp}$ 1.08%, $R_{exp}$ 0.90%)
Cellulose_p1 78.99 %
Cellulose_p2 3.95 %
Weddellite 4.14 %
Whewellite 0.68 %
Aphthitalite 3.78 %
Realgar 5.53 %
Orpiment 2.93 %
Realgar_SC
Orpiment_SC
Counts (a.u.)
2Theta (degrees)

PAP-6 Godhead X-11535 ($R_{wp}$ 1.05%, $R_{exp}$ 0.89%)

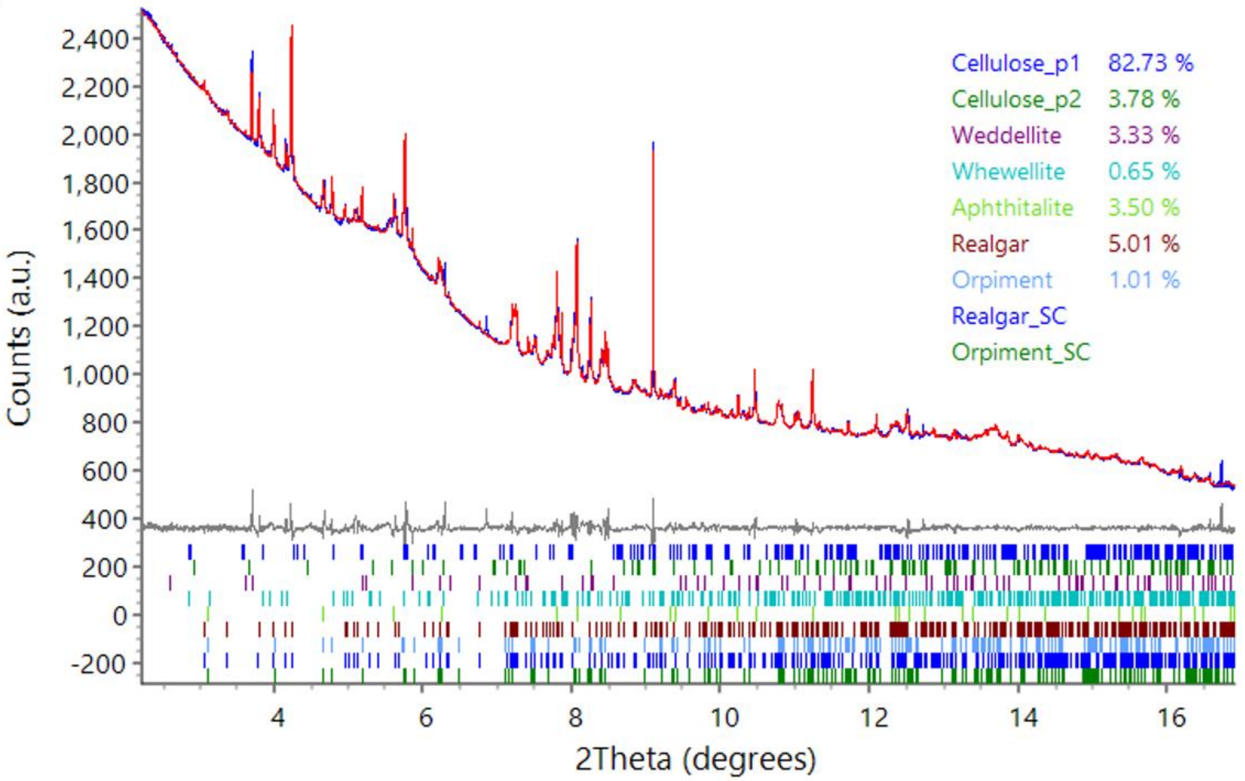


PAP-6 Godhead X-11785 ($R_{wp}$ 1.30%, $R_{exp}$ 0.85%)

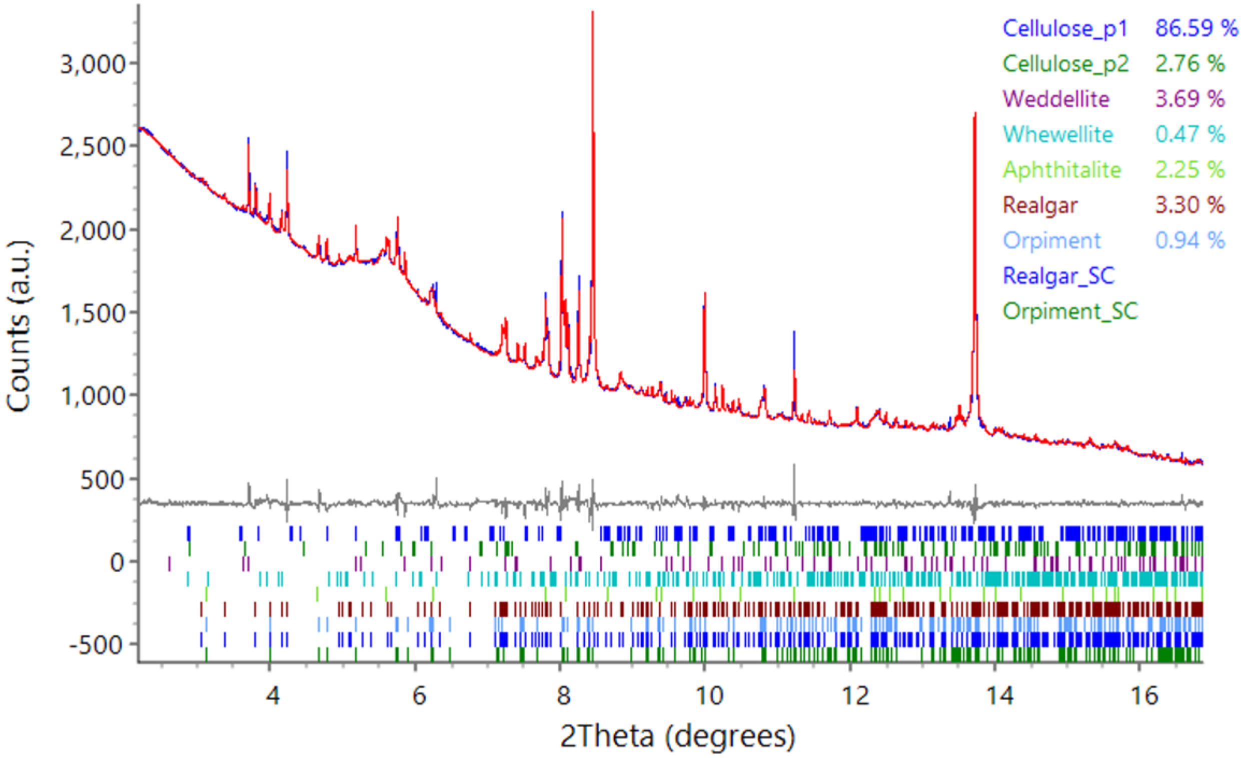

PAP-6 Godhead X-12035 ($R_{wp}$ 0.84%, $R_{exp}$ 0.79%)

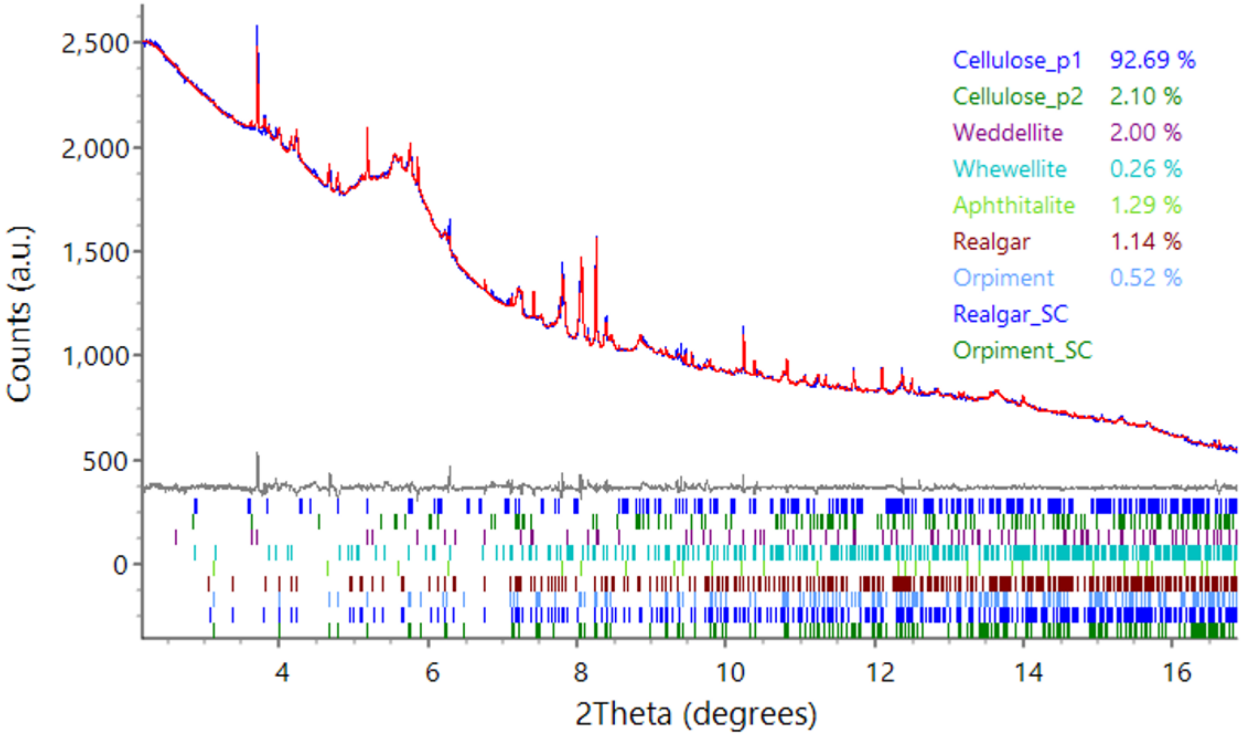


PAP-6 Godhead X-12285 ($R_{wp}$ 0.84%, $R_{exp}$ 0.82%)

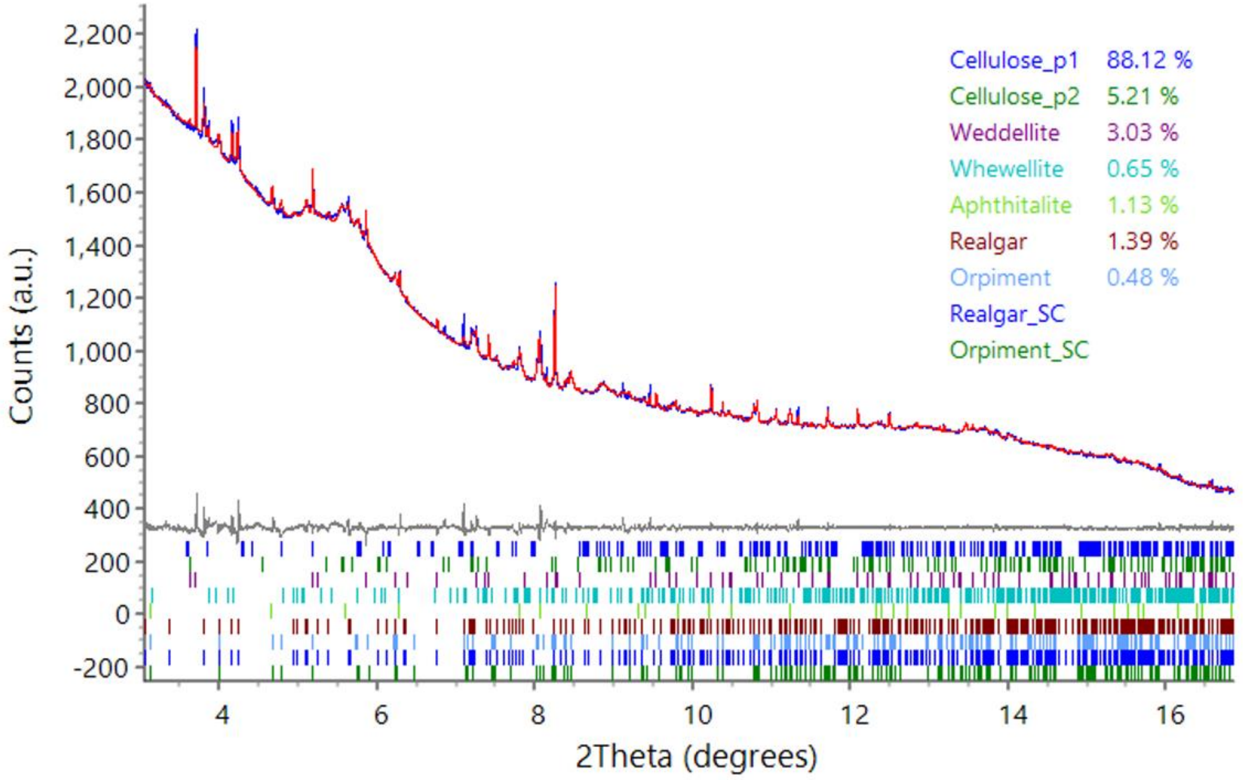

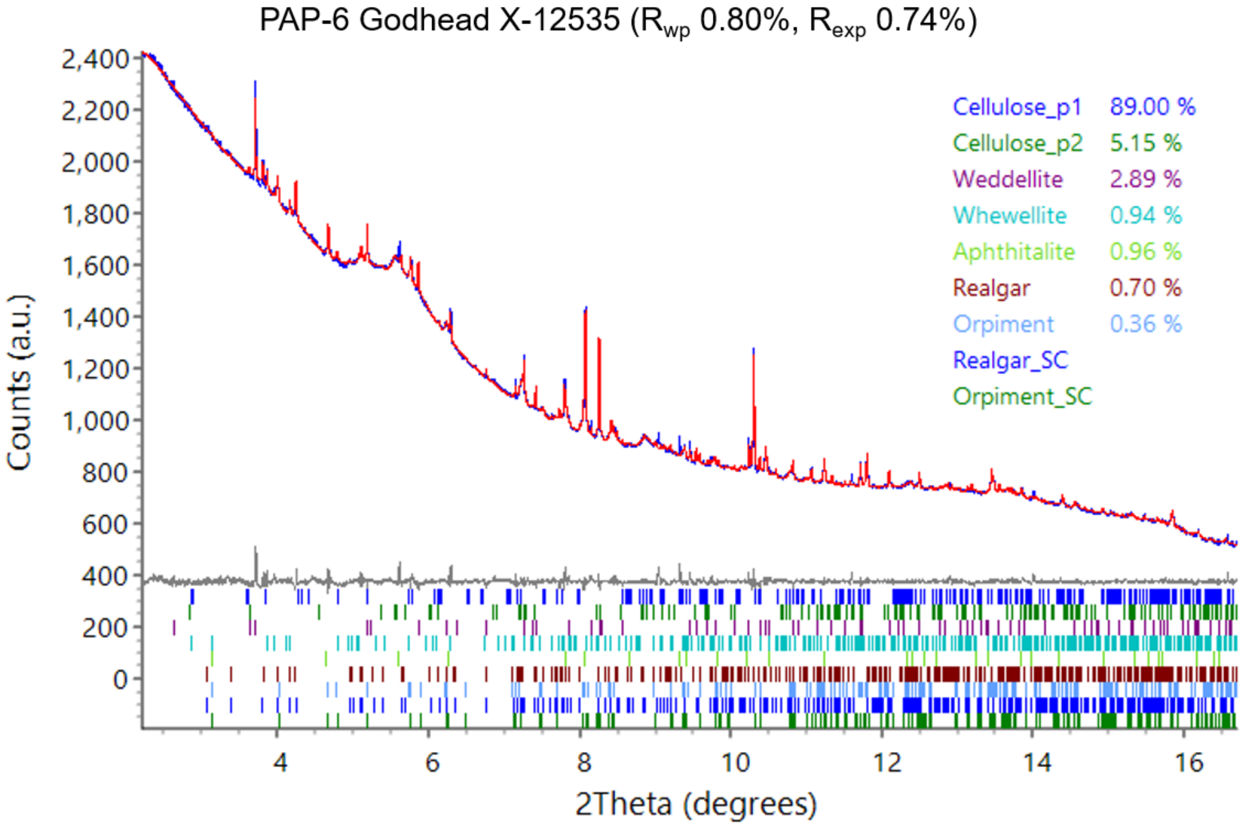

PAP-6 Godhead X-12535 (R$_{wp}$ 0.80%, R$_{exp}$ 0.74%)
Counts (a.u.)
2Theta (degrees)
Cellulose_p1 89.00 %
Cellulose_p2 5.15 %
Weddellite 2.89 %
Whewellite 0.94 %
Aphthitalite 0.96 %
Realgar 0.70 %
Orpiment 0.36 %
Realgar_SC
Orpiment_SC

**Refinement results – Deceased arm**

PAP-6 Deceased X-17750 Z-28000 ($R_{wp}$ 3.34%, $R_{exp}$ 2.61%)

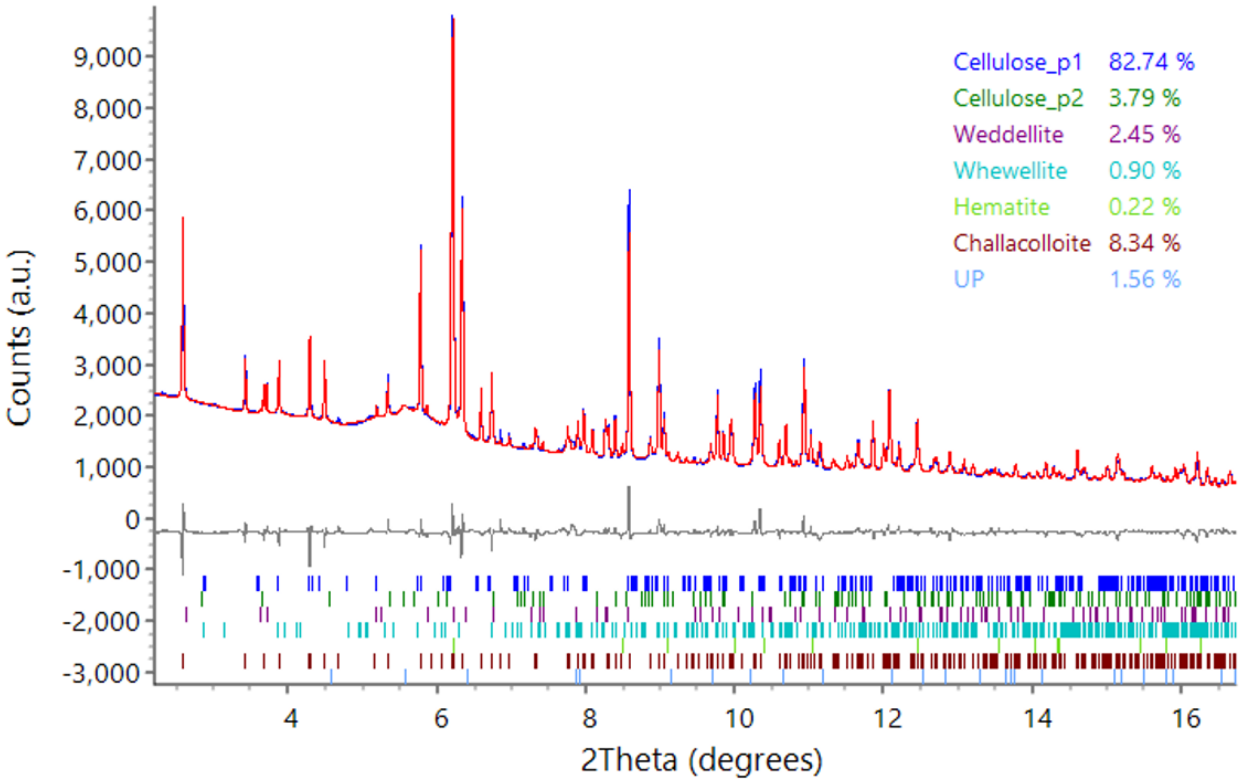


PAP-6 Deceased X-18250 Z-28250 ($R_{wp}$ 4.66%, $R_{exp}$ 2.52%)

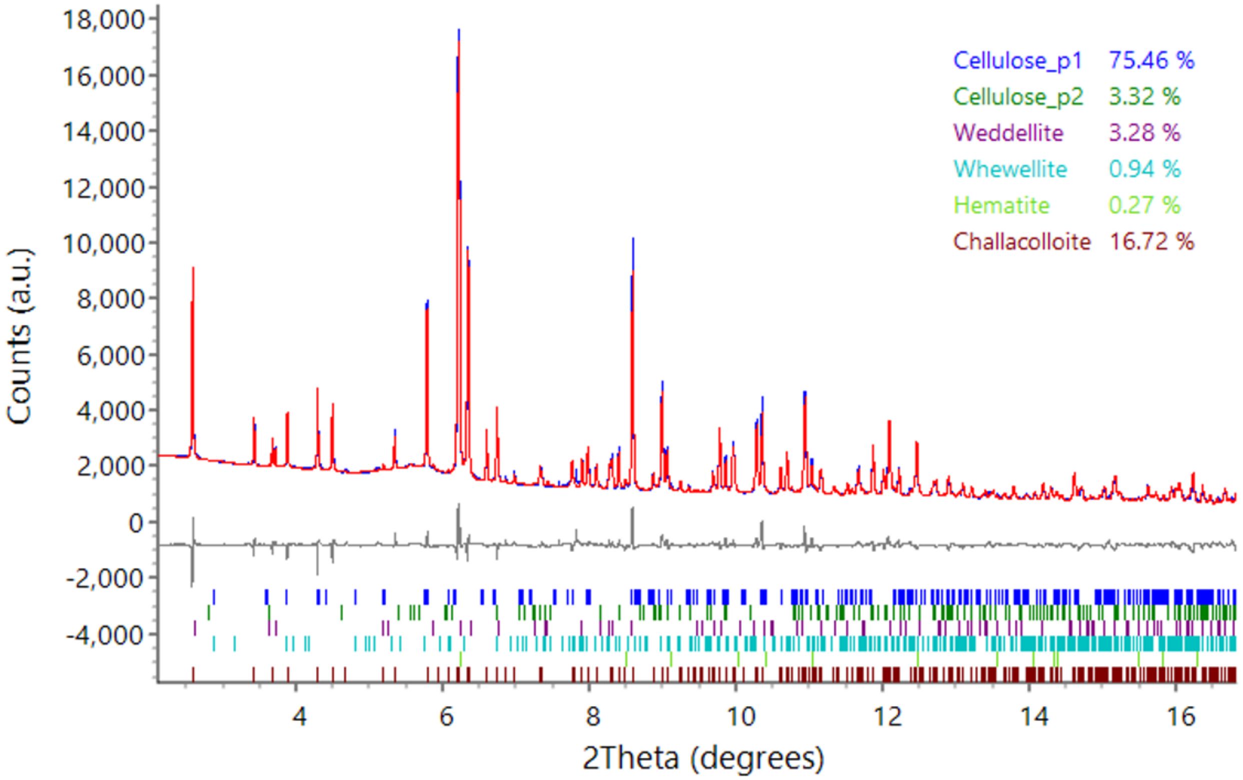

PAP-6 Deceased X-18750 Z-28500 ($R_{wp}$ 2.91%, $R_{exp}$ 2.60%)

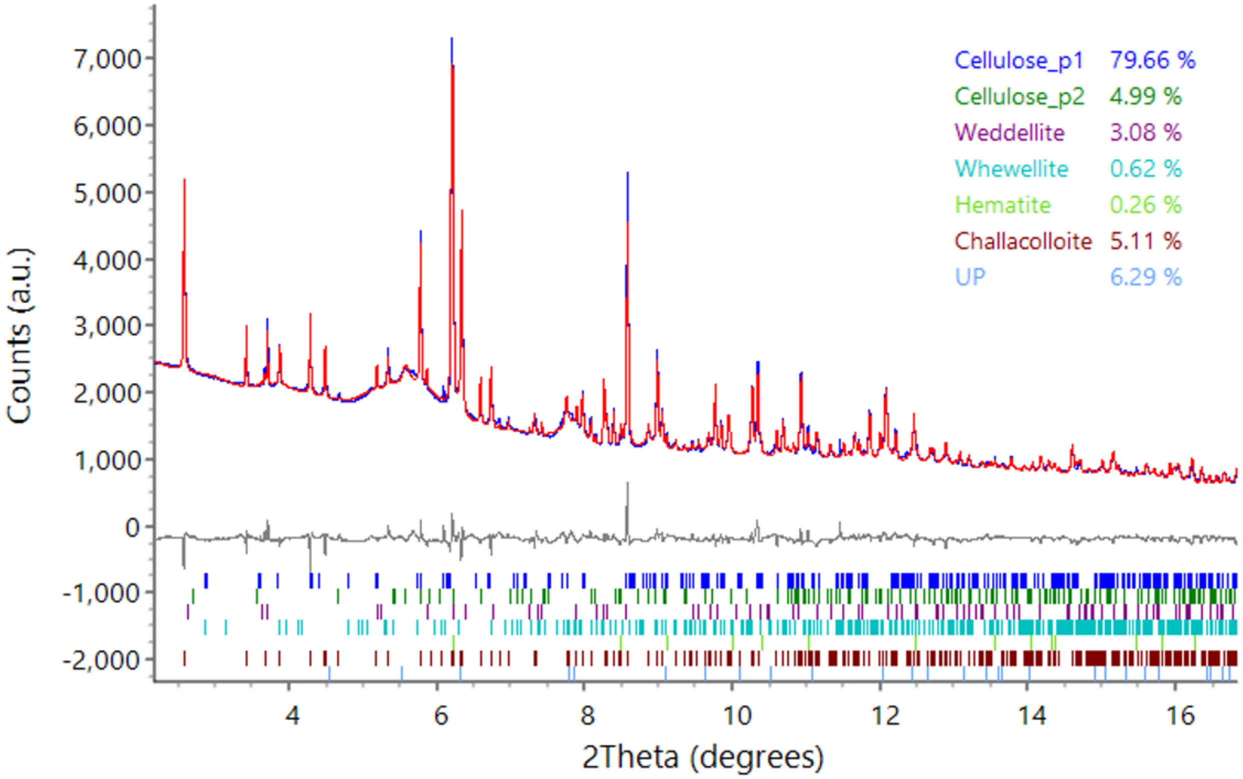


PAP-6 Deceased X-19250 Z-28750 ($R_{wp}$ 1.91%, $R_{exp}$ 1.85%)

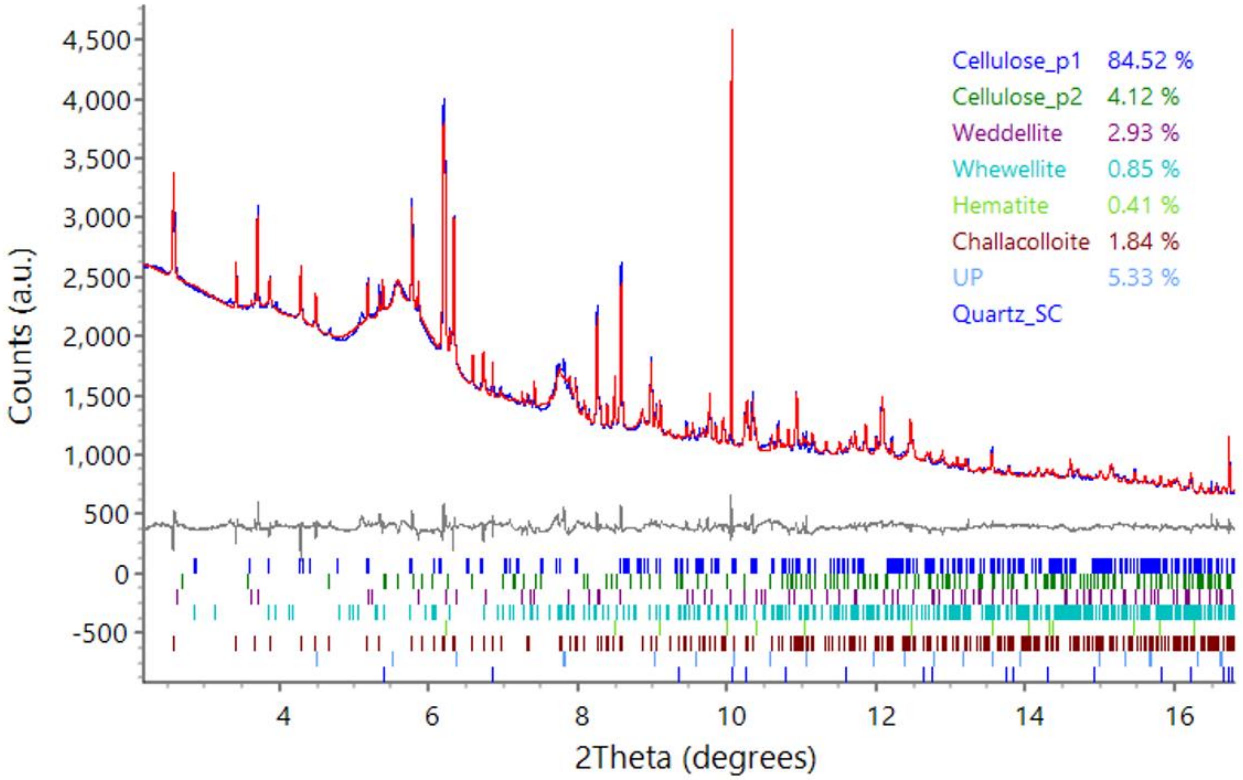

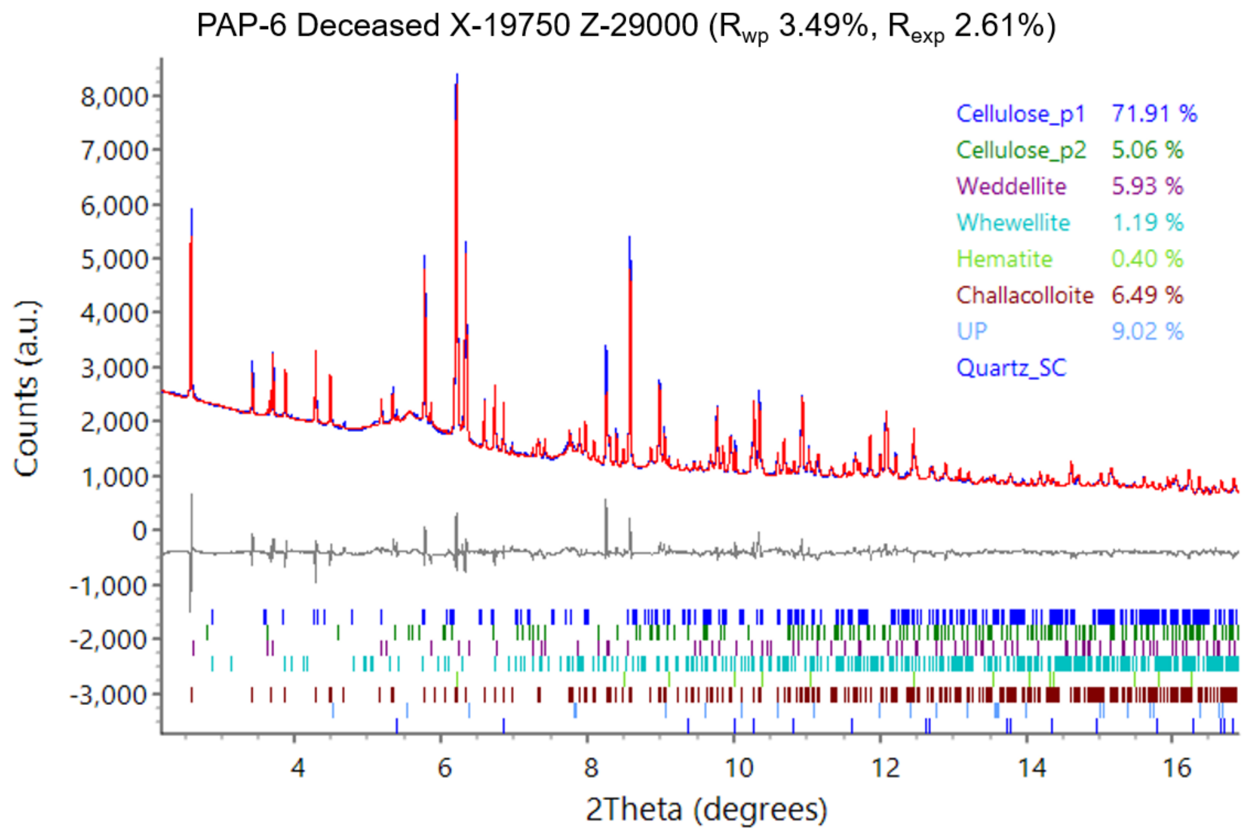
PAP-6 Deceased X-19750 Z-29000 ($R_{wp}$ 3.49%, $R_{exp}$ 2.61%)
Cellulose_p1 71.91 %
Cellulose_p2 5.06 %
Weddellite 5.93 %
Whewellite 1.19 %
Hematite 0.40 %
Challacolloite 6.49 %
UP 9.02 %
Quartz_SC
Counts (a.u.)
8,000
7,000
6,000
5,000
4,000
3,000
2,000
1,000
0
-1,000
-2,000
-3,000
4
6
8
10
12
14
16
2Theta (degrees)

**Refinement results – Column**

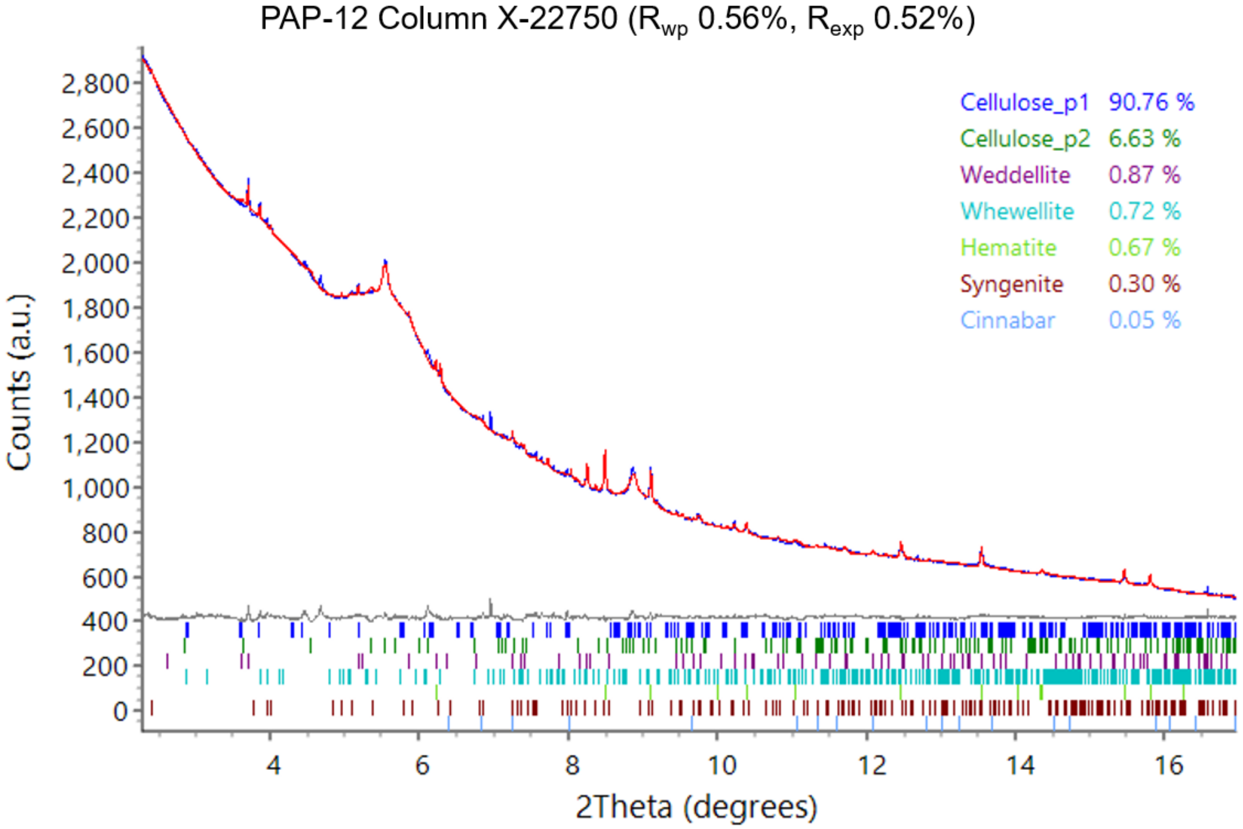


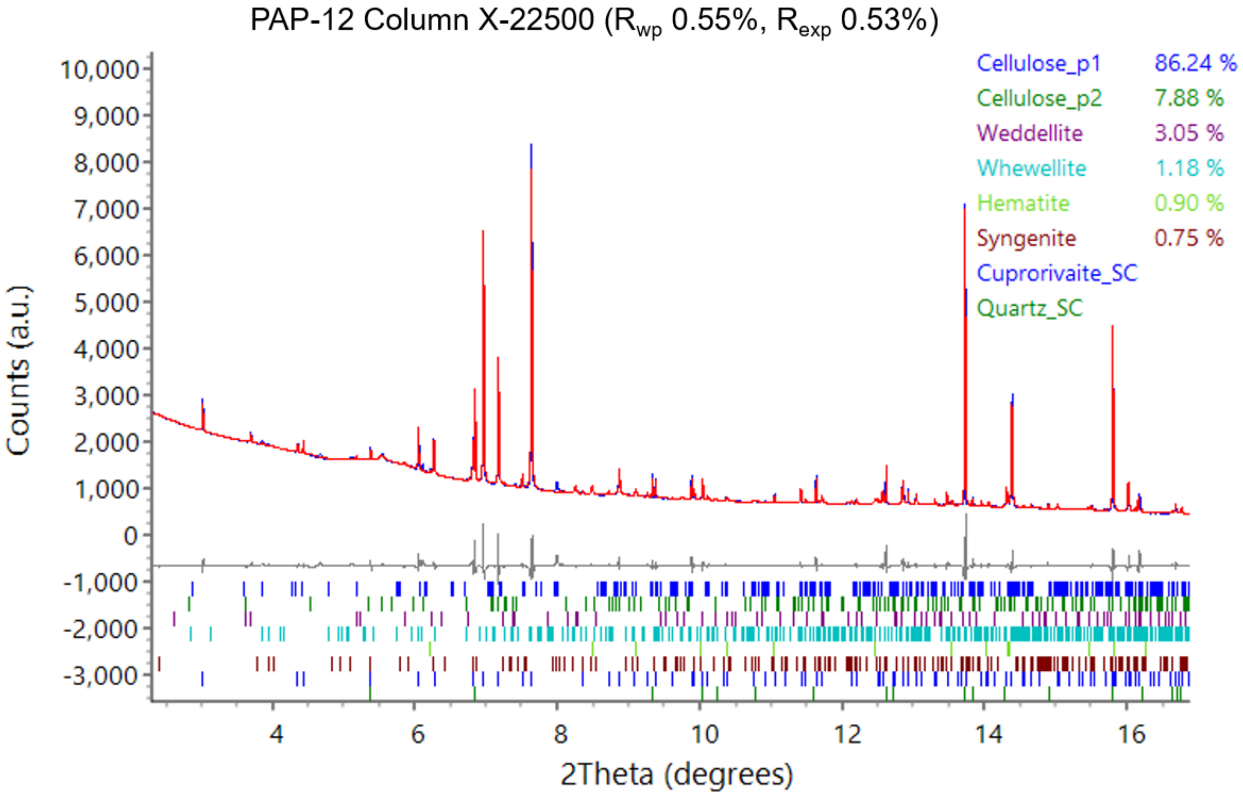

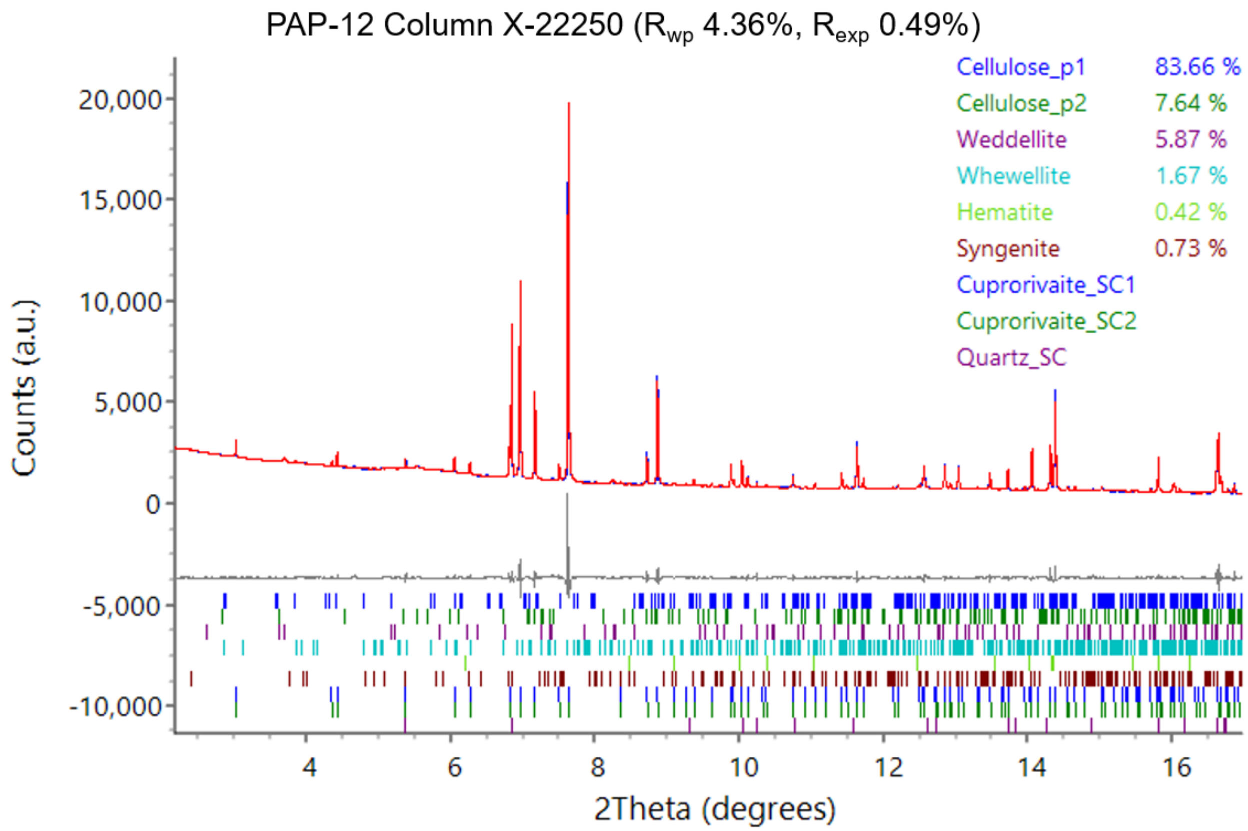
PAP-12 Column X-22250 ($R_{wp}$ 4.36%, $R_{exp}$ 0.49%)
Cellulose_p1 83.66 %
Cellulose_p2 7.64 %
Weddellite 5.87 %
Whewellite 1.67 %
Hematite 0.42 %
Syngenite 0.73 %
Cuprorivaite_SC1
Cuprorivaite_SC2
Quartz_SC
Counts (a.u.)
2Theta (degrees)

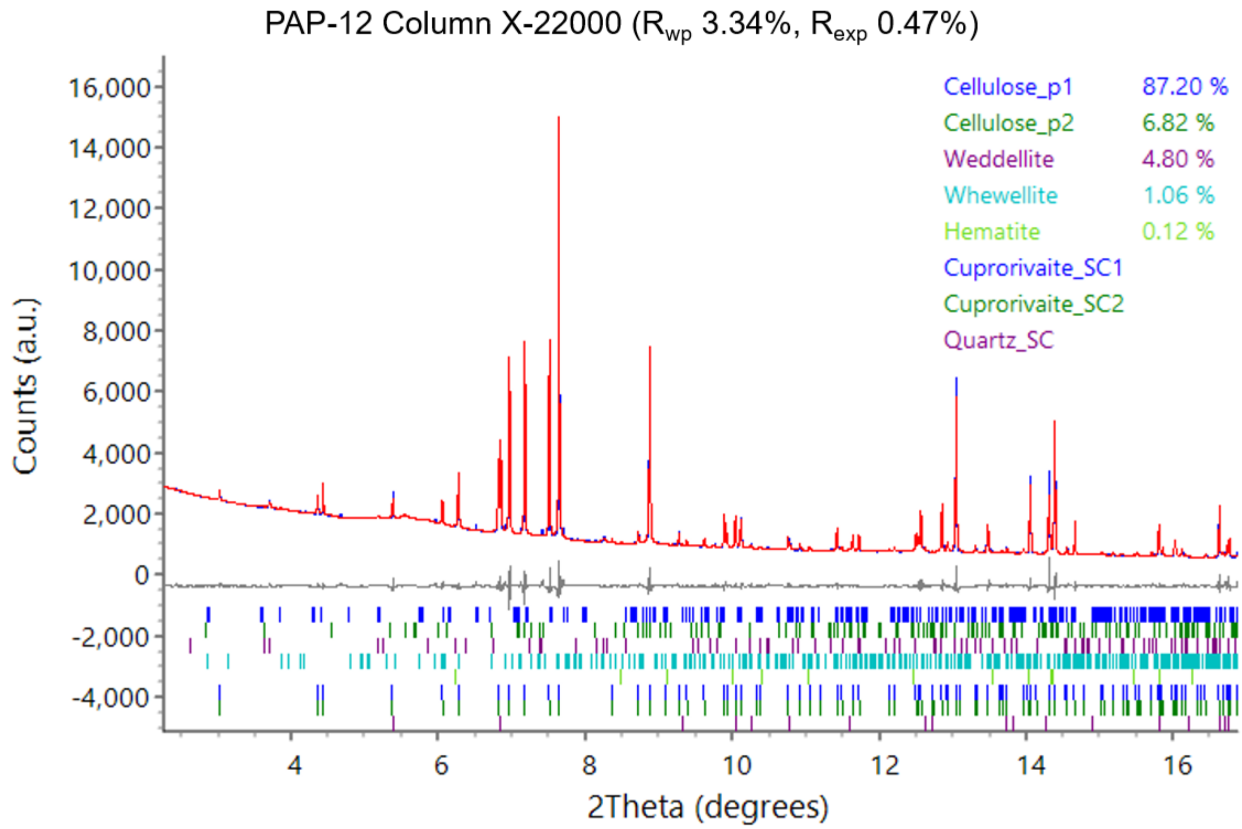
PAP-12 Column X-22000 ($R_{wp}$ 3.34%, $R_{exp}$ 0.47%)
Cellulose_p1 87.20 %
Cellulose_p2 6.82 %
Weddellite 4.80 %
Whewellite 1.06 %
Hematite 0.12 %
Cuprorivaite_SC1
Cuprorivaite_SC2
Quartz_SC
Counts (a.u.)
2Theta (degrees)

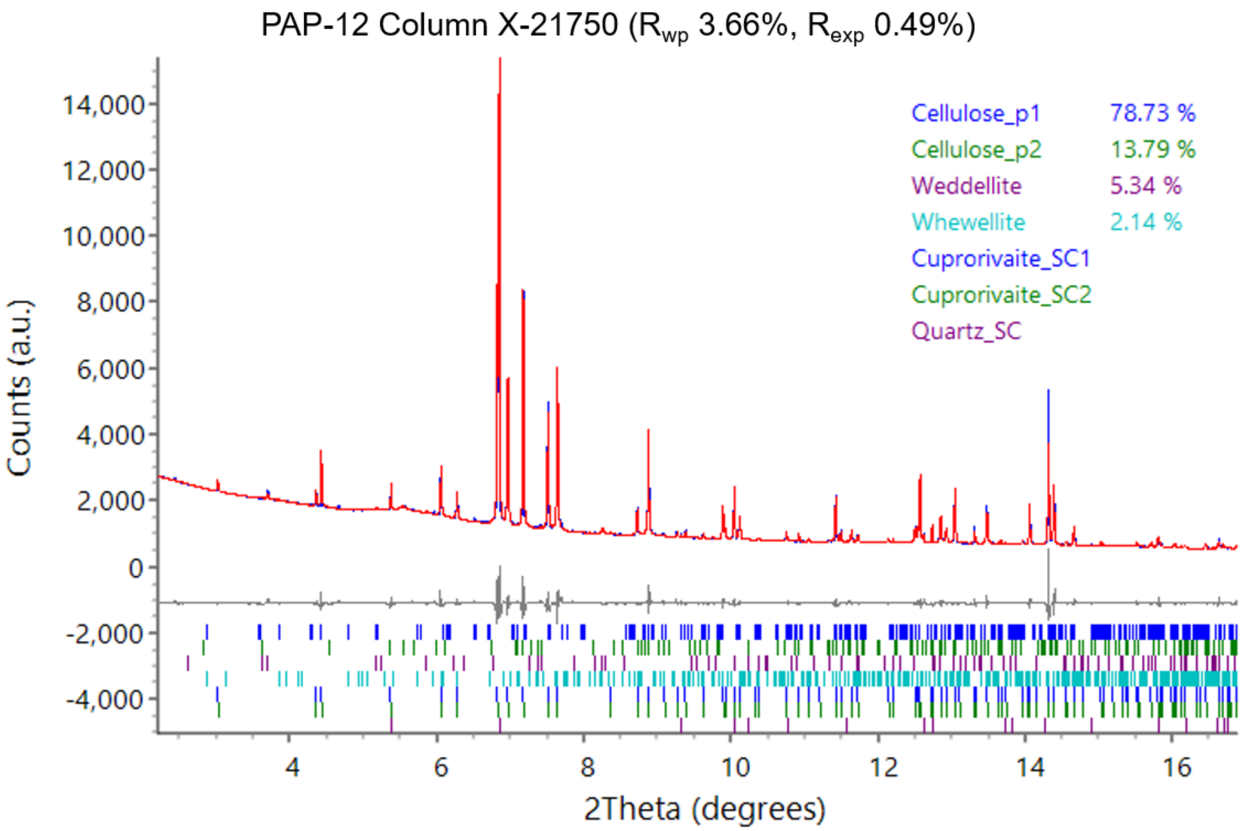
PAP-12 Column X-21750 ($R_{wp}$ 3.66%, $R_{exp}$ 0.49%)
Cellulose_p1 78.73 %
Cellulose_p2 13.79 %
Weddellite 5.34 %
Whewellite 2.14 %
Cuprorivaite_SC1
Cuprorivaite_SC2
Quartz_SC
Counts (a.u.)
2Theta (degrees)

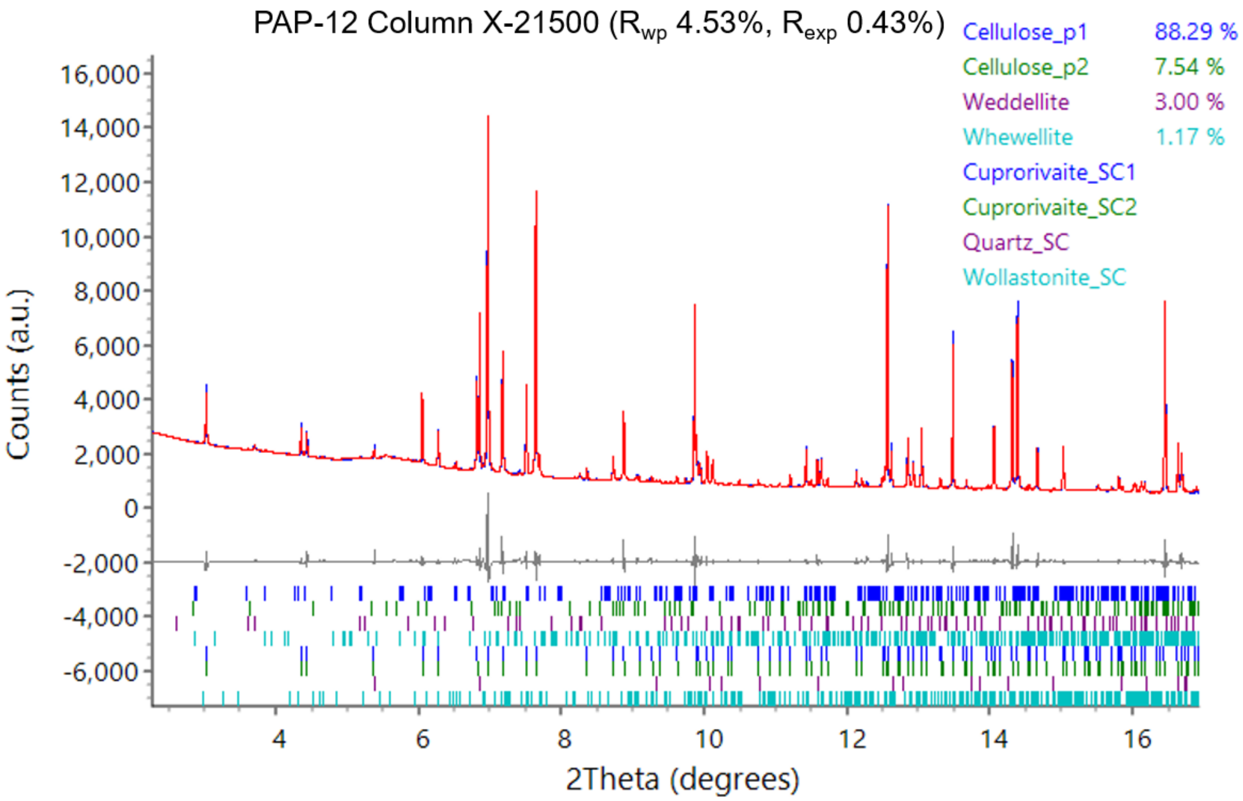
PAP-12 Column X-21500 ($R_{wp}$ 4.53%, $R_{exp}$ 0.43%)
Cellulose_p1 88.29 %
Cellulose_p2 7.54 %
Weddellite 3.00 %
Whewellite 1.17 %
Cuprorivaite_SC1
Cuprorivaite_SC2
Quartz_SC
Wollastonite_SC
Counts (a.u.)
2Theta (degrees)

PAP-12 Column X-21250 ($R_{wp}$ 3.83%, $R_{exp}$ 0.47%)

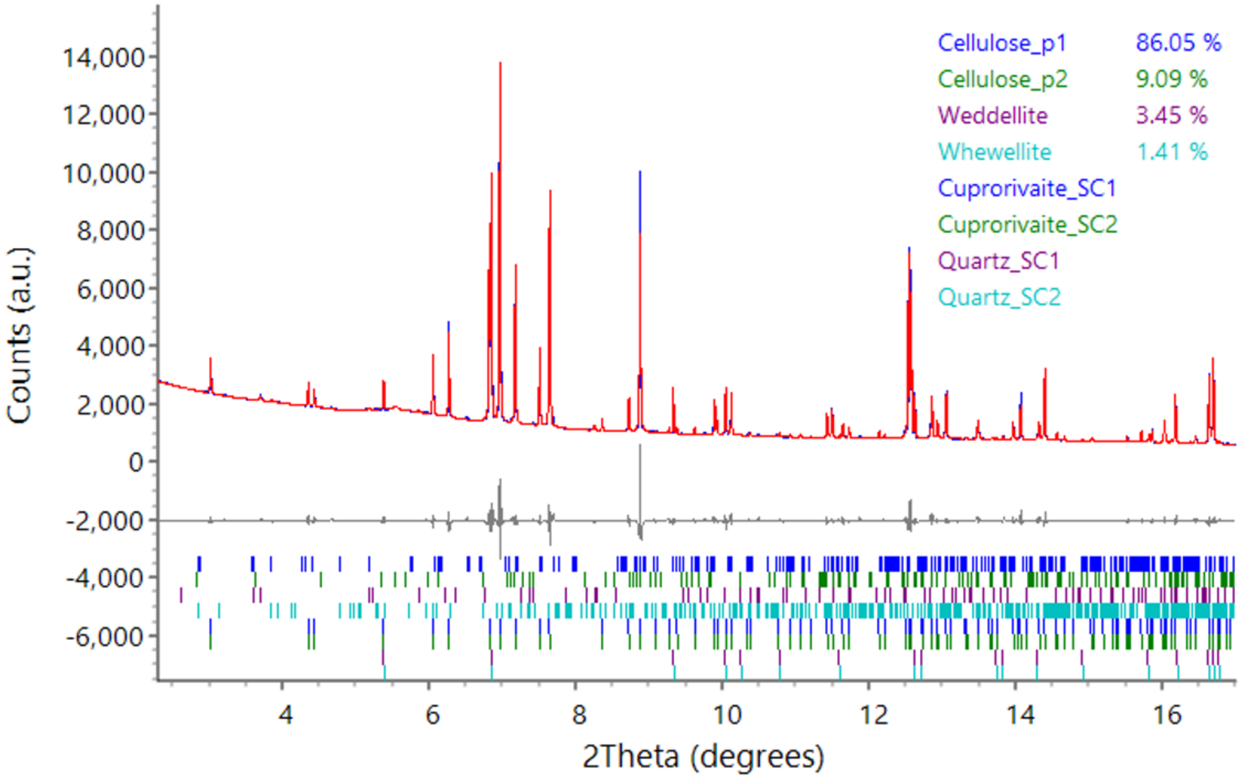


PAP-12 Column X-21000 ($R_{wp}$ 3.17%, $R_{exp}$ 0.50%)

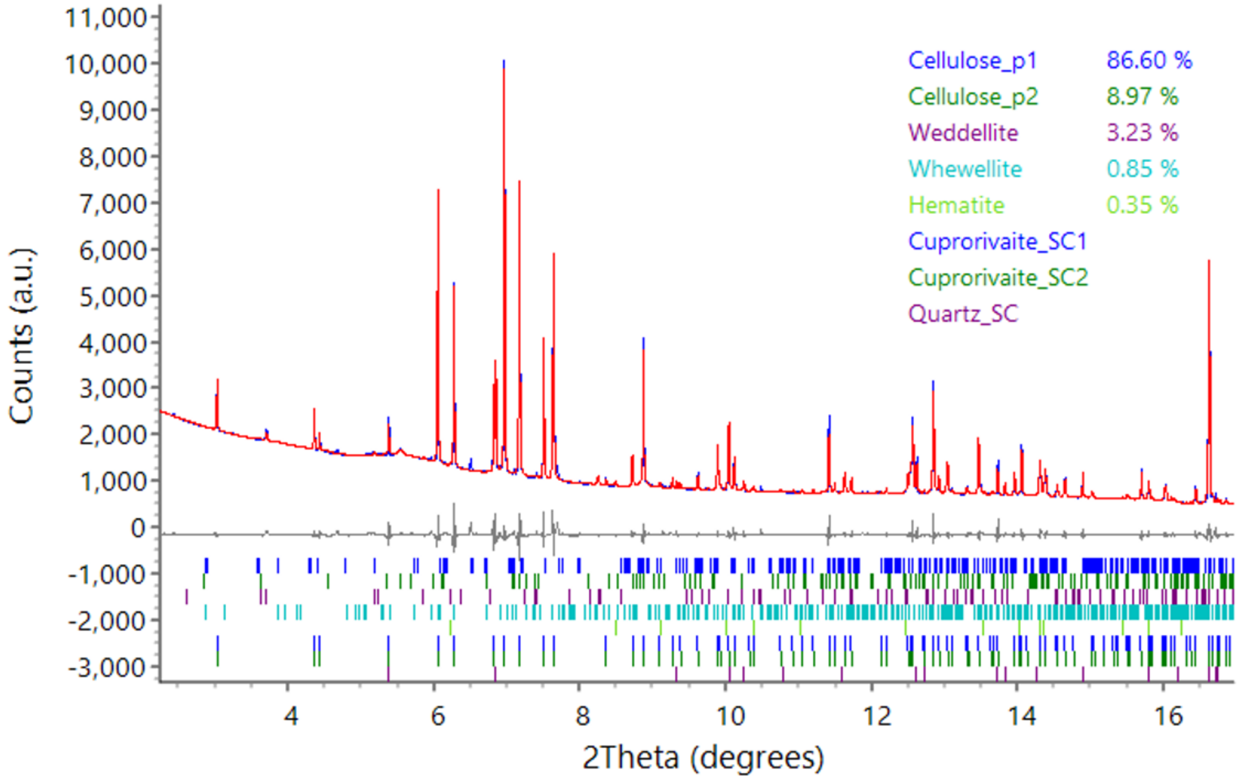

PAP-12 Column X-20750 ($R_{wp}$ 2.01%, $R_{exp}$ 0.50%)

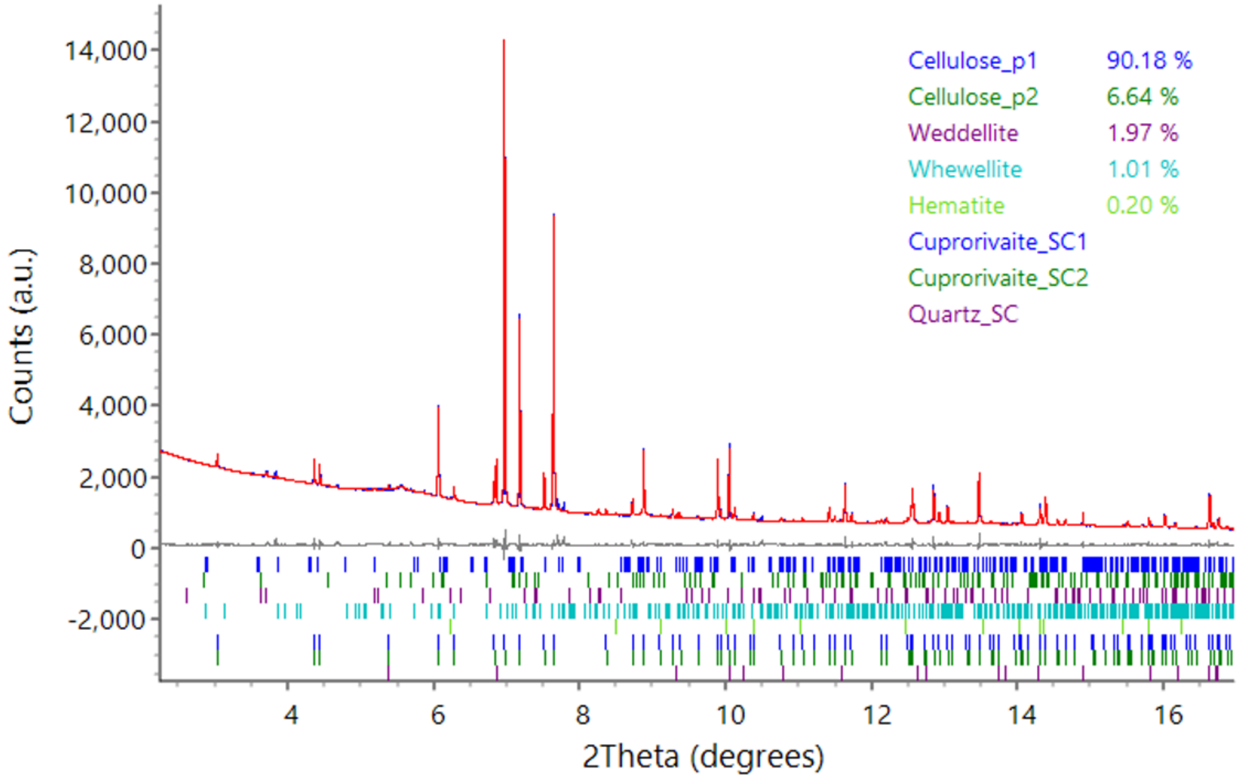


PAP-12 Column X-20500 ($R_{wp}$ 0.45%, $R_{exp}$ 0.42%)

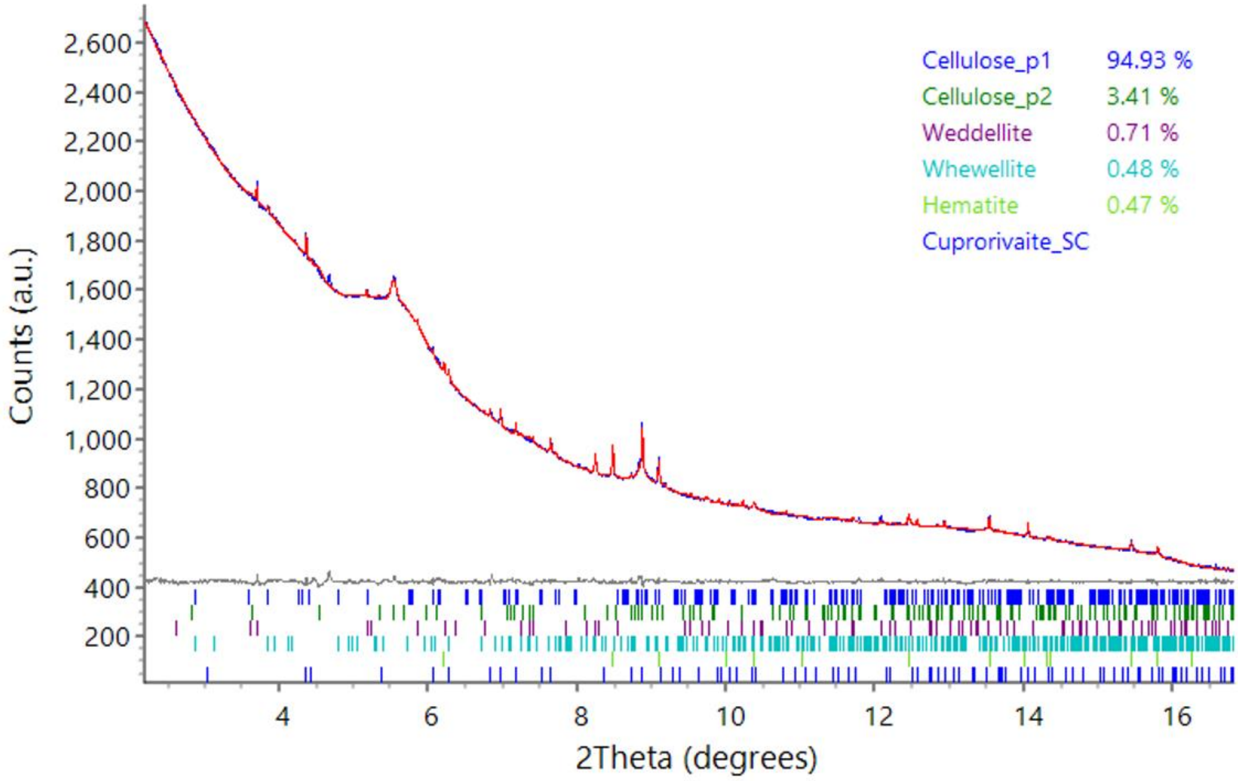

PAP-12 Column X-20250 ($R_{wp}$ 0.76%, $R_{exp}$ 0.56%)

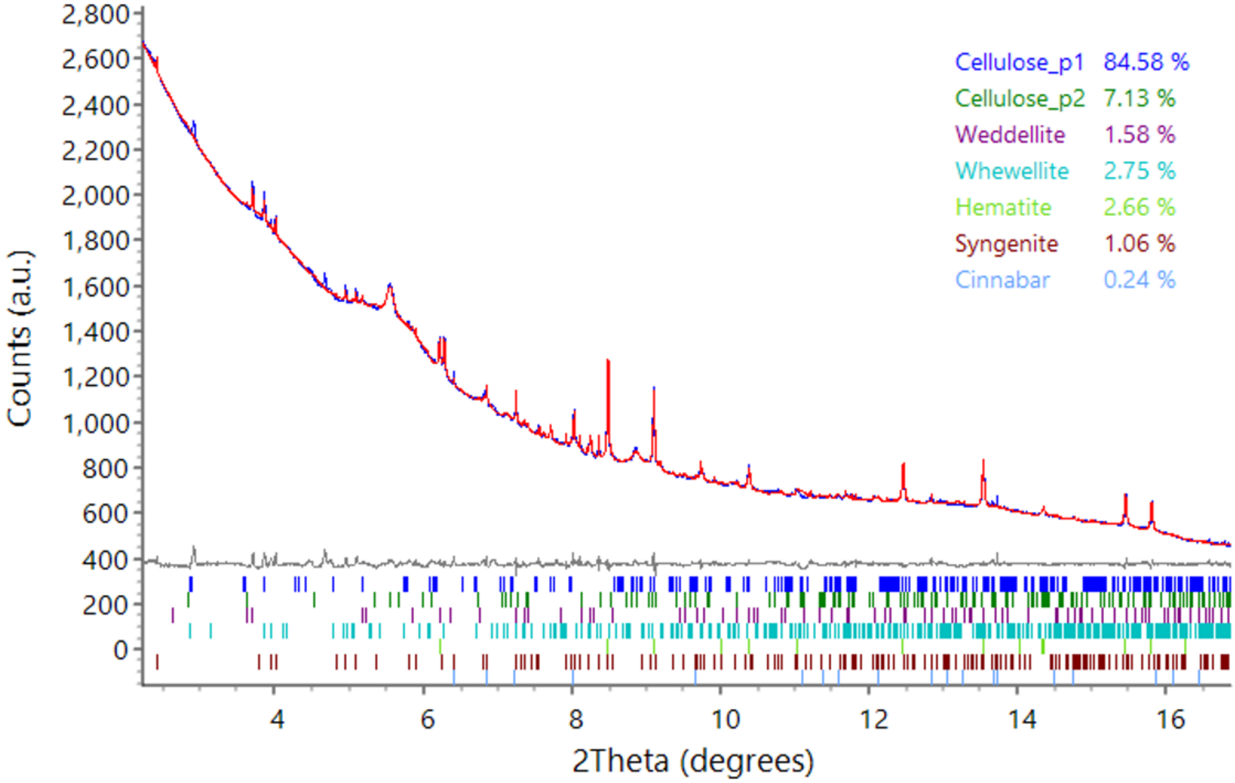


PAP-12 Column X-20000 ($R_{wp}$ 0.71%, $R_{exp}$ 0.54%)

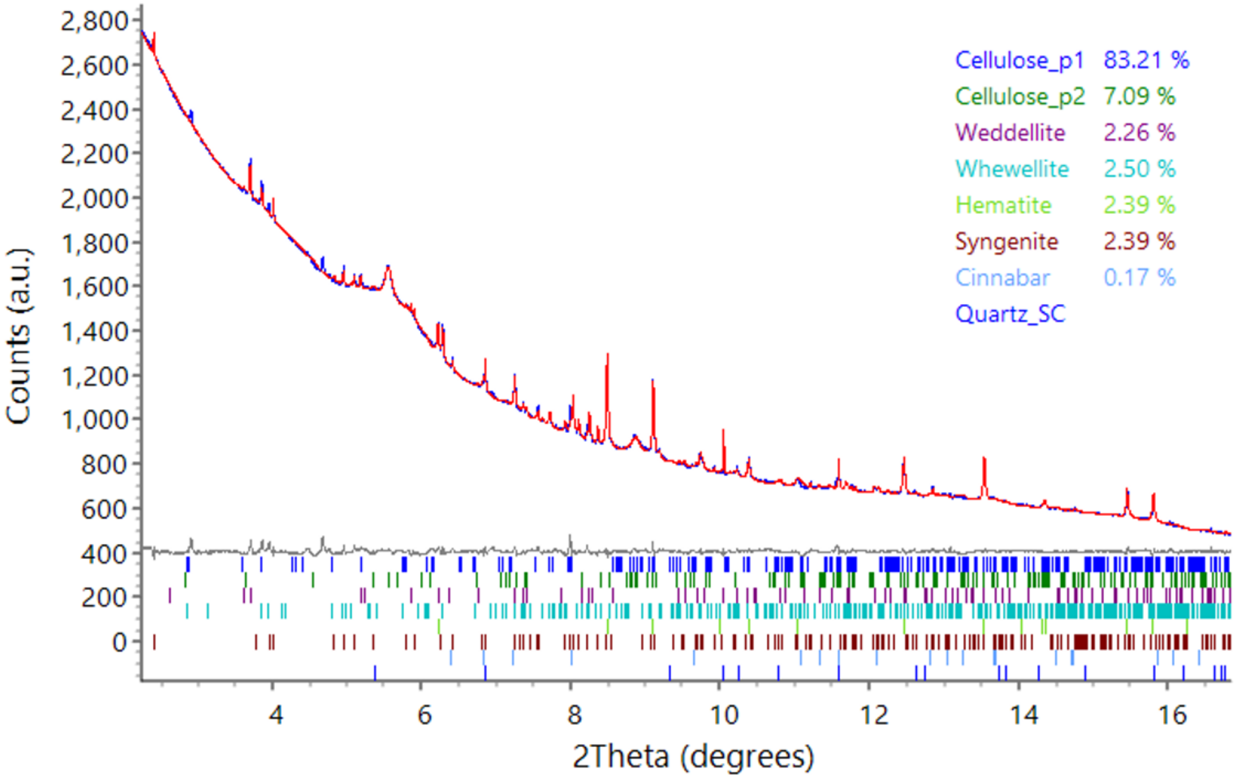

PAP-12 Column X-19750 ($R_{wp}$ 1.01%, $R_{exp}$ 0.56%)

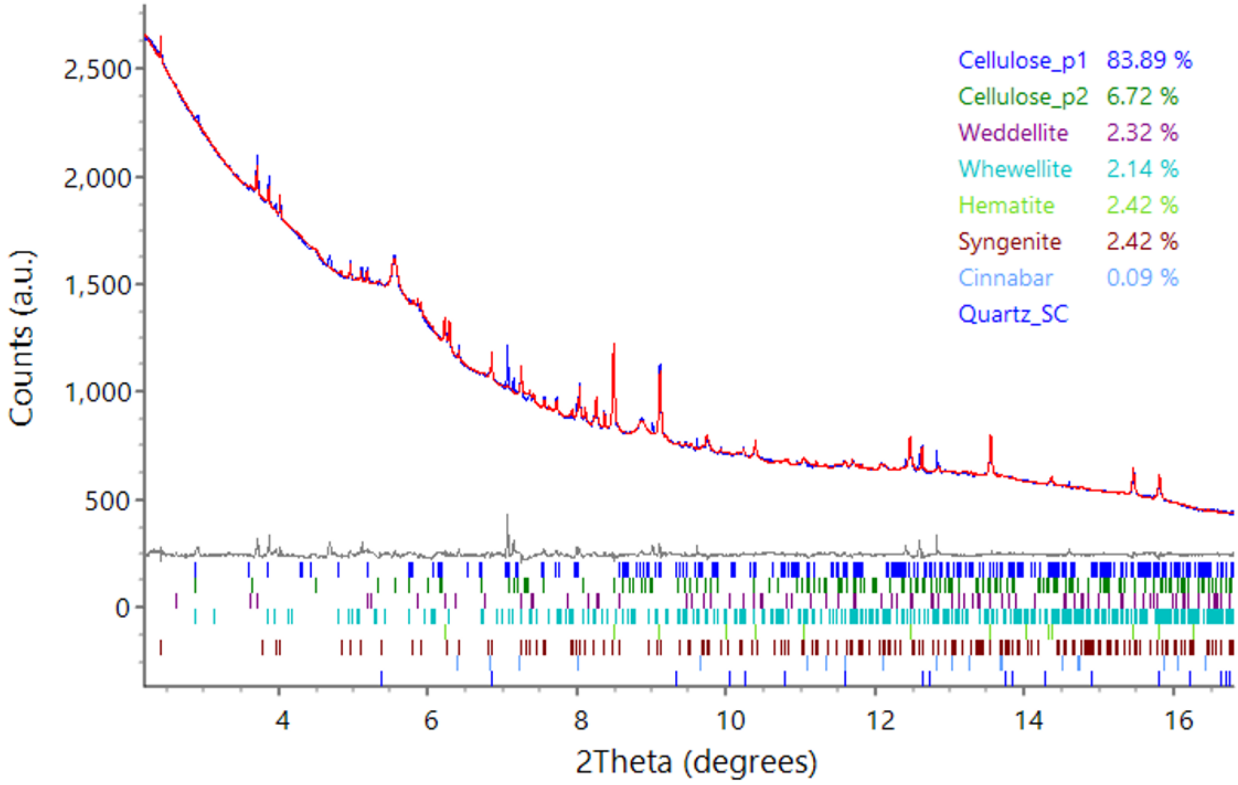


PAP-12 Column X-19500 ($R_{wp}$ 0.70%, $R_{exp}$ 0.56%)

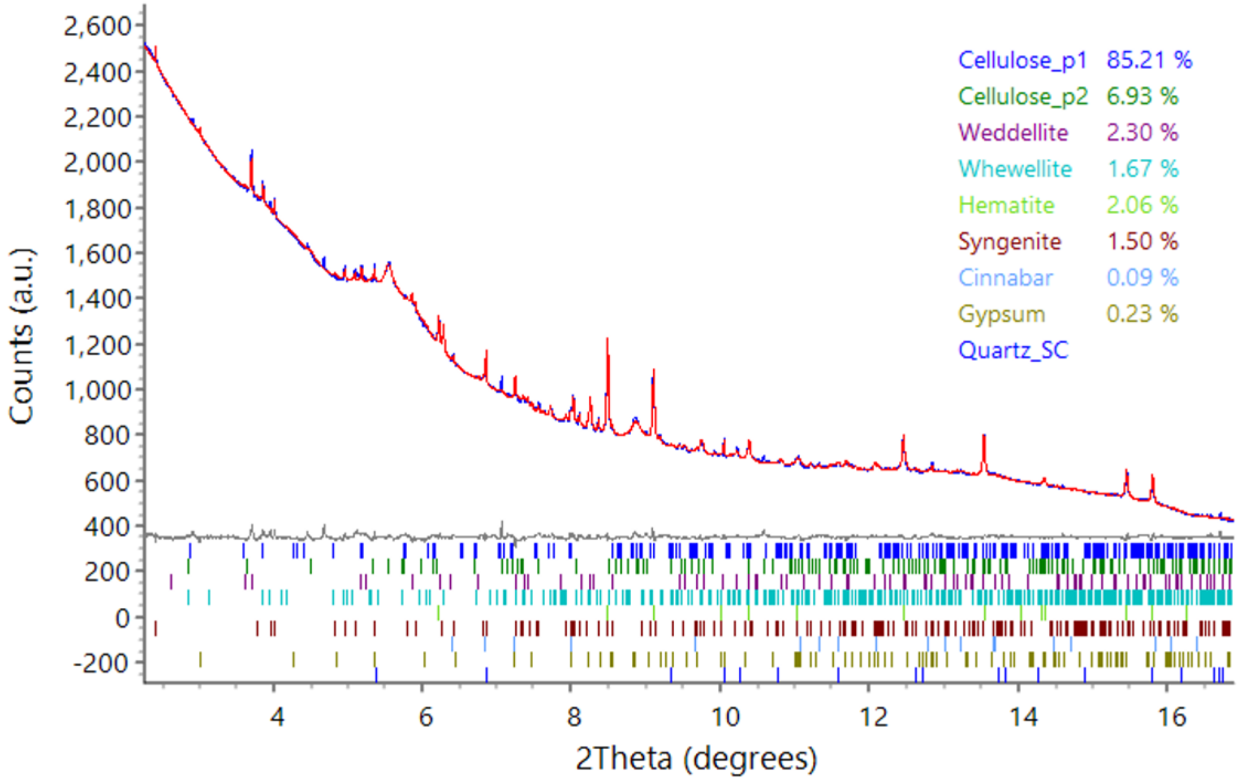

PAP-12 Column X-19250 ($R_{wp}$ 0.78%, $R_{exp}$ 0.55%)

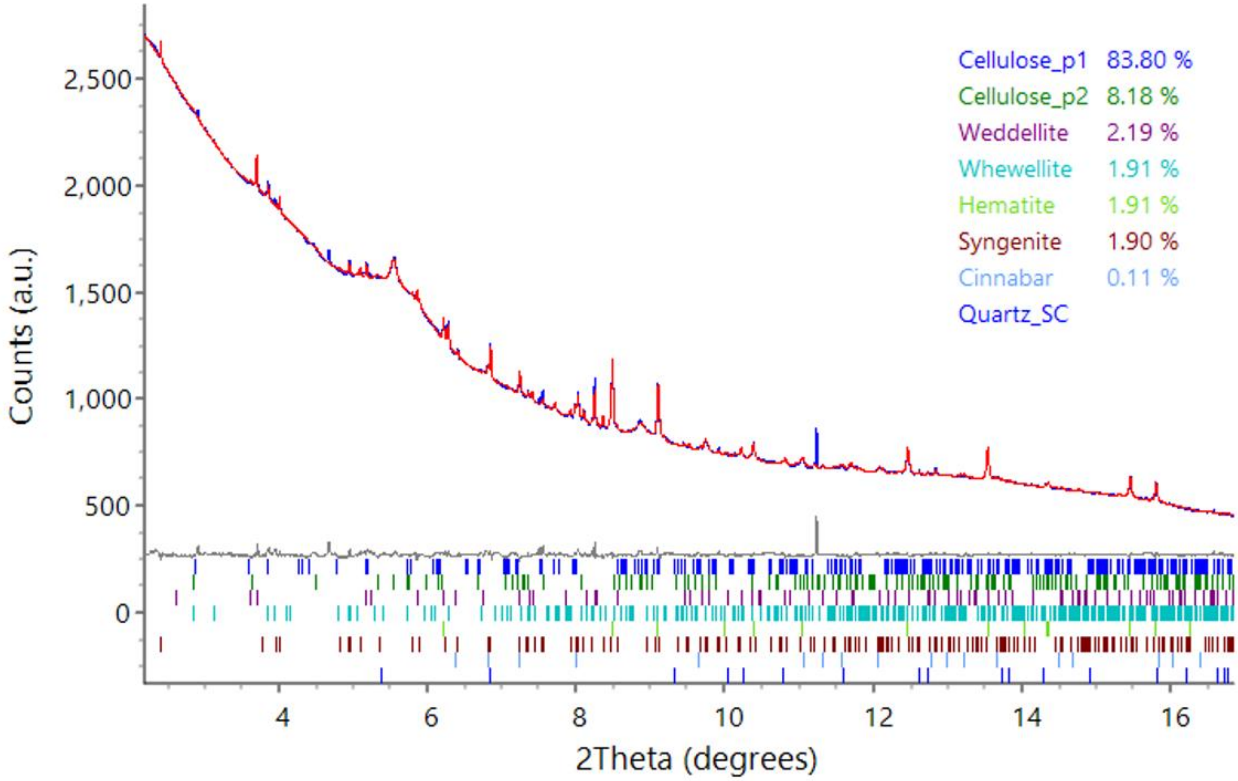


PAP-12 Column X-19000 ($R_{wp}$ 0.63%, $R_{exp}$ 0.56%)

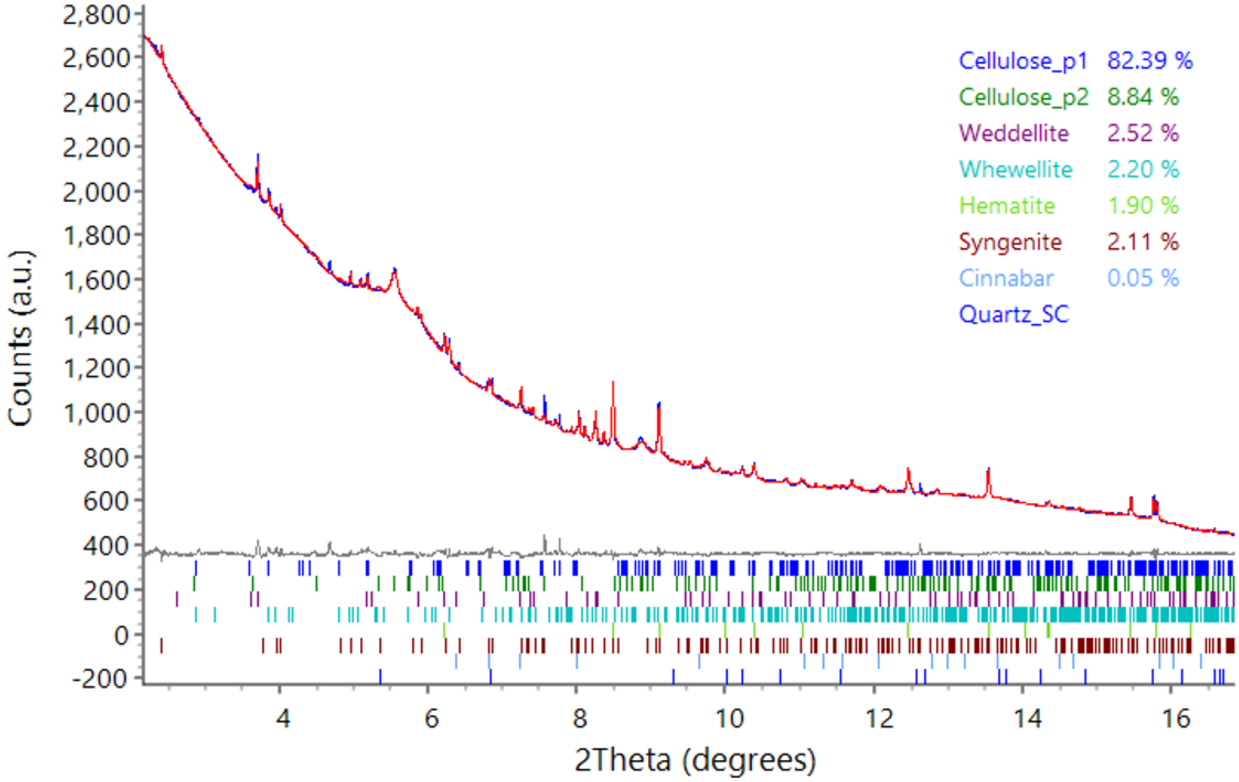

PAP-12 Column X-18750 ($R_{wp}$ 0.56%, $R_{exp}$ 0.49%)

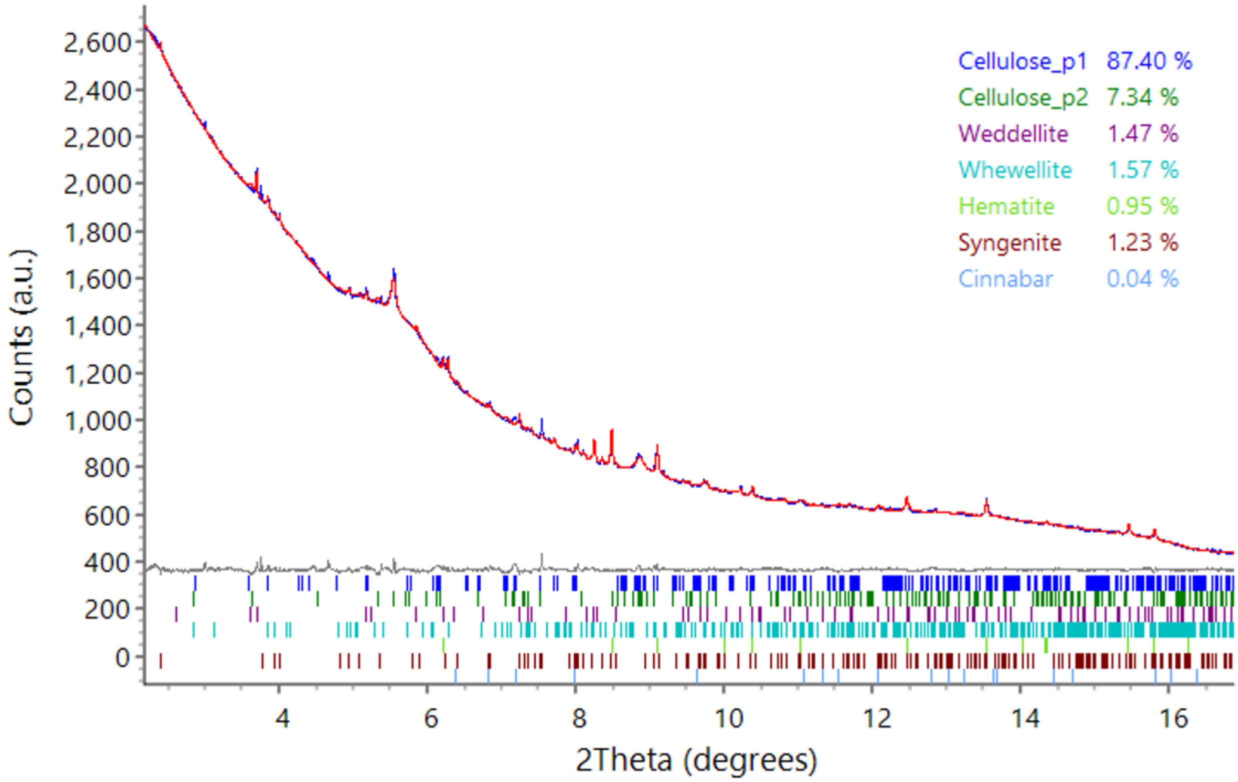


PAP-12 Column X-18250 ($R_{wp}$ 0.51%, $R_{exp}$ 0.47%)

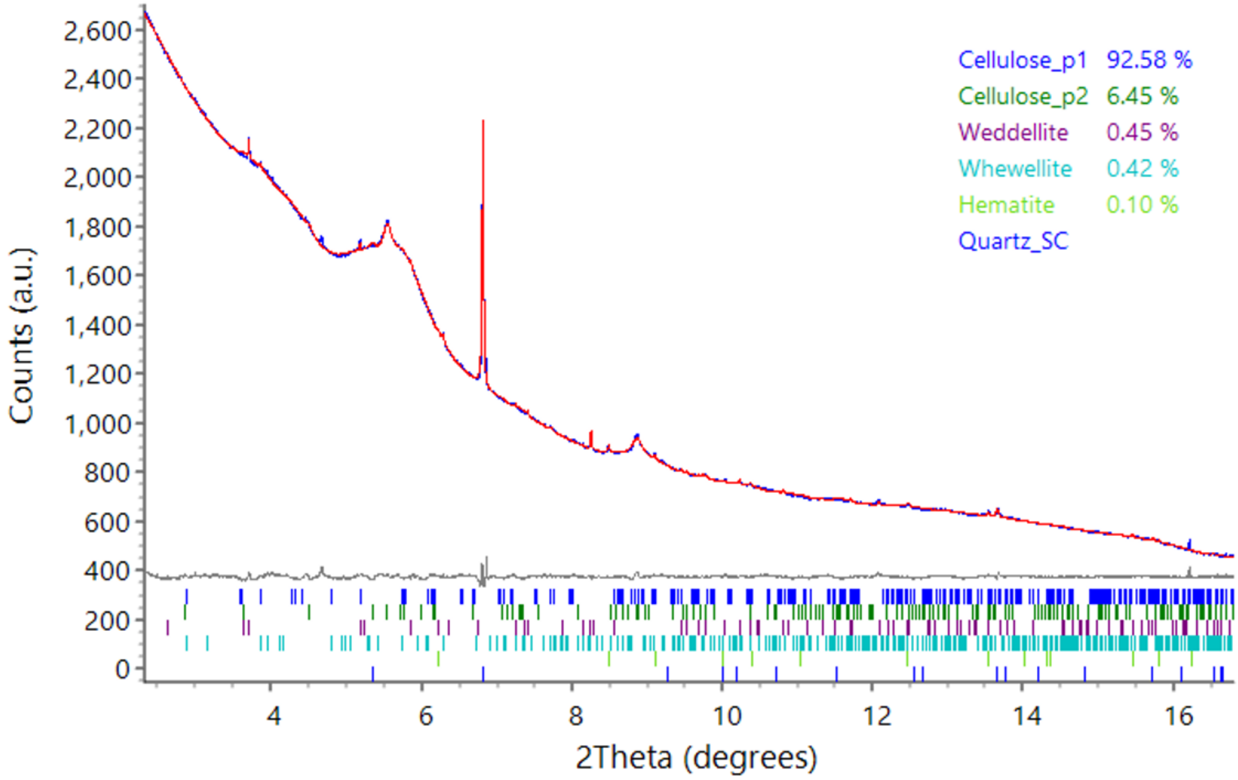

PAP-12 Column X-17750 ($R_{wp}$ 0.43%, $R_{exp}$ 0.42%)

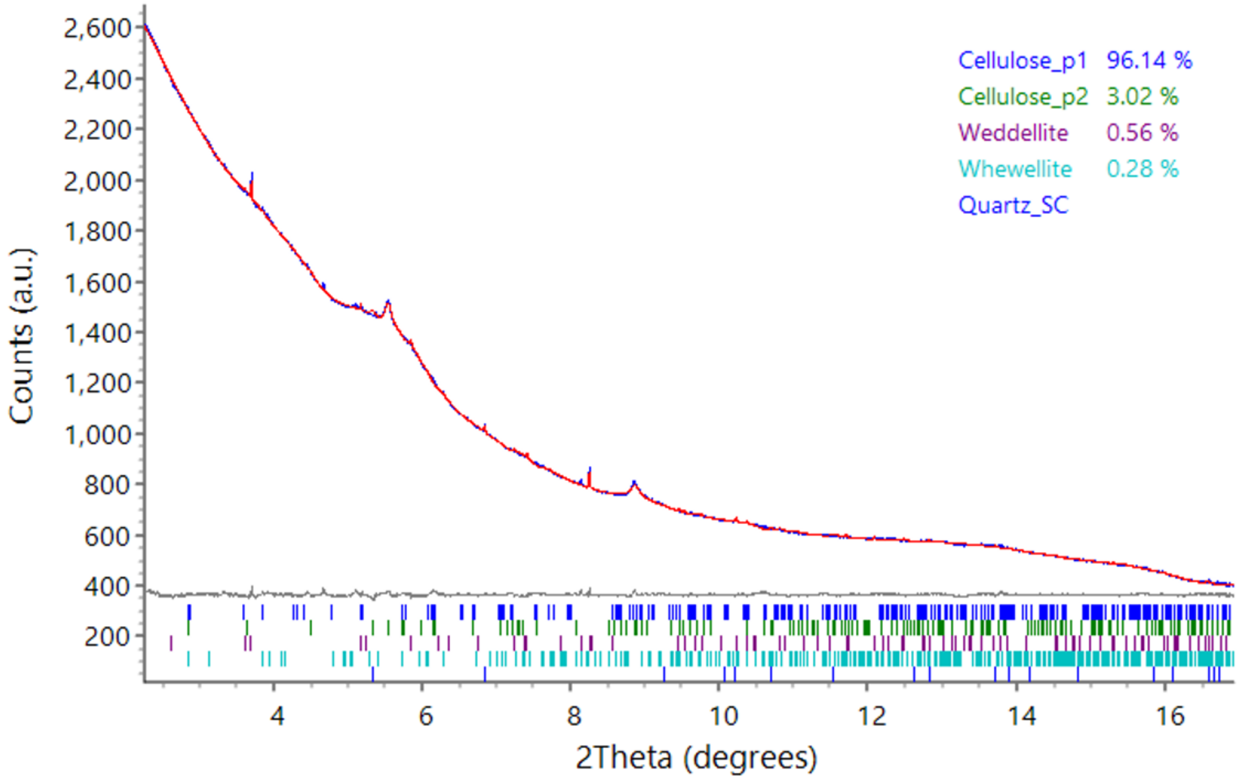


PAP-12 Column X-17500 ($R_{wp}$ 1.30%, $R_{exp}$ 0.55%)

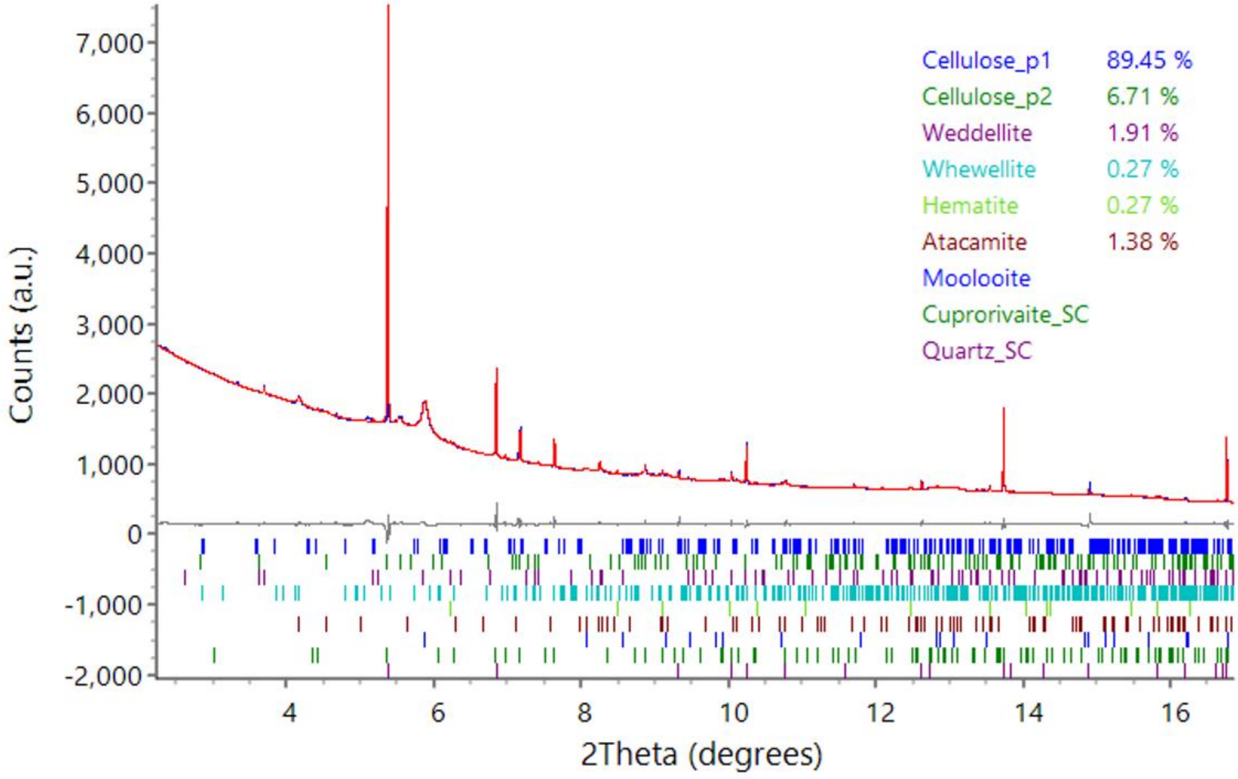

PAP-12 Column X-17250 ($R_{wp}$ 1.82%, $R_{exp}$ 0.50%)

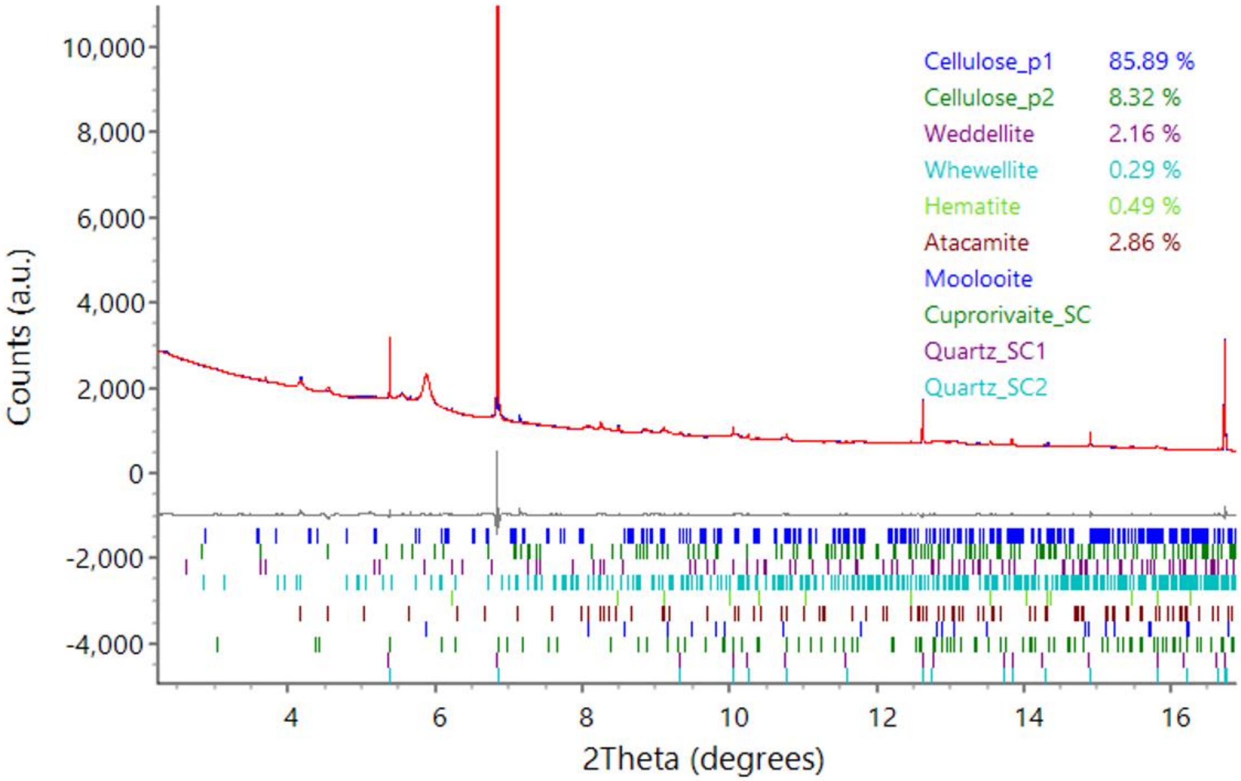


PAP-12 Column X-17000 ($R_{wp}$ 0.82%, $R_{exp}$ 0.55%)

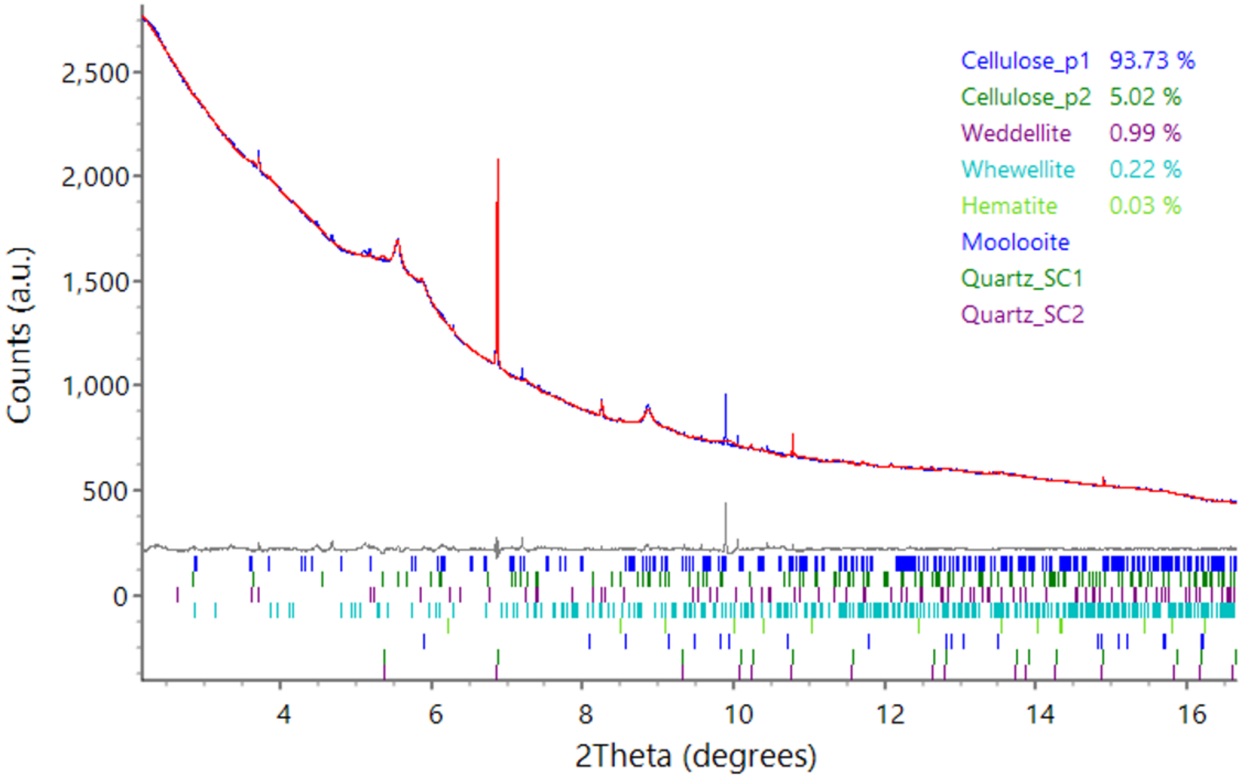

PAP-12 Column X-16750 ($R_{wp}$ 0.41%, $R_{exp}$ 0.40%)

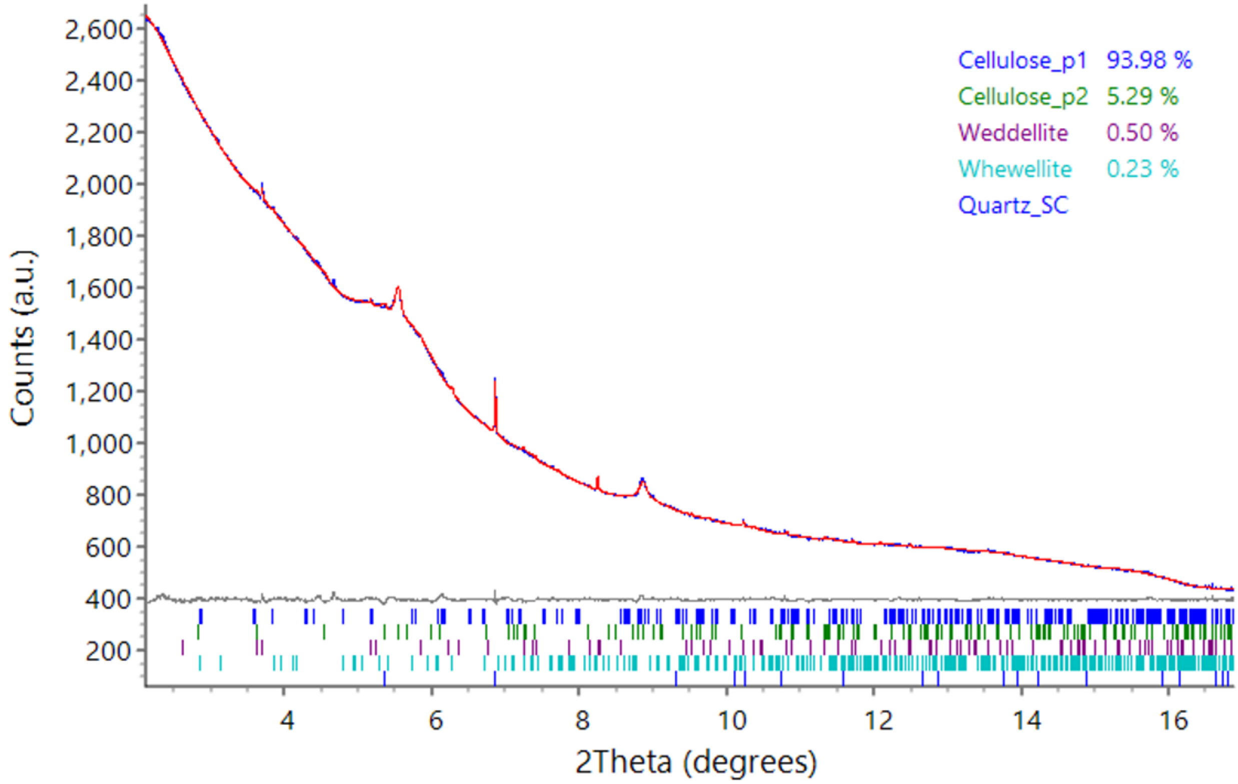


PAP-12 Column X-16500 ($R_{wp}$ 0.39%, $R_{exp}$ 0.38%)

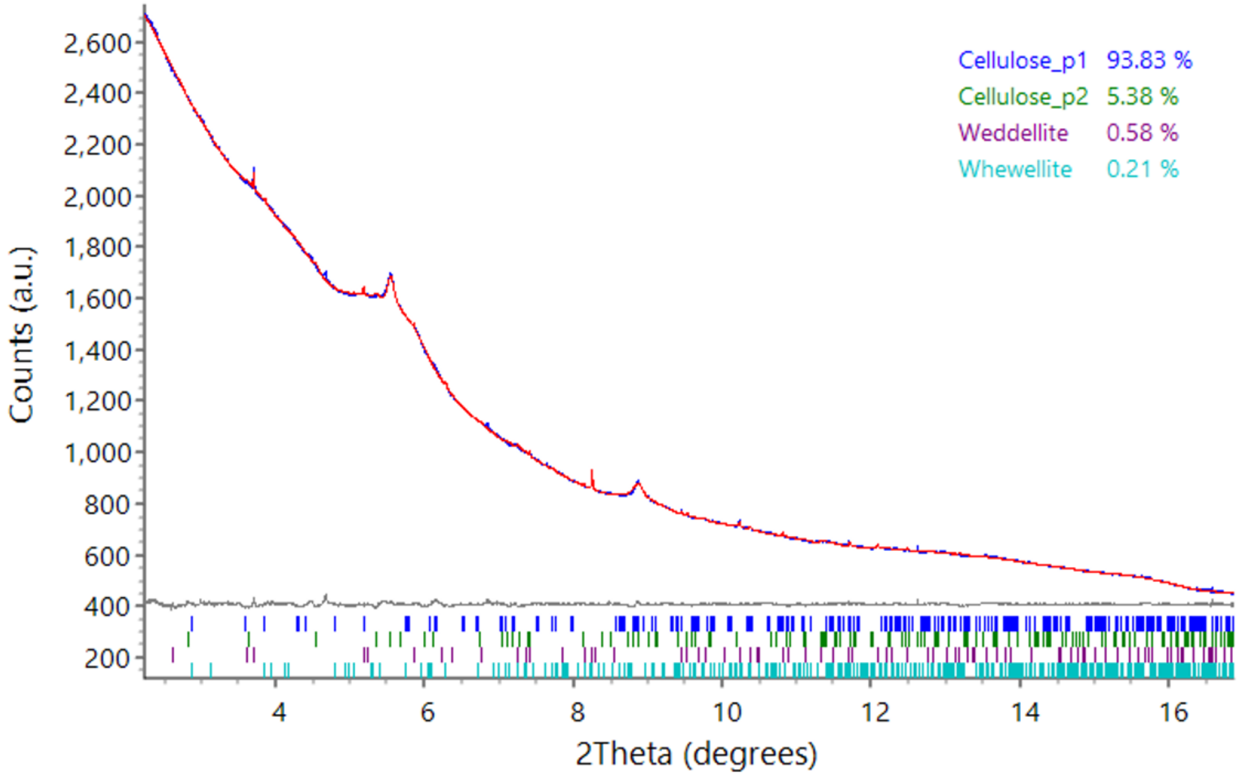

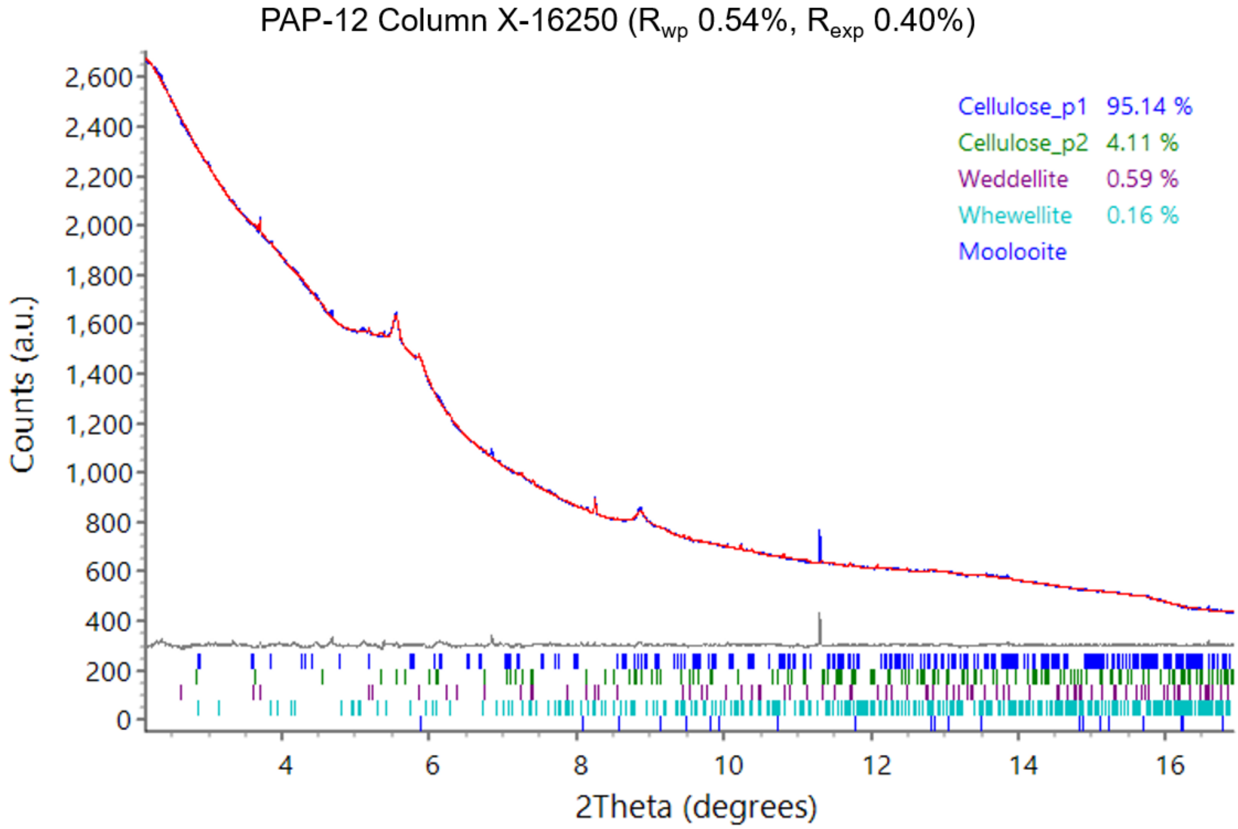
PAP-12 Column X-16250 (Rwp 0.54%, Rexp 0.40%)
Counts (a.u.)
2Theta (degrees)
Cellulose_p1 95.14 %
Cellulose_p2 4.11 %
Weddellite 0.59 %
Whewellite 0.16 %
Moolooite

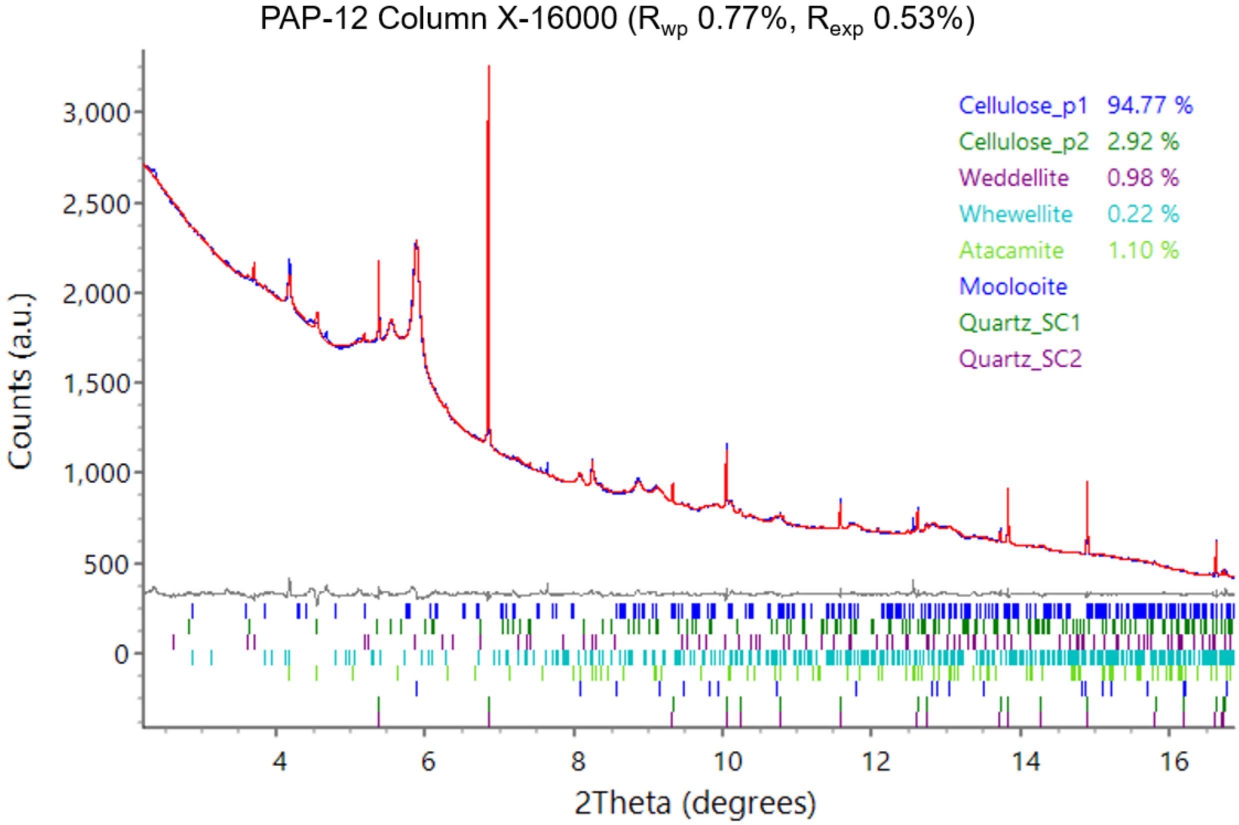
PAP-12 Column X-16000 (Rwp 0.77%, Rexp 0.53%)
Counts (a.u.)
2Theta (degrees)
Cellulose_p1 94.77 %
Cellulose_p2 2.92 %
Weddellite 0.98 %
Whewellite 0.22 %
Atacamite 1.10 %
Moolooite
Quartz_SC1
Quartz_SC2

**Refinement results – Cobra**

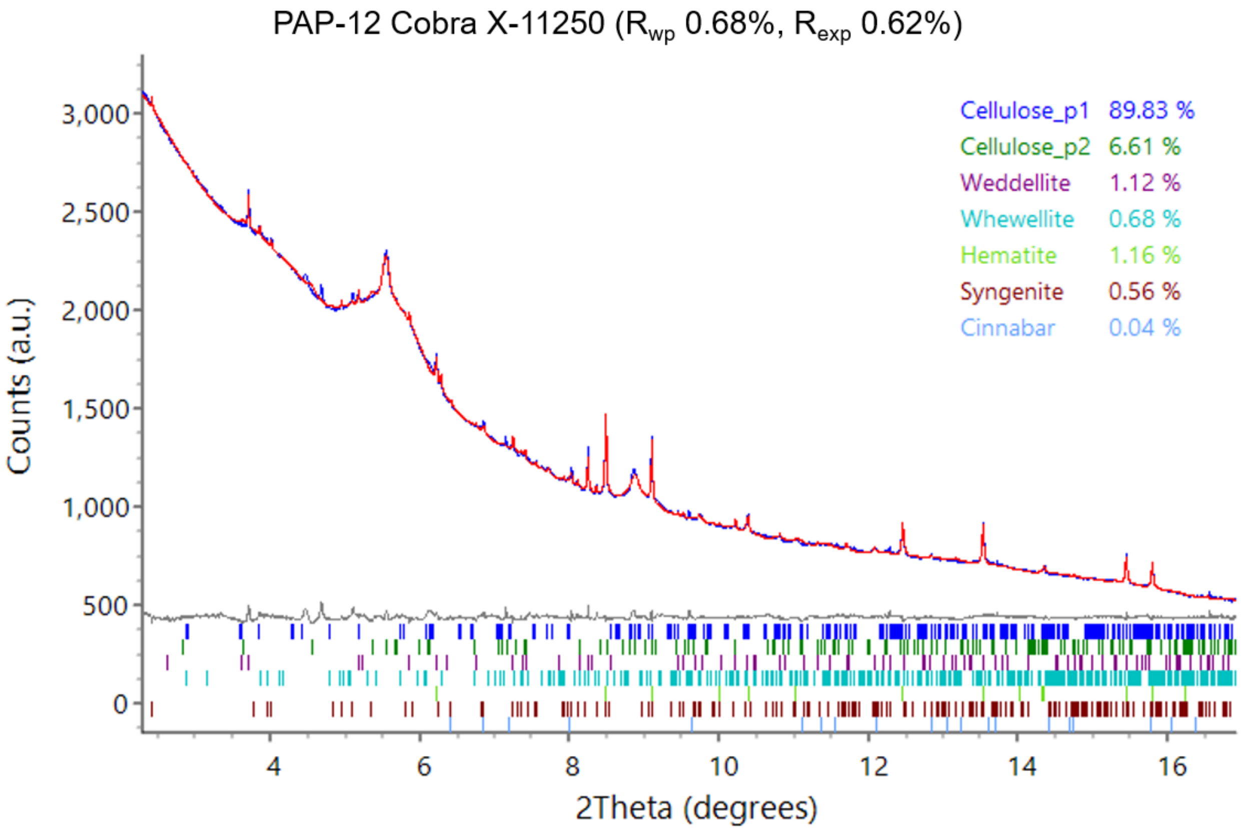


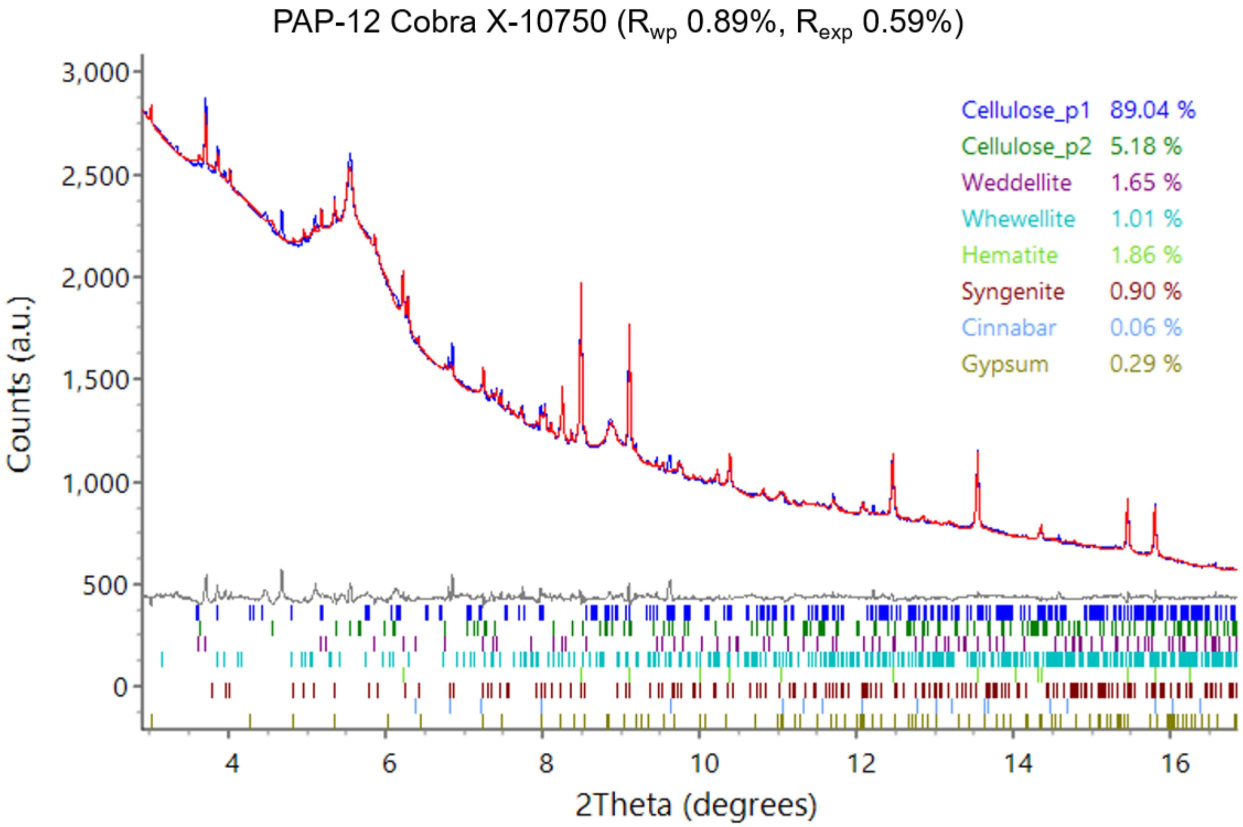

PAP-12 Cobra X-10500 ($R_{wp}$ 0.79%, $R_{exp}$ 0.53%)

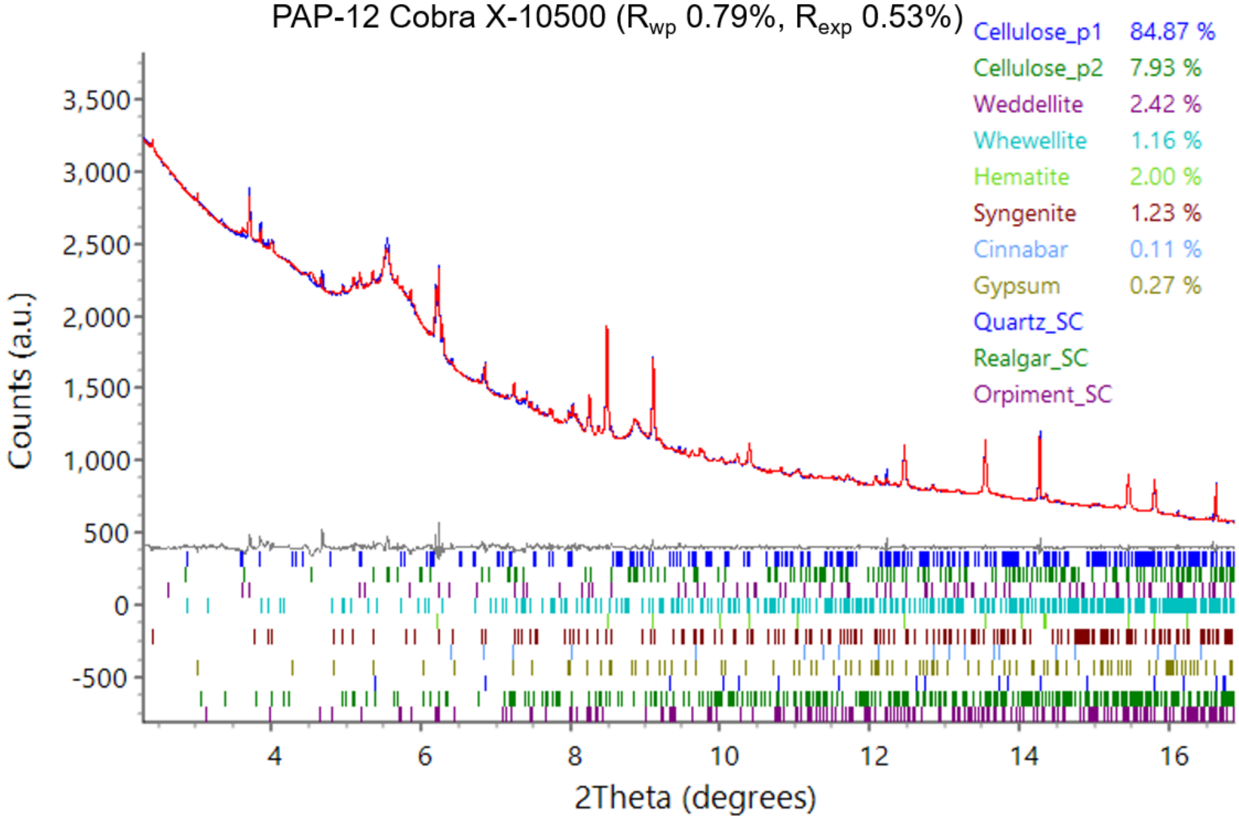


PAP-12 Cobra X-10250 ($R_{wp}$ 1.22%, $R_{exp}$ 0.56%)

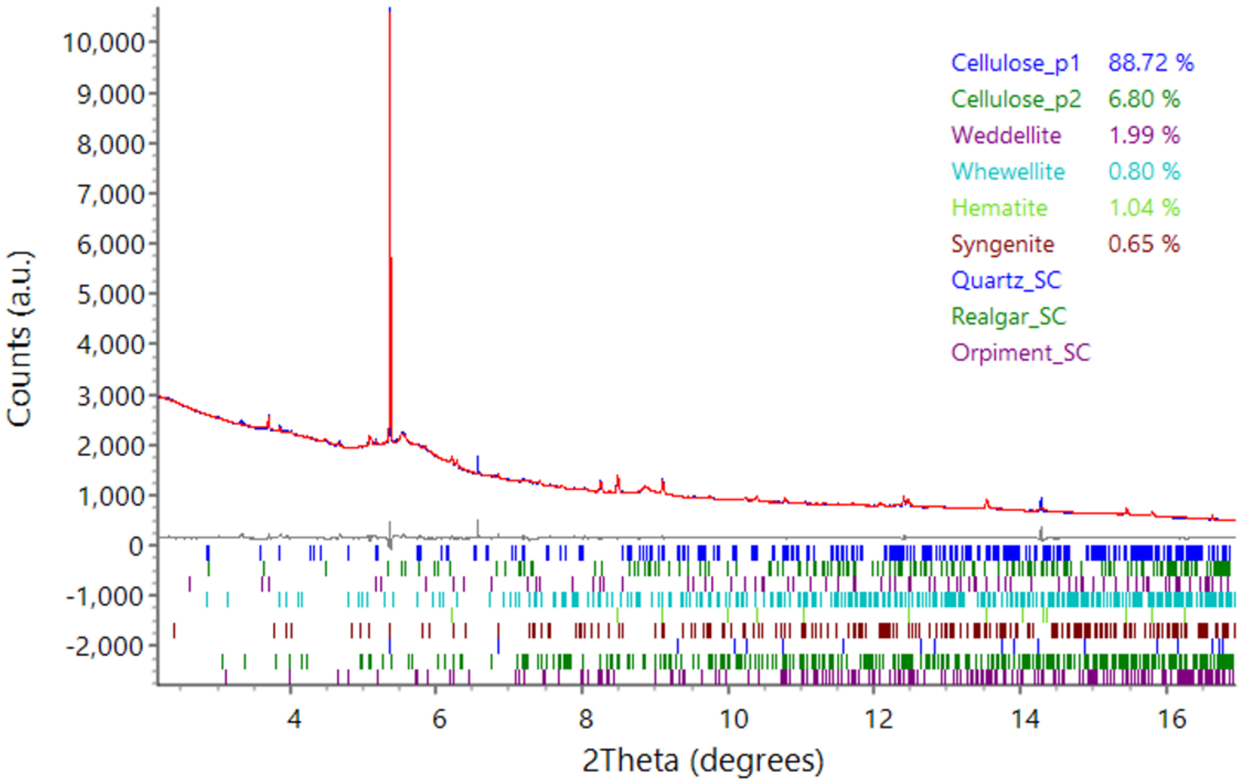

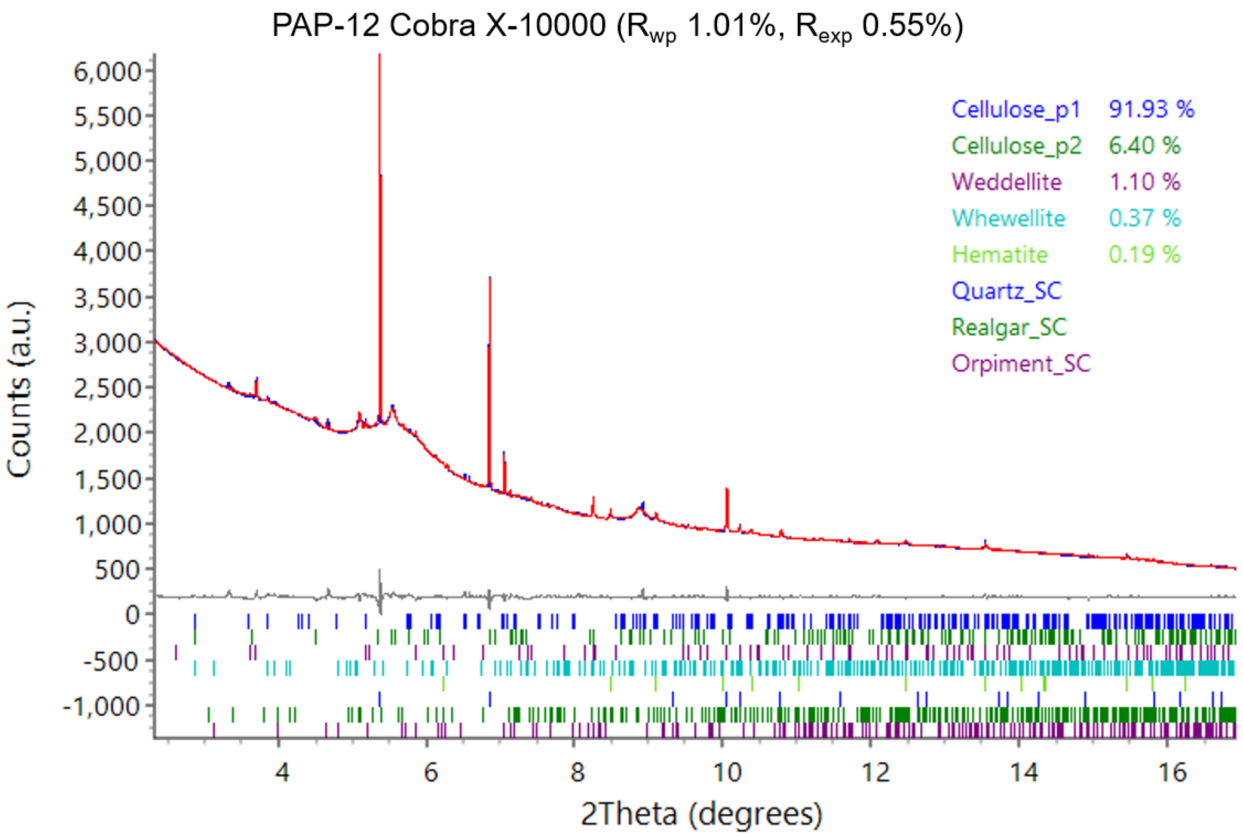
PAP-12 Cobra X-10000 ($R_{wp}$ 1.01%, $R_{exp}$ 0.55%)
Counts (a.u.)
2Theta (degrees)
Cellulose_p1 91.93 %
Cellulose_p2 6.40 %
Weddellite 1.10 %
Whewellite 0.37 %
Hematite 0.19 %
Quartz_SC
Realgar_SC
Orpiment_SC

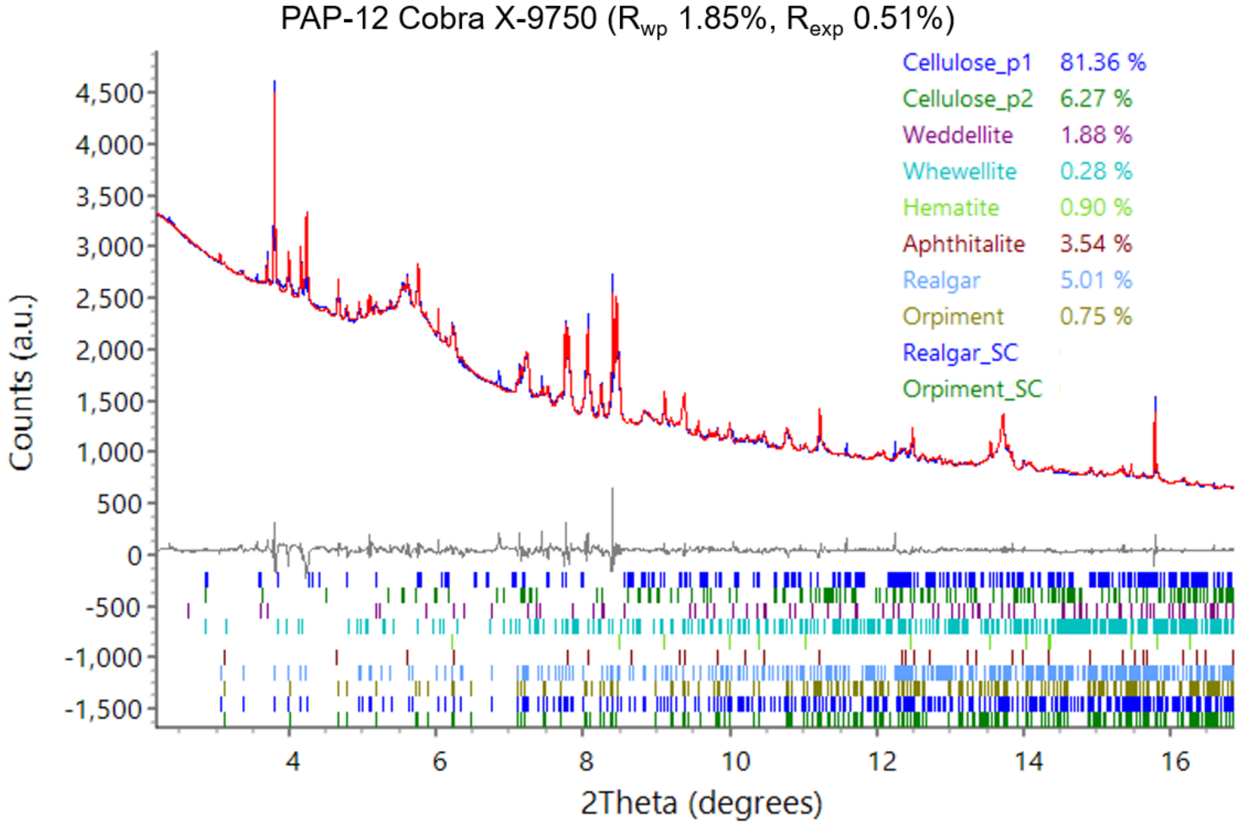
PAP-12 Cobra X-9750 ($R_{wp}$ 1.85%, $R_{exp}$ 0.51%)
Counts (a.u.)
2Theta (degrees)
Cellulose_p1 81.36 %
Cellulose_p2 6.27 %
Weddellite 1.88 %
Whewellite 0.28 %
Hematite 0.90 %
Aphthitalite 3.54 %
Realgar 5.01 %
Orpiment 0.75 %
Realgar_SC
Orpiment_SC

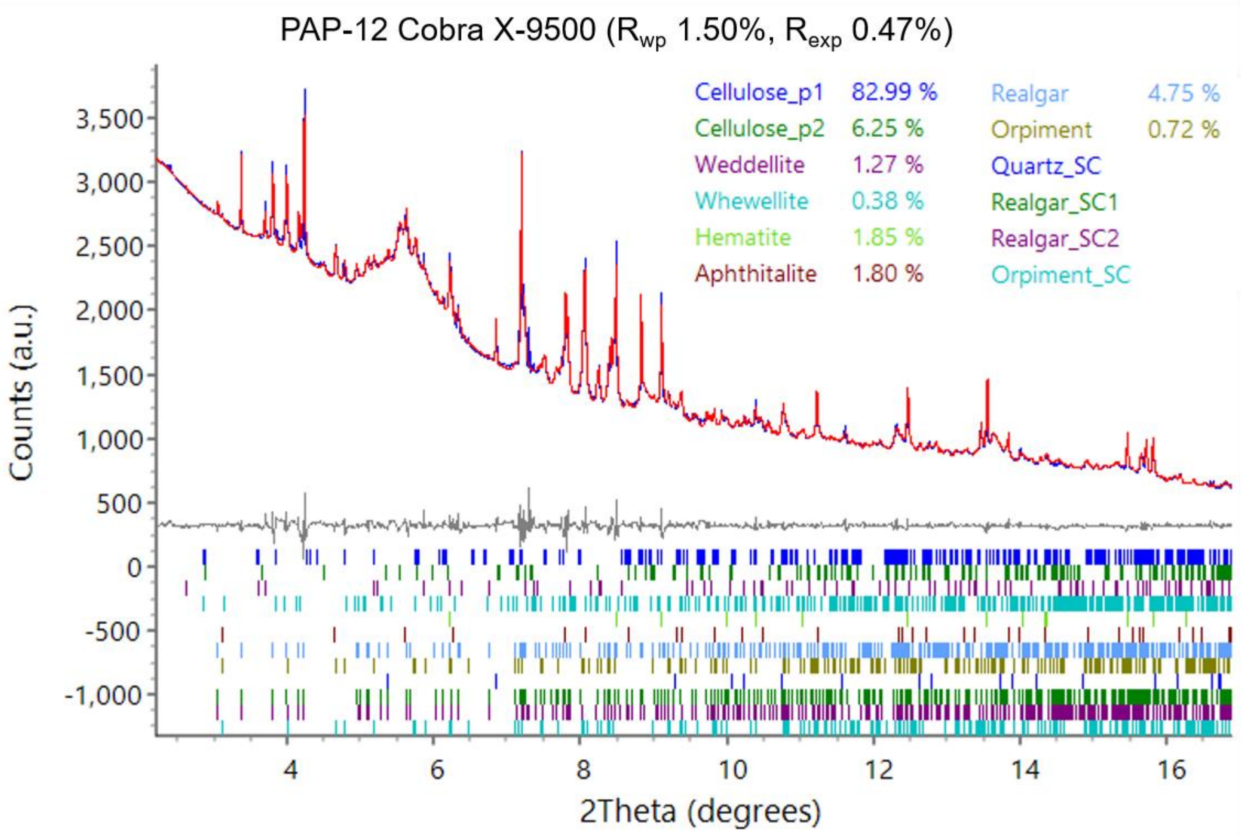
PAP-12 Cobra X-9500 ($R_{wp}$ 1.50%, $R_{exp}$ 0.47%)
Cellulose_p1 82.99 %
Cellulose_p2 6.25 %
Weddellite 1.27 %
Whewellite 0.38 %
Hematite 1.85 %
Aphthitalite 1.80 %
Realgar 4.75 %
Orpiment 0.72 %
Quartz_SC
Realgar_SC1
Realgar_SC2
Orpiment_SC
Counts (a.u.)
3,500
3,000
2,500
2,000
1,500
1,000
500
0
-500
-1,000
4
6
8
10
12
14
16
2Theta (degrees)

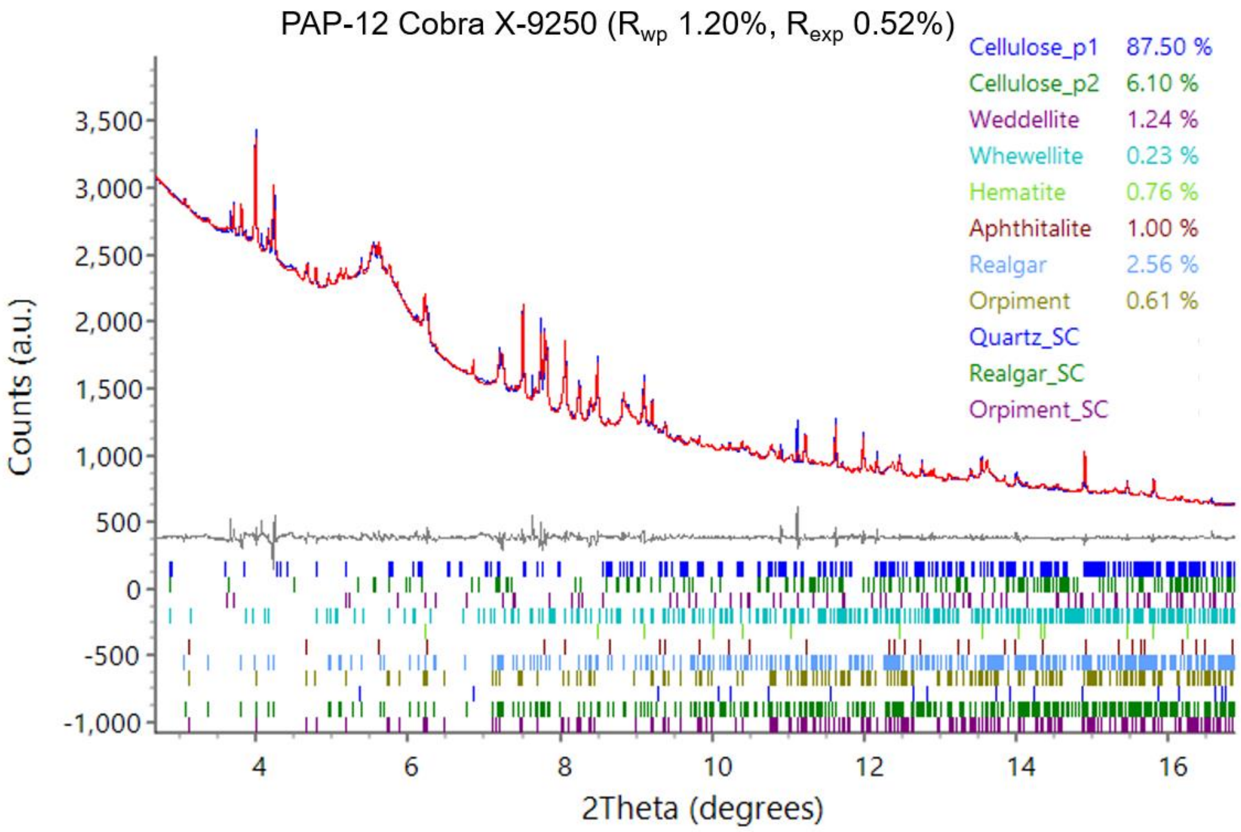
PAP-12 Cobra X-9250 ($R_{wp}$ 1.20%, $R_{exp}$ 0.52%)
Cellulose_p1 87.50 %
Cellulose_p2 6.10 %
Weddellite 1.24 %
Whewellite 0.23 %
Hematite 0.76 %
Aphthitalite 1.00 %
Realgar 2.56 %
Orpiment 0.61 %
Quartz_SC
Realgar_SC
Orpiment_SC
Counts (a.u.)
3,500
3,000
2,500
2,000
1,500
1,000
500
0
-500
-1,000
4
6
8
10
12
14
16
2Theta (degrees)

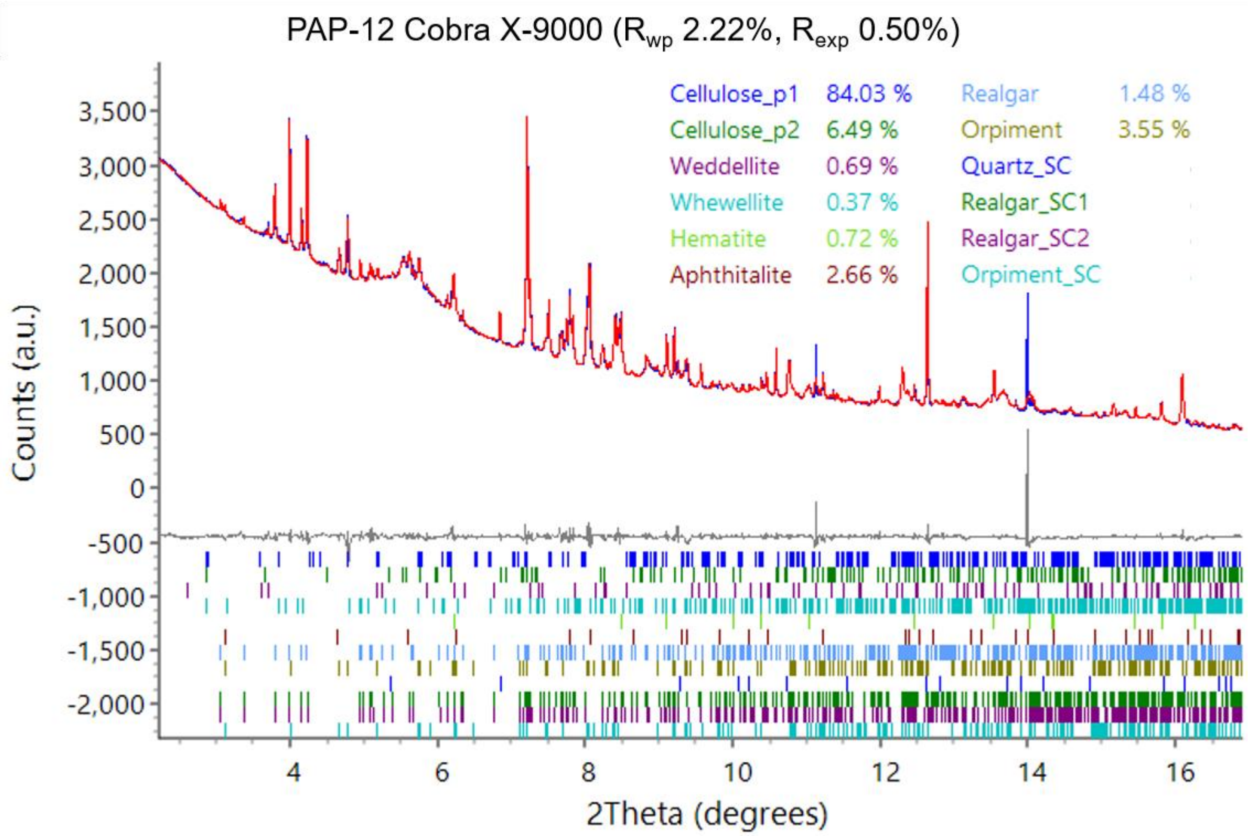
PAP-12 Cobra X-9000 ($R_{wp}$ 2.22%, $R_{exp}$ 0.50%)
Cellulose_p1 84.03 %
Cellulose_p2 6.49 %
Weddellite 0.69 %
Whewellite 0.37 %
Hematite 0.72 %
Aphthitalite 2.66 %
Realgar 1.48 %
Orpiment 3.55 %
Quartz_SC
Realgar_SC1
Realgar_SC2
Orpiment_SC
Counts (a.u.)
2Theta (degrees)

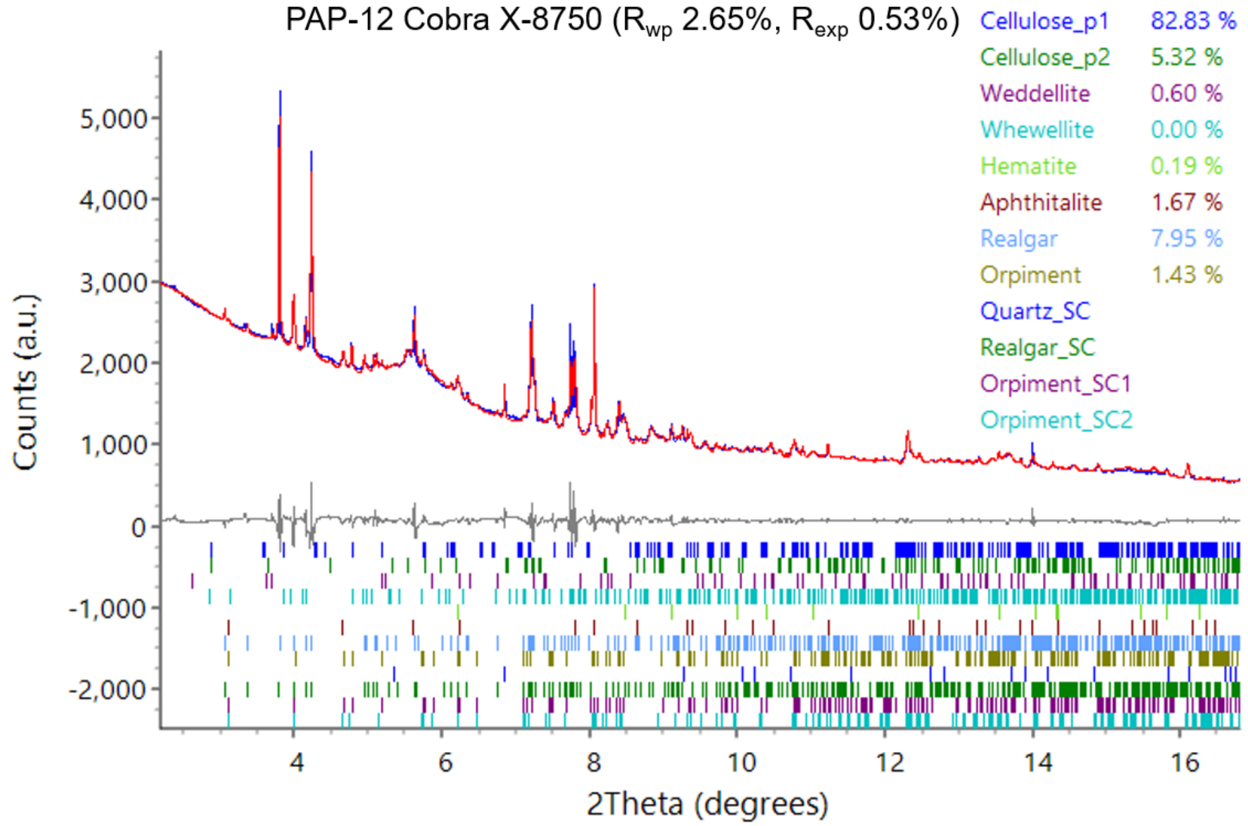
PAP-12 Cobra X-8750 ($R_{wp}$ 2.65%, $R_{exp}$ 0.53%)
Cellulose_p1 82.83 %
Cellulose_p2 5.32 %
Weddellite 0.60 %
Whewellite 0.00 %
Hematite 0.19 %
Aphthitalite 1.67 %
Realgar 7.95 %
Orpiment 1.43 %
Quartz_SC
Realgar_SC
Orpiment_SC1
Orpiment_SC2
Counts (a.u.)
2Theta (degrees)

PAP-12 Cobra X-8500 ($R_{wp}$ 1.42%, $R_{exp}$ 0.55%)

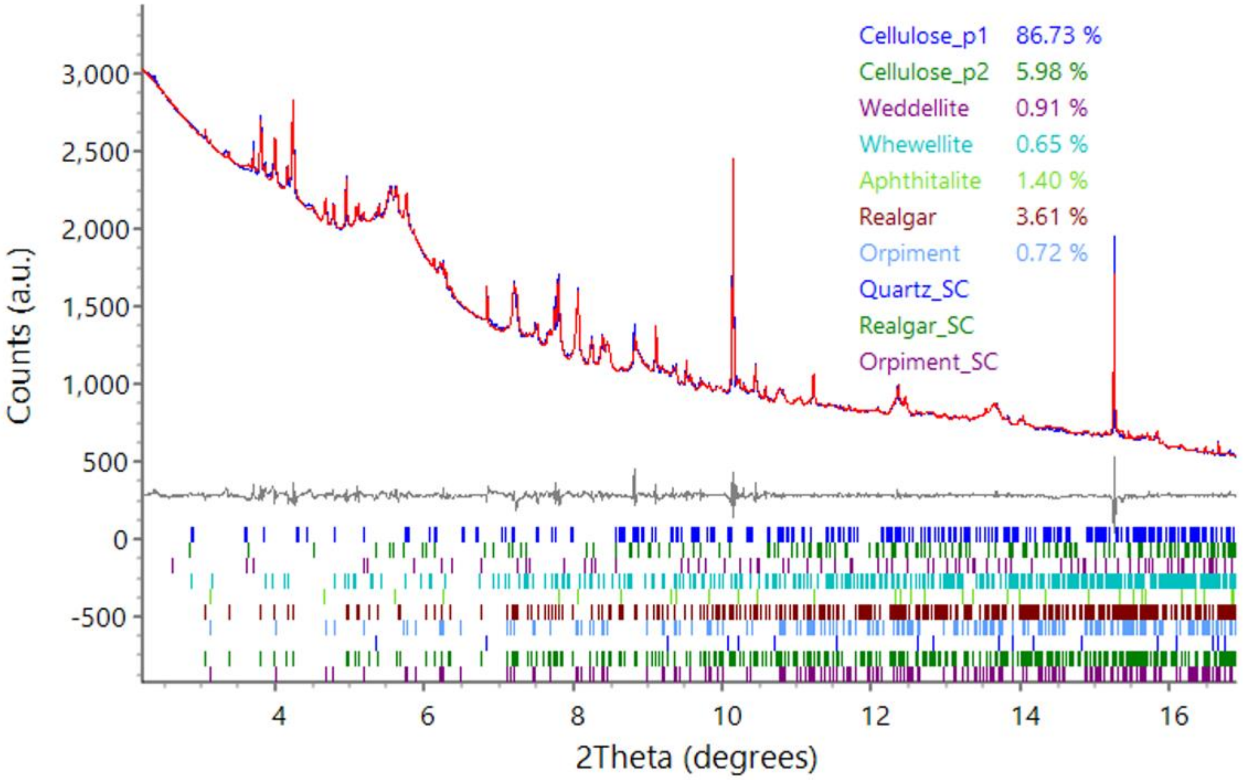


PAP-12 Cobra X-8250 ($R_{wp}$ 1.50%, $R_{exp}$ 0.50%)

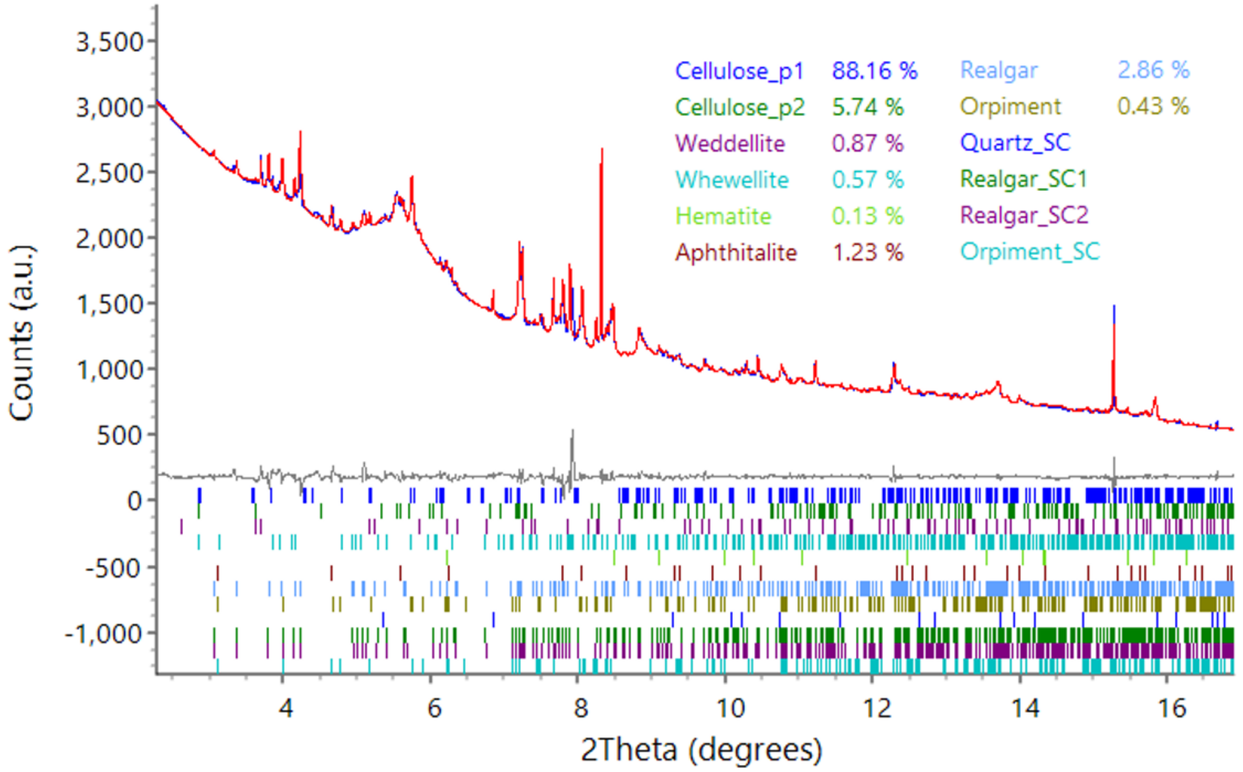

PAP-12 Cobra X-8000 ($R_{wp}$ 1.12%, $R_{exp}$ 0.48%)

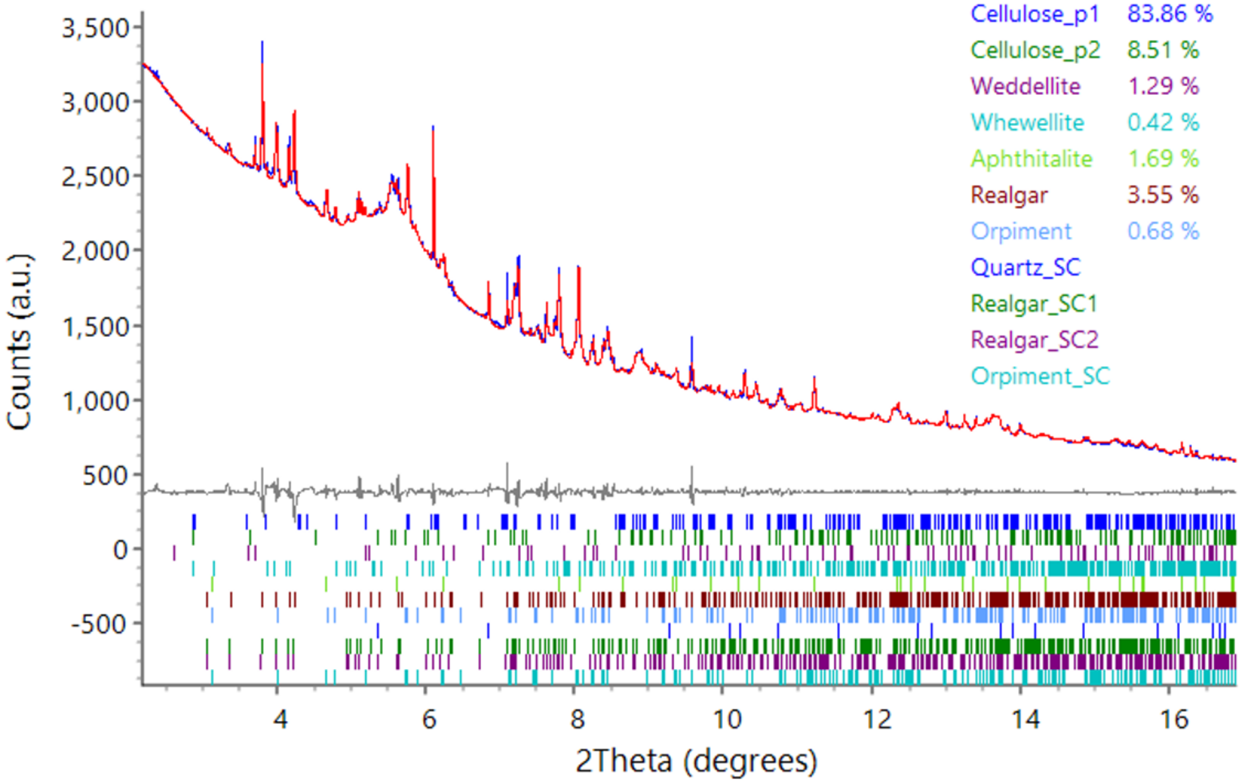


PAP-12 Cobra X-7750 ($R_{wp}$ 1.18%, $R_{exp}$ 0.55%)

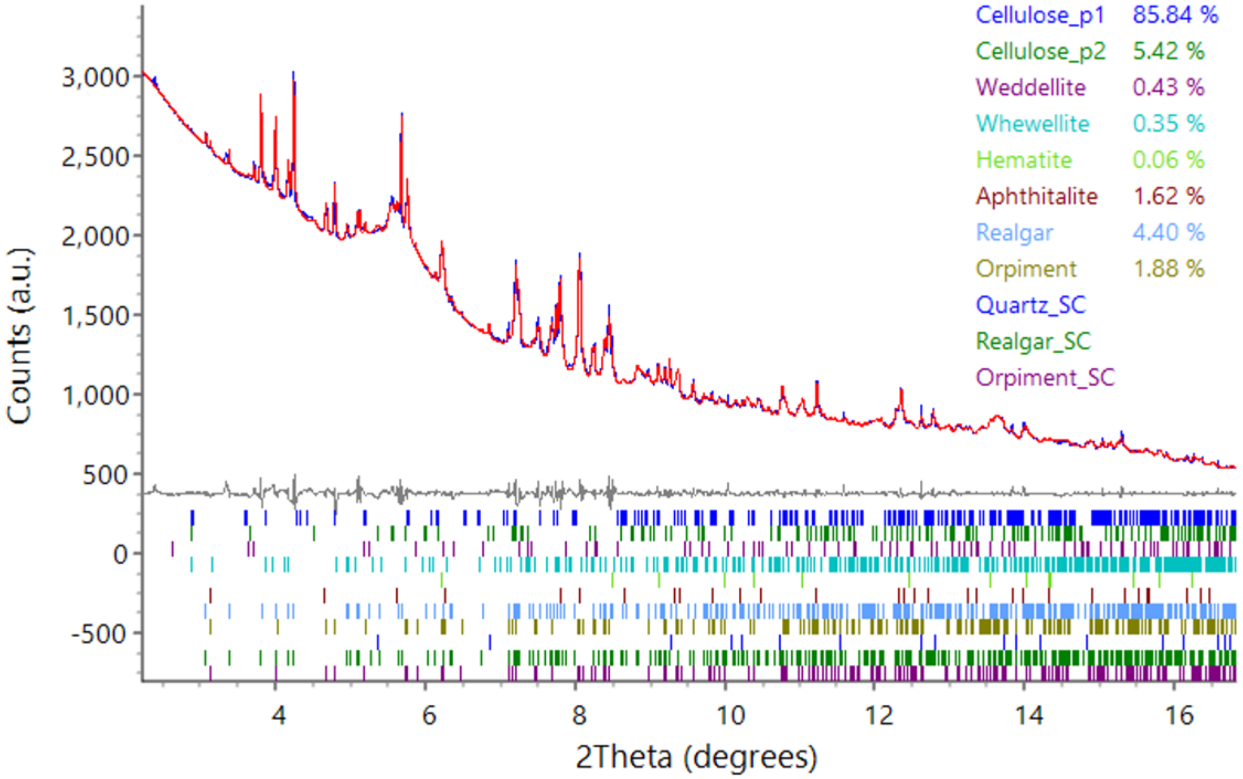

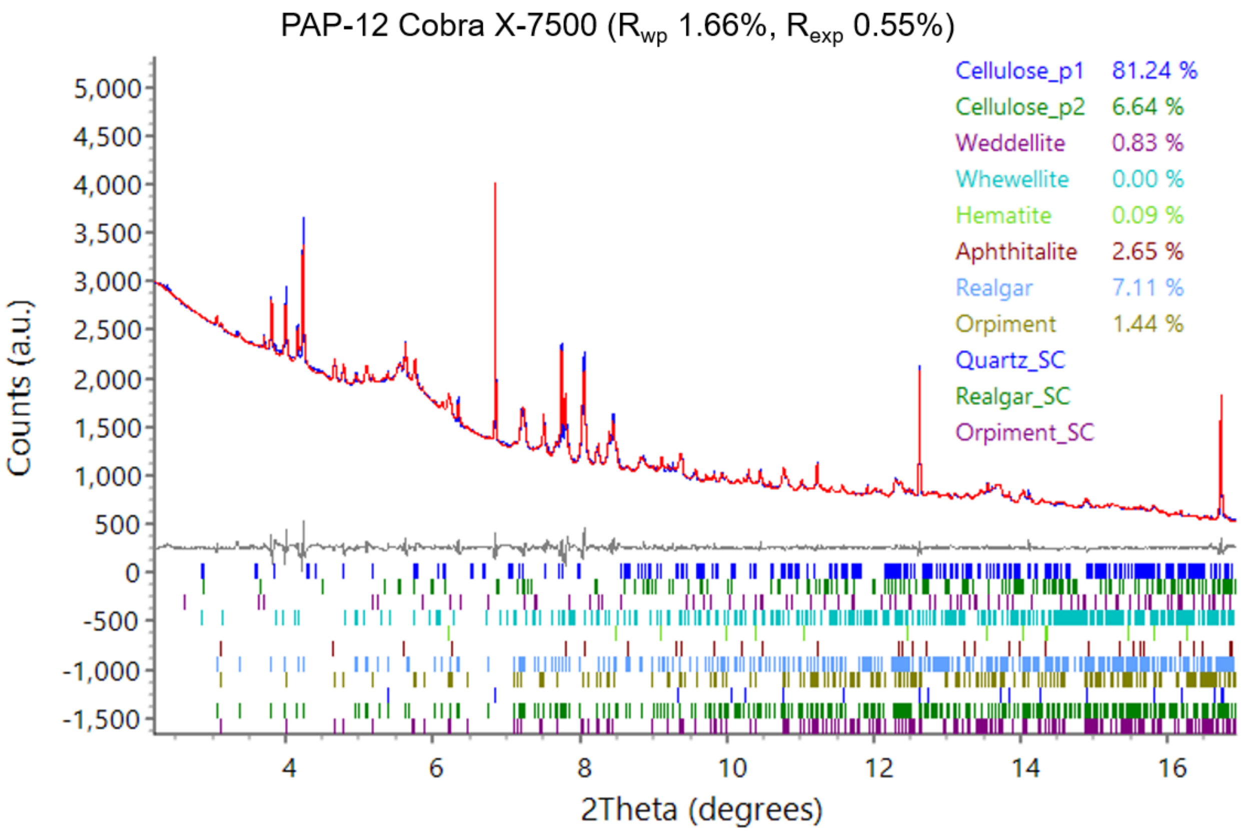
PAP-12 Cobra X-7500 (Rwp 1.66%, Rexp 0.55%)
Cellulose_p1 81.24 %
Cellulose_p2 6.64 %
Weddellite 0.83 %
Whewellite 0.00 %
Hematite 0.09 %
Aphthitalite 2.65 %
Realgar 7.11 %
Orpiment 1.44 %
Quartz_SC
Realgar_SC
Orpiment_SC
Counts (a.u.)
2Theta (degrees)

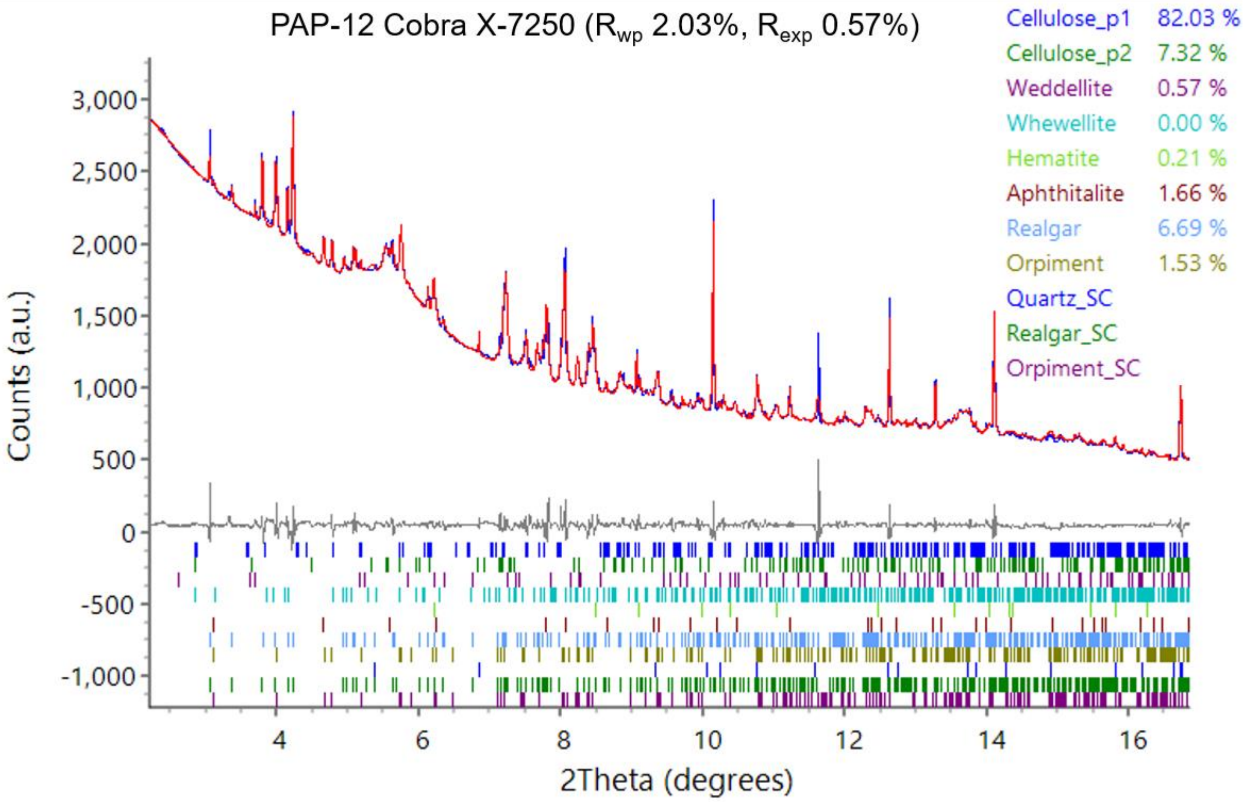
PAP-12 Cobra X-7250 (Rwp 2.03%, Rexp 0.57%)
Cellulose_p1 82.03 %
Cellulose_p2 7.32 %
Weddellite 0.57 %
Whewellite 0.00 %
Hematite 0.21 %
Aphthitalite 1.66 %
Realgar 6.69 %
Orpiment 1.53 %
Quartz_SC
Realgar_SC
Orpiment_SC
Counts (a.u.)
2Theta (degrees)

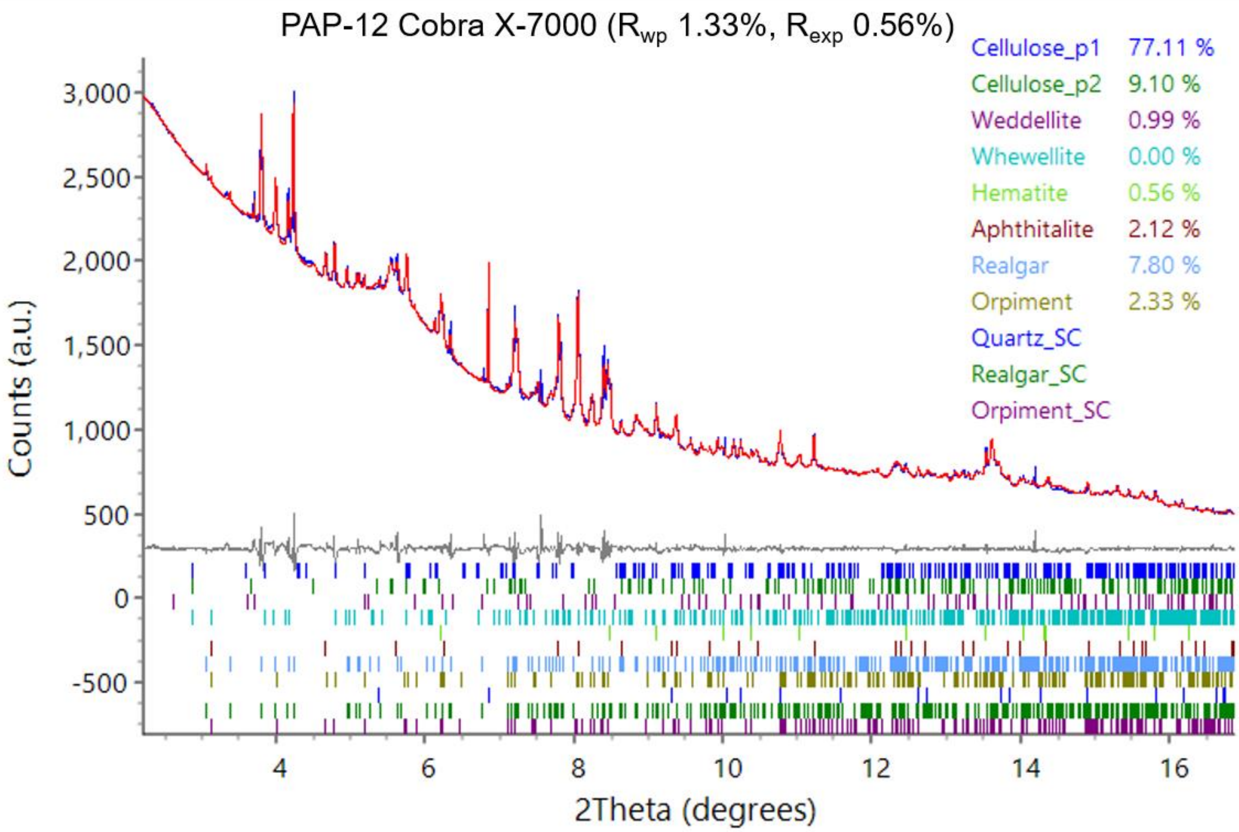
PAP-12 Cobra X-7000 ($R_{wp}$ 1.33%, $R_{exp}$ 0.56%)
Cellulose_p1 77.11 %
Cellulose_p2 9.10 %
Weddellite 0.99 %
Whewellite 0.00 %
Hematite 0.56 %
Aphthitalite 2.12 %
Realgar 7.80 %
Orpiment 2.33 %
Quartz_SC
Realgar_SC
Orpiment_SC
Counts (a.u.)
2Theta (degrees)

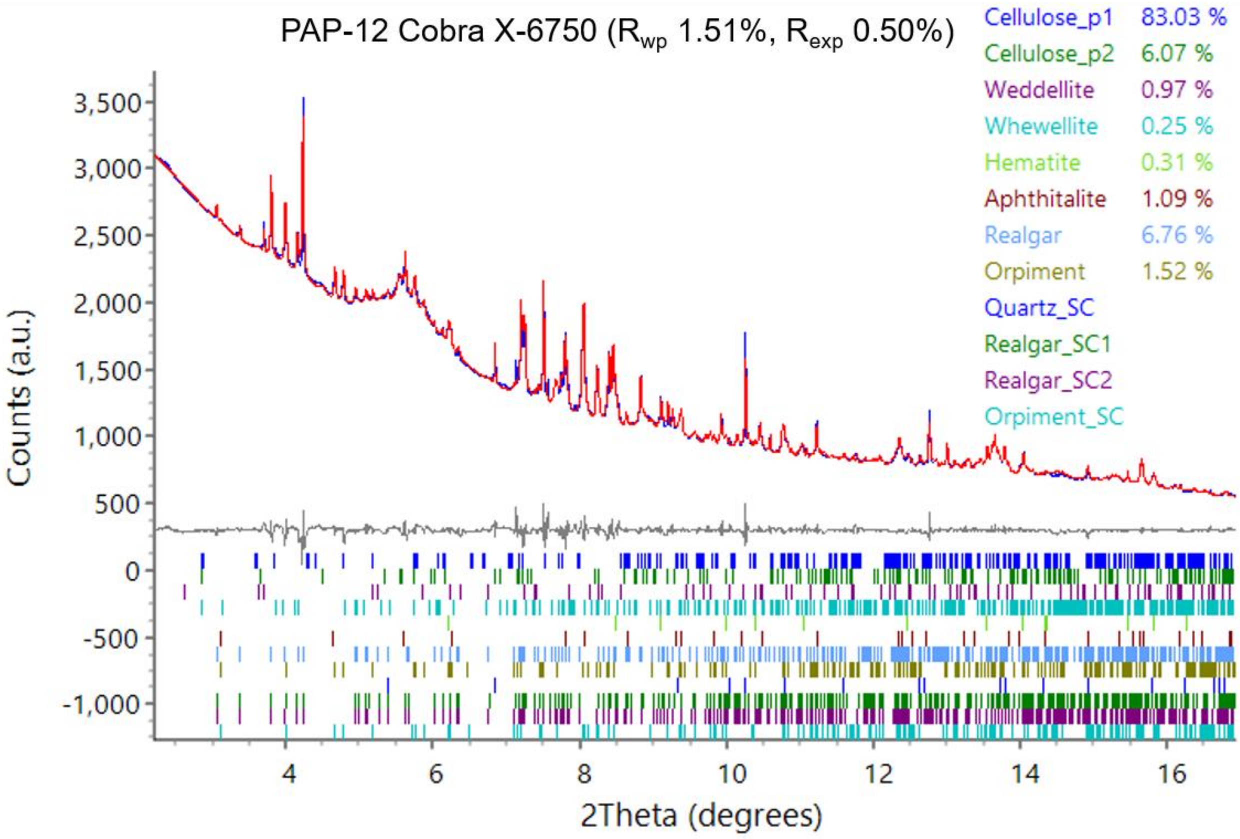
PAP-12 Cobra X-6750 ($R_{wp}$ 1.51%, $R_{exp}$ 0.50%)
Cellulose_p1 83.03 %
Cellulose_p2 6.07 %
Weddellite 0.97 %
Whewellite 0.25 %
Hematite 0.31 %
Aphthitalite 1.09 %
Realgar 6.76 %
Orpiment 1.52 %
Quartz_SC
Realgar_SC1
Realgar_SC2
Orpiment_SC
Counts (a.u.)
2Theta (degrees)

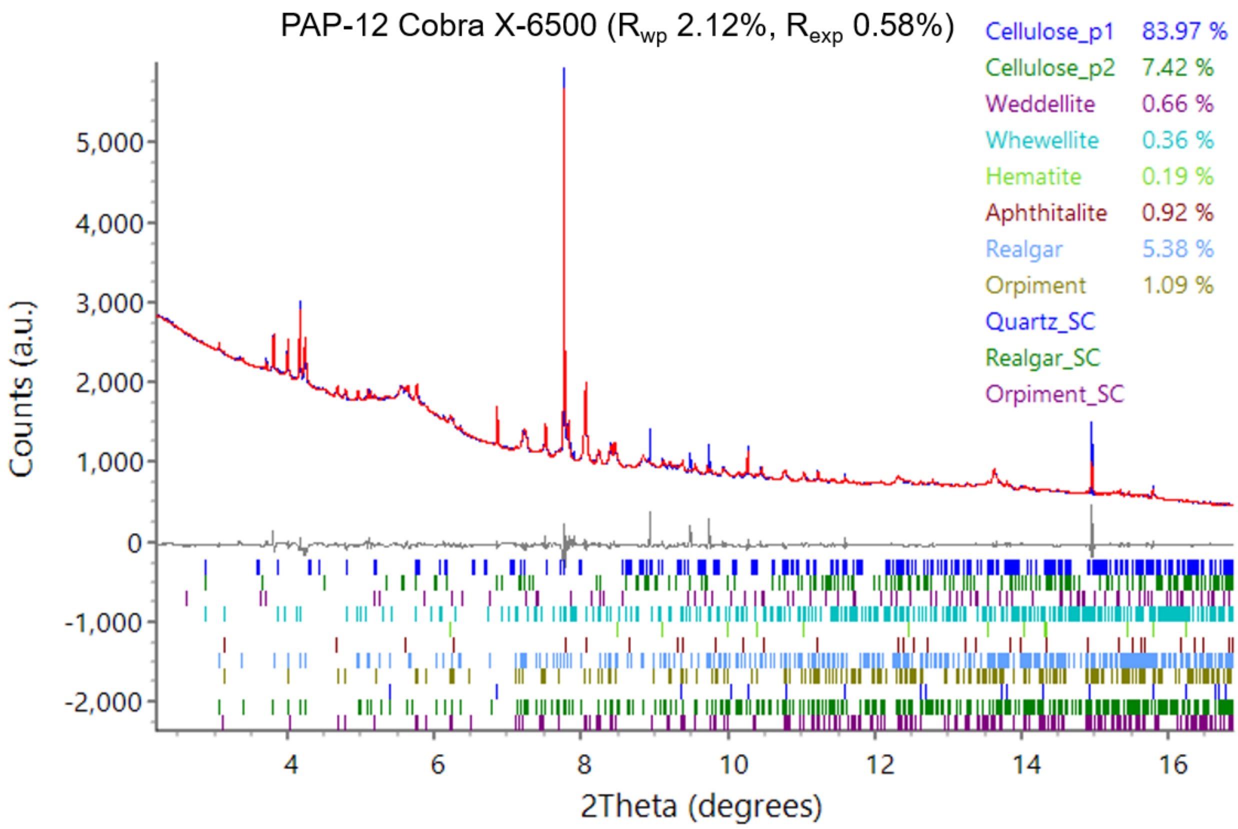
PAP-12 Cobra X-6500 ($R_{wp}$ 2.12%, $R_{exp}$ 0.58%)
Cellulose_p1 83.97 %
Cellulose_p2 7.42 %
Weddellite 0.66 %
Whewellite 0.36 %
Hematite 0.19 %
Aphthitalite 0.92 %
Realgar 5.38 %
Orpiment 1.09 %
Quartz_SC
Realgar_SC
Orpiment_SC
Counts (a.u.)
5,000
4,000
3,000
2,000
1,000
0
-1,000
-2,000
4
6
8
10
12
14
16
2Theta (degrees)
